# Evidence of the Lewis‐Amphoteric Character of Tris(pentafluoroethyl)silanide, [Si(C_2_F_5_)_3_]^−^

**DOI:** 10.1002/anie.202016455

**Published:** 2021-03-22

**Authors:** Natalia Tiessen, Mira Keßler, Beate Neumann, Hans‐Georg Stammler, Berthold Hoge

**Affiliations:** ^1^ Universität Bielefeld Fakultät für Chemie Centrum für Molekulare Materialien Universitätsstrasse 25 33615 Bielefeld Germany

**Keywords:** activation of small molecules, Lewis amphoteric, perfluoroalkyl, silanides, weakly coordinating cations

## Abstract

According to a first view on the geometrical and electronic structure of the tris(pentafluoroethyl)silanide, this anion appears as a Lewis base. Quantum chemical calculations on perfluoroalkylated silanides show significantly lower HOMO and LUMO energy levels in comparison to their non‐fluorinated counterparts, which implies reduced Lewis basicity and increased Lewis acidity of the [Si(C_2_F_5_)_3_]^−^ ion. With these findings and a HOMO–LUMO gap of 4.80 eV similar to N‐heterocyclic silylenes (NHSis), perfluoroalkyl silanides are predestined to exhibit Lewis‐amphoteric character similar to silylenes. Deprotonation of Si(C_2_F_5_)_3_H with sterically demanding phosphazene bases afforded thermally stable phosphazenium salts of the [Si(C_2_F_5_)_3_]^−^ anion, which add to benzaldehyde, benzophenone, CS_2_, and CO_2_ in various manners. This behavior also mirrors the reactivity of silylenes towards ketones as well as heterocumulenes and is rationalized by Lewis amphotericity being inherent in these silanides.

## Graphical abstract

Several room‐temperature‐stable salts of the [Si(C_2_F_5_)_3_]^−^ ion were isolated utilizing sterically demanding phosphazenium cations. Similar to Lewis amphoteric silylenes, the [Si(C_2_F_5_)_3_]^−^ ion activates CO_2_ and adds side‐on to CS_2_ allowing novel negatively charged species like **2** and **3** to be structurally characterized.

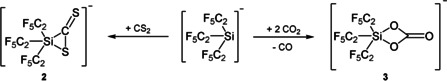

## Introduction

In keeping with the Lewis definition, amphoteric species possess electron‐deficient (Lewis acidic) and electron‐rich (Lewis basic) centers. These reactive positions may be located on two different well separated atoms within one molecule. In a special class of Lewis‐amphoteric systems, the so‐called frustrated Lewis pairs (FLPs), an adduct formation is effectively prevented by sterically demanding substituents, which leads to their inherent capability to activate or incorporate a large variety of small molecules such as H_2_, CO_2_, CS_2_, SO_2_, NO_2_, NO, CO, or unsaturated hydrocarbons.^[1]^

Yet another class of Lewis‐amphoteric species derives from main group IV elements (C, Si, Ge, Sn, and Pb) in the oxidation state +II. Here, the Lewis acidic as well as the Lewis basic position are located at the same center, namely the divalent tetrel atom. The HOMO of these species displays a significant s‐orbital character, whereas the LUMO is usually represented by an empty p‐orbital.^[2]^ It is noteworthy that stable germylenes, stannylenes, and plumbylenes were long known^[3]^ before reports on stable divalent silicon compounds as Si(η^5^‐C_5_Me_5_)_2_ (Jutzi,1986)^[4]^ or the first stable N‐heterocyclic silylene [HC‐N^*t*^Bu]_2_Si (NHSi) (Denk & West, 1994).^[5]^ The latter species are the higher homologues of N‐heterocyclic carbenes (NHCs), the first of which has been presented by Arduengo III in 1991.^[6]^ Denk's seminal discovery stimulated further intense research on silylene chemistry.[^7^, ^8^, ^9^, ^10^, ^11^] Due to their unique Lewis amphoteric properties, silylenes proved to be versatile and valuable building blocks in organosilicon chemistry, reacting readily with multiple bonds as present in alkenes, alkynes, ketones, imines, azides, as well as heteroallenes X=C=Y (X, Y=O, S, NR with R=alkyl). Many of these transformations are initiated by side‐on additions.[^8^, ^9^, ^10^, ^11^, ^12^] It is considered that silylenes with larger bite angles and small HOMO–LUMO gaps are more reactive than those with contrary properties.^[13]^ Thus, for example, acyclic silylenes (HOMO–LUMO gap ca. 2 eV) can even activate H_2_ affording silanes,^[14]^ whereas NHSis cannot (HOMO–LUMO gap ca. 5 eV).^[15]^

**Figure 1:**
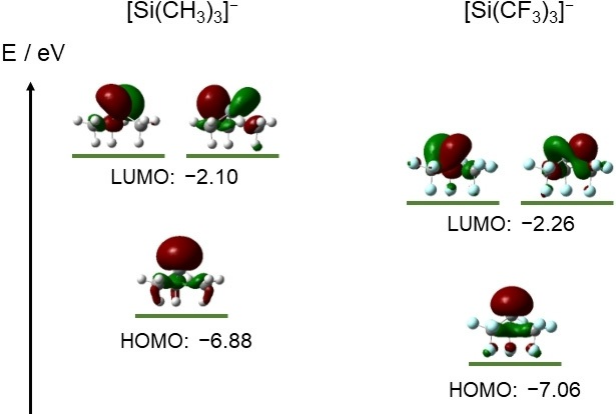
HOMO and LUMO energy levels of [Si(CH_3_)_3_]^−^ and [Si(CF_3_)_3_]^−^ (B3LYP/6‐31+G(3d,p); isosurface value=0.05 e^−^/au^3^).^[16]^

Quantum chemical calculations on the tris(trifluoromethyl)silanide anion, [Si(CF_3_)_3_]^−^, disclose a HOMO–LUMO gap of 4.80 eV as well as lower HOMO and LUMO energy levels (−7.06 eV; −2.26 eV) in comparison to the non‐fluorinated analogue, [Si(CH_3_)_3_]^−^ (−6.88 eV; −2.10 eV) (Figure 1).^[16]^ This is clearly due to the strong electron withdrawing effect of the perfluoroalkyl groups.^[17]^ These computations point to a reduced Lewis basicity and in turn an increased Lewis acidity of the perfluorinated species.

Thus, we were interested in whether or not perfluoroalkyl silanides display Lewis amphoteric character similar to silylenes. To cast some light on this issue we investigated syntheses, structures, and reactivities of salts featuring the [Si(C_2_F_5_)_3_]^−^ ion and support our experimental findings by computational studies.

## Results and Discussion

**Scheme 1:**
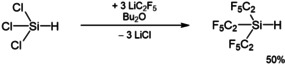
Improved synthesis of Si(C_2_F_5_)_3_H.

While the precursor for the [Si(C_2_F_5_)_3_]^−^ ion, the tris(pentafluoroethyl)silane, Si(C_2_F_5_)_3_H, has been originally synthesized over four steps,^[18]^ an improved and more efficient synthesis is based upon the treatment of SiCl_3_H with three equivalents of in situ generated pentafluoroethyllithium, LiC_2_F_5_, in *n*‐dibutyl ether and subsequent isothermic distillation (Scheme 1).

**Scheme 2:**
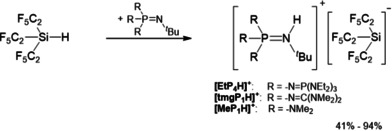
Synthesis of several [R_3_P=N(H)^*t*^Bu][Si(C_2_F_5_)_3_] salts; [MeP_1_H][Si(C_2_F_5_)_3_], [tmgP_1_H][Si(C_2_F_5_)_3_], and [EtP_4_H][Si(C_2_F_5_)_3_].

The most straightforward approach to obtain Li[Si(C_2_F_5_)_3_], the low‐temperature deprotonation of Si(C_2_F_5_)_3_H[^19^, ^20^] by LDA, had to be discarded due to the notorious thermolability of the product at temperatures above −80 °C.[^18^, ^21^] As demonstrated recently, phosphazenium cations, [R_3_P=N(H)^*t*^Bu]^+^ with R being Me_2_N‐ ([MeP_1_H]^+^), (Me_2_N)_2_C=N‐ ([tmgP_1_H]^+^) and (Et_2_N)_3_P=N‐ ([EtP_4_H]^+^), are weakly coordinating and thus predestined to stabilize reactive anions like hydroxide trihydrate [OH(OH_2_)_3_]^−^,^[22]^ silanol silanolates,^[23]^ or the weakly coordinating aluminate [Al(C_2_F_5_)_4_]^−^.^[24]^ Consistently, deprotonation of Si(C_2_F_5_)_3_H with sterically demanding phosphazene bases R_3_P=N^*t*^Bu affords [Si(C_2_F_5_)_3_]^−^ salts in high yields (Scheme 2). The colorless products are sufficiently robust to be handled at room temperature. Elemental analyses confirm their purity (see Supporting Information).

**Figure 2:**
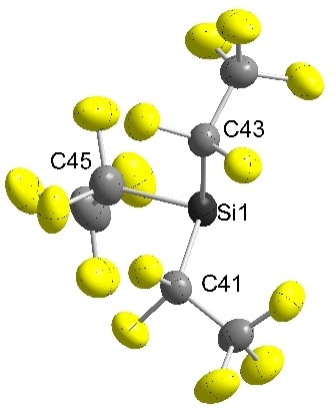
Molecular structure of the non‐disordered anion in [EtP_4_H][Si(C_2_F_5_)_3_] in the solid state.^[45]^ Thermal ellipsoids are represented with a probability of 50 %. The cation has been omitted for clarity. Selected bond lengths [pm] and angles [°] for the [Si(C_2_F_5_)_3_]^−^ moiety: Si1–C41 198.9(3), Si1–C43 199.6(4), Si–C45 199.6(4); C41‐Si1‐C43 94.8(1), C41‐Si1‐C45 95.8(2), C45‐Si1‐C43 94.0(2).

Single crystals of [EtP_4_H][Si(C_2_F_5_)_3_] were grown from a chilled solution in diethyl ether. The salt crystallizes in the triclinic space group *P* $\bar{1}$ with *Z*=4 (Figure 2). One of the two symmetrically independent anions shows some disorder. Due to the particularly weak interaction with the phosphazenium cation, the [Si(C_2_F_5_)_3_]^−^ ion is observed under so‐called pseudo‐gas phase conditions.^[25]^ The C‐Si‐C angles of the [Si(C_2_F_5_)_3_]^−^ ion are significantly smaller (94.0(2)–95.8(2)°) than in the silane Si(C_2_F_5_)_3_H (107.7(3)–108.7(3)°),^[18]^ which indicates a higher p‐orbital character in the Si−C bond and a higher s‐orbital character in the lone pair of the silicon atom.

**Scheme 3:**
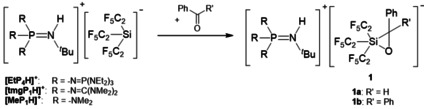
Reaction of [R_3_PN(H)^*t*^Bu][Si(C_2_F_5_)_3_] salts with benzaldehyde and benzophenone.

Analogously to Lewis amphoteric silylenes,[^26^, ^27^, ^28^] the [Si(C_2_F_5_)_3_]^−^ ion undergoes a side‐on addition with carbonyl compounds like benzaldehyde and benzophenone to afford a phosphazenium salt of the corresponding oxasiliranide anion **1 a** and **1 b** (Scheme 3).

^29^Si NMR chemical shifts of pentafluoroethyl‐substituted silicon compounds nicely correlate with the coordination number of the silicon atom and are thus useful in deducing the coordination number of the silicon atom. Thus, tetracoordinated silanes exhibit chemical shifts in the range of +10 to −90 ppm. Penta‐ and hexacoordinated pentafluoroethyl silicon compounds show chemical shifts of −95 to −150 ppm ([SiR_5_]^−^) and −150 to −200 ppm ([SiR_6_]^2−^], respectively.[^19^, ^20^, ^29^] In keeping with this, **1 a** and **1 b** feature a ^29^Si NMR chemical shift of −127.5 ppm and −122.8 ppm, respectively. The ^13^C NMR chemical shift of the R′PhC moiety shifts from 194.0 ppm in benzaldehyde^[30a]^ to 75.5 ppm in **1 a** and from 196.3 ppm in benzophenone^[30b]^ to 78.9 ppm in **1 b**. Negative ESI mass spectra reveal the molecular ion peaks of **1 a** and **1 b** at *m*/*z* values of 491 for [Si(C_2_F_3_)_3_(η^2^‐CPhHO)]^−^ (**1 a**) and 567 for [Si(C_2_F_5_)_3_(η^2^‐CPh_2_O)]^−^ (**1 b**).

**Figure 3:**
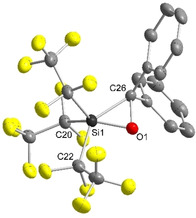
Molecular structure of the non‐disordered anion [Si(C_2_F_5_)_3_(η^2^‐CPh_2_O)]^−^ (**1 b**) in the crystal with [tmgP_1_H]^+^ as counterion.^[45]^ Thermal ellipsoids are represented with a probability of 50 %. The cation and hydrogen atoms have been omitted for clarity. Selected bond lengths [pm] and angles [°]: Si1–O1 165.5(2), C26–O1 152.0(4), Si1–C26 192.6(3); O1‐Si1‐C26 49.5(1), C26‐O1‐Si1 74.6(2), O1‐C26‐Si1 55.9(1).

Single crystals of [tmgP_1_H]**1 b** were grown by slow evaporation of a diethyl ether solution. The phosphazenium salt of [Si(C_2_F_5_)_3_(η^2^‐CPh_2_O)]^−^ (**1 b**) crystallizes in the triclinic space group *P* $\bar{1}$ with *Z*=4; one of the anions is disordered. **1 b** displays the geometry of a highly distorted square pyramid (*τ*=0.18 in the non‐disordered anion and *τ*=0.12 in the disordered one)^[31]^ with a C_2_F_5_ substituent at the apex (Figure 3). The structural motif of a three‐membered Si−O−C heterocycle is quite familiar from the side‐on addition of silylenes to carbonyl compounds.[^26^, ^27^, ^28^] The Si1−O1 and the C26−O1 bond in the three‐membered ring of **1 b** are comparable to those of reported penta‐ and tetracoordinated oxasiliranes. The Si1−C26 bond of **1 b** (192.6(3) pm) is slightly longer than in other penta‐ (185.0–189.2 pm)[^26^, ^27^, ^28^] and tetracoordinated (184.9 pm)^[32]^ oxasiliranes. To the best of our knowledge all pentacoordinated three‐membered Si−O−C‐heterocycles are neutral compounds. Consequently, **1 b** represents the first example of a structurally characterized negatively charged pentacoordinated oxasiliranide of type [SiR_3_(η^2^‐CR′_2_O)]^−^ (R, R′=alkyl, aryl, H).

**Scheme 4:**
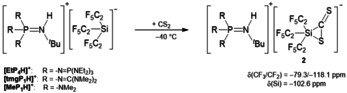
Reaction of [R_3_PN(H)^*t*^Bu][Si(C_2_F_5_)_3_] with CS_2_.

We further investigated the reaction of the [Si(C_2_F_5_)_3_]^−^ ion with CS_2_. When [tmgP_1_H][Si(C_2_F_5_)_3_] is exposed to carbon disulfide at low temperatures, a side‐on addition to CS_2_ takes place. This affords the phosphazenium salt of thiasiliranide **2**, which is only stable at temperatures below −20 °C (Scheme 4).

A ^19^F NMR spectrum of **2** at −20 °C reveals signals at −79.3 ppm and −118.1 ppm for the corresponding CF_3_ and the CF_2_ groups. Consistent with a pentacoordinated silicon atom, **2** features a ^29^Si NMR chemical shift of −102.6 ppm.

**Figure 4:**
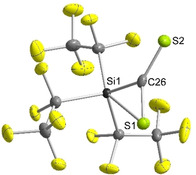
Molecular structure of [Si(C_2_F_5_)_3_(η^2^‐CS_2_)]^−^ (**2**) in the solid state with [tmgP_1_H]^+^ as counterion.^[45]^ Thermal ellipsoids are represented with a probability of 50 %. The cation and minor occupied disordered C_2_F_5_ groups have been omitted for clarity. Selected bond lengths [pm] and angles [°]: Si1–S1 266.0(1), Si1–C26 184.3(2), C26–S1 169.6(2), C26–S2 165.3(2); C26‐Si1‐S1 39.2(1), Si1‐S1‐C26 43.4(1), Si1‐C26‐S1 97.4(1), Si1‐C26‐S2 131.1(1), S1‐C26‐S2 131.5(1).

**Figure 5:**
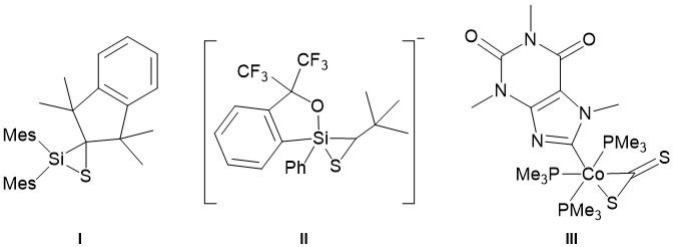
Thiasilirane **I**, thiasiliranide ion **II**, and η^2^‐CS_2_ complex **III**.

When [tmgP_1_H][Si(C_2_F_5_)_3_(η^2^‐CS_2_)] was generated in situ at −40 °C and *n*‐hexane was slowly diffused into the solution, single crystals were obtained, which were prepared at low temperatures for X‐ray diffraction analysis. [tmgP_1_H][Si(C_2_F_5_)_3_(η^2^‐CS_2_)] crystallizes in the monoclinic space group *P*2_1_/*n*; two C_2_F_5_ groups are disordered over two sites. Thiasiliranide **2** exhibits a highly distorted geometry with rather a trigonal‐bipyramidal than a square‐pyramidal geometry (*τ*=0.51, Figure 4). The Si1−S1 bond (266.0(1) pm) is significantly longer than in tetracoordinated thiasilirane **I** (209.3 pm)^[33]^ and 10 pm longer than in pentacoordinated thiasiliranide ion **II** (256.9(9) pm, Figure 5).^[34]^ However, the Si1−S1 bond is much shorter than the sum of the van der Waals radii (390 pm),^[35]^ which implies some degree of silicon−sulfur bonding. Surprisingly, the C26−S2 bond (165.3(2) pm) is only about 4 pm shorter than the C26−S1 bond (169.6(2) pm) and both are in the range of typical C=S double bonds (ca. 167 pm).^[36]^ This bond situation is also observed in η^2^‐CS_2_ transition metal complexes (e.g. 163.3 and 169.3 pm in **III**).^[37]^ In comparison, C=S bonds in crystallized CS_2_ are about 154 pm.^[38]^

**Figure 6:**
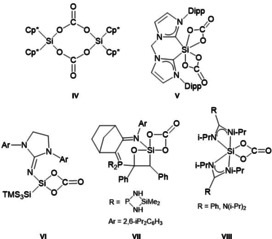
Examples of known silicon carbonates.

Since CO_2_ is one of the main greenhouse gases known, enormous efforts have been made to capture, store, and activate CO_2_ for the synthesis of value‐added products. Apart from transition metal complexes,^[39]^ some silylenes are known to successfully activate CO_2_.^[40]^ Most silylenes form dimeric species like, for example, **IV**,^[41]^ whereas only a few of them lead to compounds like **V**–**VIII** with chelating carbonate ligands (Figure 6).^[42]^

**Scheme 5:**
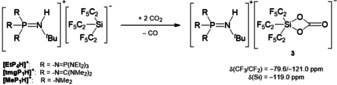
Reaction of [R_3_PN(H)^*t*^Bu][Si(C_2_F_5_)_3_] with CO_2_.

When a [Si(C_2_F_5_)_3_]^−^ salt is treated with an excess of CO_2_, the corresponding salt of silicon carbonate **3** is formed (Scheme 5). The formation of CO was monitored by IR spectroscopy of the gas phase.

The ^19^F NMR spectrum of **3** reveals signals at −79.6 ppm and −121.0 ppm resulting from the CF_3_ and CF_2_ groups. In accordance with a pentacoordinated silicon center, **3** features a resonance at −119.0 ppm in the ^29^Si NMR spectrum. The carbonyl carbon atom resonates in the ^13^C NMR spectrum at 148.6 ppm. In the IR spectrum, the $\tilde{\nu }$ (C=O) mode is detected at 1703 cm^−1^.

**Figure 7:**
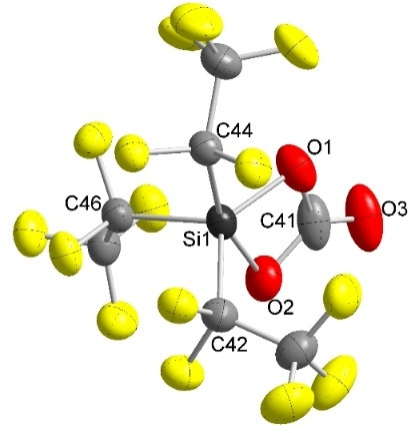
Molecular structure of the [Si(C_2_F_5_)_3_(η^2^‐CO_3_)]^−^ anion (**3**) in the solid state with [EtP_4_H]^+^ as counterion.^[45]^ Thermal ellipsoids are represented with a probability of 50 %. The cation has been omitted for clarity. Selected bond lengths [pm] and angles [°]: Si1–O1 170.1(2), Si1–O2 181.5(2), C41–O3 119.1(4); O1‐Si1‐O2 73.8(1), O2‐C41‐O1 103.6(3), O3‐C41‐O1 126.5(4), O2‐C41‐O3 129.8(4).

Upon evaporation of the solvent, decomposition of **3** to [(F_5_C_2_)CO_2_]^−^ and other unidentified species occurs. Nevertheless single crystals of [EtP_4_H][Si(C_2_F_5_)_3_(η^2^‐CO_3_)] were obtained by diffusion of *n*‐heptane in a Et_2_O/THF solution of the silicon carbonate salt at −40 °C. [EtP_4_H][Si(C_2_F_5_)_3_(η^2^‐CO_3_)] crystallizes in the monoclinic space group *P*2_1_/*c* (Figure 7). Silicon carbonate **3** exhibits a highly distorted geometry with a slight tendency to a trigonal bipyramid (*τ*=0.53). The Si1−O2 bond (181.5(2) pm) is about 11 pm longer than the Si1−O1 bond (170.1(2) pm). Both Si−O bond lengths are in the range of known silicon carbonates (171.5–180.4 pm).[^40^, ^41^] The C41=O3 bond length is comparable to the ones in other silicon carbonates, underlining its double‐bond character. The sum of the angles about C41 of 359.9° confirms a planar coordination sphere. Though some silicon carbonates are known, to the best of our knowledge **3** represents the first structurally characterized negatively charged silicon carbonate of the type [SiR_3_(η^2^‐CO_3_)]^−^ with exclusively organic substituents R=aryl, alkyl.

## Computational Studies

In order to support our experimental findings, we performed computational studies at B3LYP/6‐31+G(3d,p) level of theory^[16]^ concerning the mechanism of the side‐on addition of the tris(trifluoromethyl)silanide ion, [Si(CF_3_)_3_]^−^, to formaldehyde, benzaldehyde, benzophenone, and CS_2_ as well as of the reaction with CO_2_. To reduce computational cost, electronically similar trifluoromethyl groups instead of pentafluoroethyl groups were used. For the same reason we only investigated the addition of formaldehyde in more detail. For each depicted transition state an intrinsic reaction coordinate calculation was performed to ensure that they indeed connect the correct minima. Further details are given in the Supporting Information.

**Figure 8:**
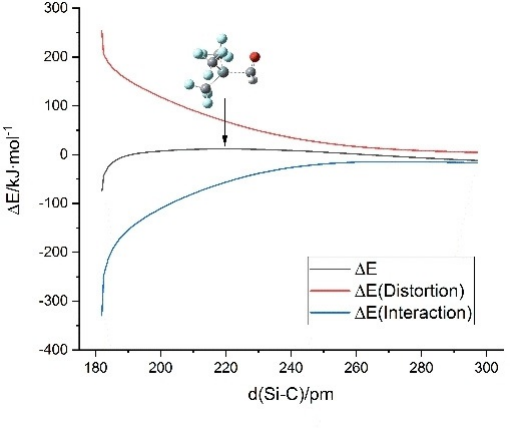
Distortion/interaction diagram of the reaction path of [Si(CF_3_)_3_]^−^+CH_2_O in relation to the Si–C_formaldehyde_ distance. The change in energy is given relative to the starting compounds. The transition state is marked with an arrow.

**Figure 9:**
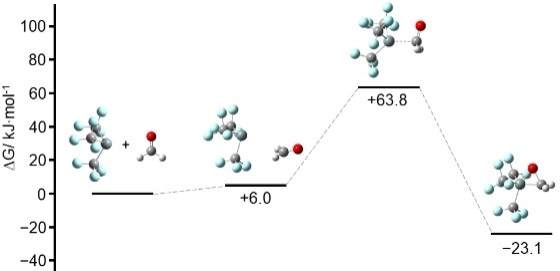
Change in Gibbs free energy along the reaction pathway of [Si(CF_3_)_3_]^−^+CH_2_O.

The addition of formaldehyde to [Si(CF_3_)_3_]^−^ is a concerted process leading to the product [Si(CF_3_)_3_(η^2^‐CH_2_O)]^−^. Figure 8 shows a distortion/interaction diagram^[43]^ for the reaction path in relation to the Si–C_formaldehyde_ distance. Unsurprisingly, the energy required for distortion of the reactants increases with decreasing distance. For values above 265 pm the interaction energy between the fragments outweighs the energy required for distortion due to long‐range interactions of the negatively charged silanide and the dipole of formaldehyde. We located a weakly bound complex as a minimum with a Si–C distance of 367 pm and a change in electronic energy (Δ*E*, zero‐point corrected) of −21.3 kJ mol^−1^ relative to the starting compounds. However, the change in Gibbs free energy (Δ*G*, Figure 9) is +6.0 kJ mol^−1^, indicating that this complex has very little influence on the outcome of the reaction.

Furthermore, we performed an NBO analysis^[44]^ of the silanide as well as of the transition state. In [Si(CF_3_)_3_]^−^ the highest Lewis‐NBO is the lone pair at the silicon atom (occupancy: 1.92) and the lowest non‐Lewis‐NBOs are antibonding and located at the Si−C bonds (occupancy: 0.05 each). The transition state is best described as an alcoholate with a Si−C_formaldehyde_ bond. However, the occupancy of the three lone pairs at the oxygen atom is relatively low (1.62, 1.88, and 1.98), whereas the antibonding Si−C_formaldehyde_ (0.33) is relatively highly occupied due to an extremely large stabilization value from delocalization of one lone pair into this antibonding Si−C NBO (256.6 kJ mol^−1^). The antibonding Si−C_trifluoromethyl_ NBOs have higher occupancies than the corresponding ones in [Si(CF_3_)_3_]^−^ (anti‐periplanar to O: 0.15, synclinal to O: 0.08 each). Delocalization of the lone pairs at the oxygen atom into the Si−C antibond in *trans* position has stabilization values of 7.1 kJ mol^−1^ and 3.1 kJ mol^−1^.

**Figure 10:**
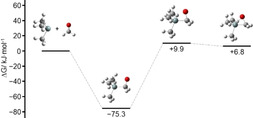
Change in Gibbs free energy along the reaction pathway of [Si(CH_3_)_3_]^−^+CH_2_O.

**Figure 11:**
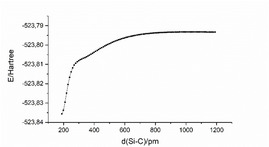
Plot of the electronic energy in relation to the Si–C_formaldehyde_ distance obtained by a relaxed potential energy scan.

In contrast to the perfluorinated silanide, the non‐fluorinated [Si(CH_3_)_3_]^−^ ion attacks only the carbon atom of formaldehyde, forming [Si(CH_3_)_3_(η^1^‐CH_2_O)]^−^ (Figure 10). [Si(CH_3_)_3_(η^2^‐CH_2_O)]^−^ is 82.1 kJ mol^−1^ higher in energy and therefore immaterial to the reaction. Since no transition state between the starting compounds and [Si(CH_3_)_3_(η^1^‐CH_2_O)]^−^ was found, we performed a relaxed potential energy scan along the Si−C_formaldehyde_ bond to gain some insight into the reaction path (Figure 11). Apparently, it is a highly exothermic reaction with no activation barrier.

The addition of [Si(CF_3_)_3_]^−^ to benzaldehyde, benzophenone, and CS_2_ proceeds analogously to the reaction with formaldehyde. The reaction parameters are given in Table 1. The activation barrier of the addition to the bulkier benzaldehyde is higher than of the addition to formaldehyde. The addition to benzophenone has the highest activation barrier. Since the reaction time was by far the longest, this is consistent with the experimental results. The Δ*G* value is positive, which contradicts the experiment, but those values are known to be error‐prone.

**Table 1:** Change in energy (zero‐point corrected) and Gibbs free energy (298.15 K, 1.0 atm) for the transition state (Δ*E* ^≠^ and Δ*G* ^≠^) and the product (Δ*E* and Δ*G*) of the concerted side‐on addition of [Si(CF_3_)_3_]^−^ to formaldehyde, benzaldehyde, benzophenone, and CS_2_.

Substrate	Δ*E* ^≠^(TS) [kJ mol^−1^]	Δ*E*(Product) [kJ mol^−1^]	Δ*G* ^≠^(TS) [kJ mol^−1^]	Δ*G*(Product) [kJ mol^−1^]
CH_2_O	17.9	−71.1	63.8	−23.1
CPhHO	38.2	−59.7	90.7	−4.4
CPh_2_O	58.4	−45.5	115.3	9.9
CS_2_^[a]^	37.2	−52.3	70.0	−7.9

[a] Note that the reaction was carried out at temperatures below −20 °C due to decomposition of the product, but Δ*G* is given for room temperature.

**Figure 12:**
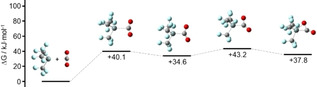
Change in Gibbs free energy along the reaction pathway of [Si(CH_3_)_3_]^−^+CO_2_.

**Figure 13:**
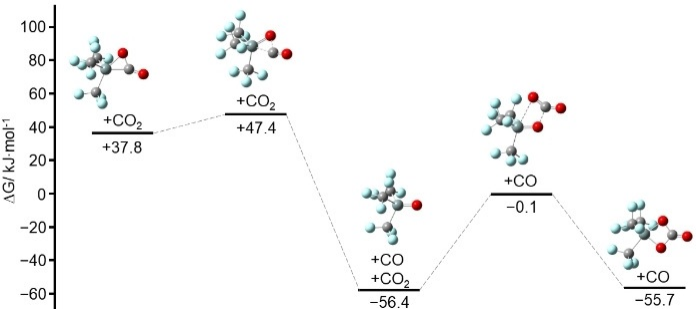
Change in Gibbs free energy along one possible reaction pathway of [Si(CF_3_)_3_(η^2^‐CO_2_)]^−^+CO_2_ relative to the initial educts.

**Figure 14:**
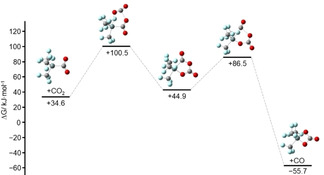
Change in Gibbs free energy along one possible reaction pathway of [Si(CF_3_)_3_(η^1^‐CO_2_)]^−^+CO_2_ relative to the initial starting compounds.

Contrary to the other reactions, CO_2_ does not add side‐on in a concerted mechanism to [Si(CF_3_)_3_]^−^. Stationary points are shown in Figure 12. Although we located two local energy minima along the reaction pathway, [Si(CF_3_)_3_(η^1^‐CO_2_)]^−^ and [Si(CF_3_)_3_(η^2^‐CO_2_)]^−^, Δ*G* is positive in both cases, suggesting that these are not the favored products. Based on the experimental results we studied potential consecutive reactions. One possible reaction starts with the elimination of CO from [Si(CF_3_)_3_(η^2^‐CO_2_)]^−^ followed by the addition of a second equivalent of CO_2_ (Figure 13). However, since [Si(CF_3_)_3_(η^2^‐CO_3_)]^−^ is 0.7 kJ mol^−1^ higher in energy than [Si(CF_3_)_3_O]^−^+CO_2_ this pathway seems not likely. Another possibility starts with the addition of a second equivalent CO_2_ to [Si(CF_3_)_3_(η^1^‐CO_2_)]^−^, forming an intermediate product featuring a five‐membered ring (Figure 14). In this case elimination of CO would lead to the overall thermodynamically favored product [Si(CF_3_)_3_(η^2^‐CO_3_)]^−^.

## Conclusion

**Scheme 6:**
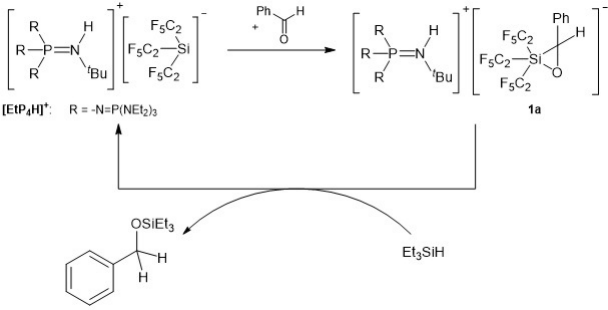
Catalytic hydrosilylation of benzaldehyde by [EtP_4_H][Si(C_2_F_5_)_3_] via [EtP_4_H]**1 a**.

In this Research Article we reported on high yielding (up to 94 %) syntheses of room‐temperature stable [Si(C_2_F_5_)_3_]^−^ salts utilizing phosphazenium cations, which as weakly coordinating cations stabilize the reactive anion. This allows the structural characterization of the anion under so‐called pseudo‐gas phase conditions. Most importantly, while exploring the reactivity of the [Si(C_2_F_5_)_3_]^−^ ion we disclosed its formal Lewis amphoteric behavior. Just like silylenes, the anion displays Lewis basic and Lewis acidic character. This is evident by the side‐on additions to benzaldehyde, benzophenone, and CS_2_, as well as by the activation of CO_2_. We isolated and structurally characterized novel negatively charged species like oxasiliranide **1 b**, thiasiliranide **2**, and silicon carbonate **3**. To the best of our knowledge, **1 b** and **3** represent the first examples of structurally characterized negatively charged hypervalent three‐ and four‐membered heterocycles [R_3_Si(η^2^‐CR′_2_O)]^−^ and [R_3_Si(η^2^‐CO_3_)]^−^ (R, R^′^=alkyl, aryl, H) with organic substituents. Preliminary investigations show that the tris(pentafluoroethyl)silanide catalyzes hydrosilylation of carbonyl compounds like benzaldehyde with triethylsilane via the herein isolated oxasiliranide **1 a** (Scheme 6). Detailed studies concerning this hydrosilylation and the activation of other small molecules by [Si(C_2_F_5_)_3_]^−^ are in progress.

## Conflict of interest

The authors declare no conflict of interest.

## Supporting information

As a service to our authors and readers, this journal provides supporting information supplied by the authors. Such materials are peer reviewed and may be re‐organized for online delivery, but are not copy‐edited or typeset. Technical support issues arising from supporting information (other than missing files) should be addressed to the authors.

Supplementary
