# Supplementary material for: Evidence of the Lewis‐Amphoteric Character of Tris(pentafluoroethyl)silanide, [Si(C2F5)3]−
Source: Angew Chem Int Ed Engl. 2021 Mar 22;60(21):12124–31. doi: 10.1002/anie.202016455 (PMC8252080; doi:10.1002/anie.202016455)
Supplement: Supplementary file 1 — Supplementary [file ANIE-60-12124-s001.pdf]

Angewandte Chemie
Eine Zeitschrift der Gesellschaft Deutscher Chemiker

Supporting Information

Evidence of the Lewis-Amphoteric Character of Tris(pentafluoroethyl)silanide, $[Si(C_2F_5)_3]^-$

*Natalia Tiessen, Mira Keßler, Beate Neumann, Hans-Georg Stammler, and Berthold Hoge**

anie_202016455_sm_miscellaneous_information.pdf

Table of Contents

1 Experimental Procedures
1.1 General Part
1.2 Syntheses
1.2.1 Improved synthesis of $Si(C_2F_5)_3H$
1.2.2 General procedure for the synthesis of $[Si(C_2F_5)_3]^-$ salts
1.2.3a Synthesis of $[EtP_4H][Si(C_2F_5)_3(\eta^2\text{-}CPhHO)]$
1.2.3b Synthesis of $[EtP_4H][Si(C_2F_5)_3(\eta^2\text{-}CPh_2O)]$
1.2.4 Synthesis of $[EtP_4H][Si(C_2F_5)_3(\eta^2\text{-}CS_2)]$
1.2.5 Synthesis of $[EtP_4H][Si(C_2F_5)_3(\eta^2\text{-}CO_3)]$
1.2.6 Catalytic hydrosilylation of benzaldehyde

2 Results
2.1 NMR spectra of $Si(C_2F_5)_3H$
2.2 NMR spectra of $[Si(C_2F_5)_3]^-$ salts
2.3 NMR spectra of $[EtP_4H][Si(C_2F_5)_3(\eta^2\text{-}CPhHO)]$
2.4 NMR spectra $[EtP_4H][Si(C_2F_5)_3(\eta^2\text{-}CPh_2O)]$
2.5 NMR spectra of $[tmgP_1H][Si(C_2F_5)_3(\eta^2\text{-}CS_2)]$
2.6 NMR spectra of $[EtP_4H][Si(C_2F_5)_3(\eta^2\text{-}CO_3)]$
2.7 NMR spectra concerning the catalytic hydrosilylation of benzaldehyde
2.8 DFT calculations
2.9 X-ray data

1 Experimental Procedures

1.1 General Part

All reactions were performed in the absence of water and air by use of standard *Schlenk* techniques. Solvents were dried according to usual procedures. Other chemicals were obtained from commercial sources and used without further purification.

NMR spectra were either recorded on a *Bruker Avance III 300* or *Bruker Avance III 500 HD* in the indicated solvent with acetone-[d_6] as lock substance in a capillary or in the indicated deuterated solvent. Positive shifts are downfield from the external standards (TMS for ^{1}H, ^{13}C and ^{29}Si, H_3PO_4 for ^{31}P, CCl_3F for ^{19}F). IR spectroscopic measurements were performed on a *Bruker Alpha-FT-IR* spectrometer with a diamond crystal. Nano-ESI mass spectra were recorded using an *Esquire 3000* ion trap mass spectrometer (*Bruker Daltonik GmbH*, Bremen, Germany) equipped with a nanoESI source. Samples were dissolved in the specified solvent and introduced by static nano ESI utilizing in-house pulled glass emitters. Nitrogen served both as nebulizer gas and dry gas. It was generated by a *Bruker* nitrogen generator *NGM 11*. The mass axis was externally calibrated with *ESI-L Tuning Mix* (*Agilent Technologies*, Santa Clara, CA, USA) as calibration standard. The spectra were recorded with the *Bruker Daltonik esquireNT 5.2 esquireControl* software and *DataAnalysisTM software 3.4* was used for processing the spectra. The melting point was measured on a *Mettler Toledo Mp70* Melting Point System. Elemental analyses were either performed with a *HEKAtech Euro EA 3000* apparatus or by Mikoanalytisches Laboratorium Kolbe (Oberhausen, Germany). SCXRD was performed on a Rigaku Supernova diffractometer.

1.2 Syntheses

1.2.1 Improved synthesis of $Si(C_2F_5)_3H$

n-Butyllithium (1.6 M solution in *n*-hexane, 40.4 g, 94.9 mmol) was freed from *n*-hexane and dissolved in chilled di-*n*-butyl ether (250 mL). The solution was degassed and HC_2F_5 (104 mmol) was condensed at −80 °C onto the mixture. The solution was then stirred for 1 h to reach a temperature of −60 °C. $SiCl_3H$ (4.37 g, 32.3 mmol) was added and the reaction mixture was slowly warmed to ambient temperature. All volatile compounds were separated in vacuum from the reaction mixture. $Si(C_2F_5)_3H$ (6.45 g, 16.7 mmol, 52 %) was obtained by isothermic distillation (−47 °C, 1 – 2 mbar) of the condensate as a colorless liquid in a purity of 96 %. Vapor pressure: 80 mbar at rt.

^{1}H NMR (500.2 MHz, neat, rt): δ = 5.05 (m, $Si(C_2F_5)_3$**H**);

^{19}F NMR ($CDCl_3$, rt): δ = −84.3 (m, 9 F, CF_3), −122.2 ppm (m, 6 F, CF_2).

1.2.2 General procedure for the synthesis of $[Si(C_2F_5)_3]^-$ salts

A solution of the respective base in *n*-hexane was treated with $Si(C_2F_5)_3H$ at −20 °C, whereby a colorless solid separated. Removal of the supernatant and washing the residue with *n*-hexane yielded the corresponding $[Si(C_2F_5)_3]^-$ salts.

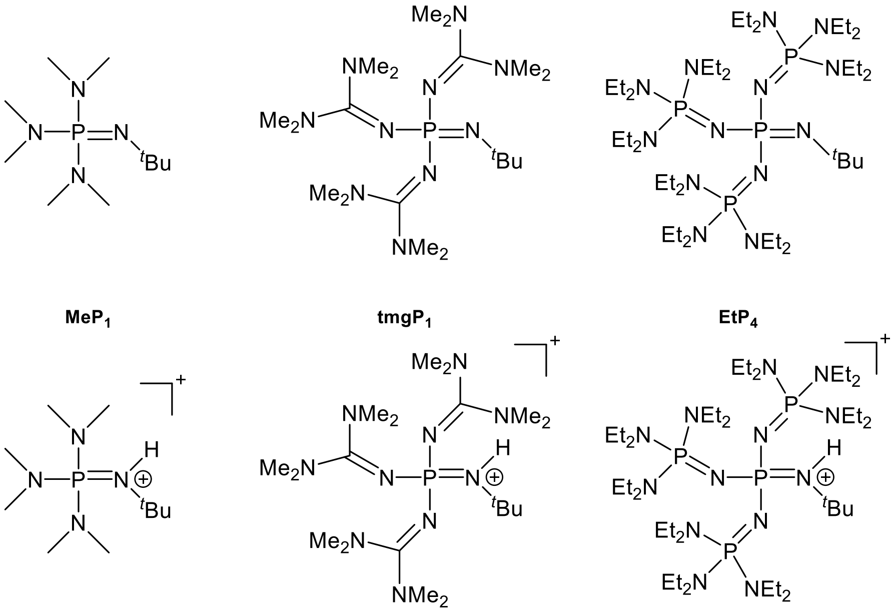

MeP$_1$ **tmgP$_1$** **EtP$_4$**

[MeP$_1$H]$^+$ **[tmgP$_1$H]$^+$** **[EtP$_4$H]$^+$**

Figure S1. Overview of the utilized free and protonated phosphazenebases.

Characterization of the products

$[MeP_1H][Si(C_2F_5)_3]$: MeP_1 (0.506 g, 2.16 mmol) and $Si(C_2F_5)_3H$ (0.869 g, 2.25 mmol) afforded $[MeP_1H][Si(C_2F_3)_3]$ (0.559 g, 0.901 mmol) in 42 % yield.

^{1}H NMR (300.1 MHz, PhCl, rt): δ = 3.15 (d, $^{2}J_{H,P}$ = 8 Hz, 1 H, -P=N(**H**)C(CH$_3$)$_3$), 2.08 (d, $^{3}J_{H,P}$ = 10 Hz,18 H, (C**H**$_3$)$_2$NP-), 0.81 ppm (s, 9 H, -P=N(H)C(C**H**$_3$)$_3$);

^{13}C{^{1}H}DEPTq135 NMR (75.5 MHz, PhCl, rt): δ = 52.5 (s, -P=N(H)**C**(CH$_3$)$_3$), 36.5 (d, $^{2}J_{C,P}$ = 5 Hz, (**C**H$_3$)$_2$NP-), 30.4 ppm (d, $^{3}J_{C,P}$ = 4 Hz, -P=N(H)C(**C**H$_3$)$_3$);

^{13}C{^{19}F}DEPT135 NMR (75.5 MHZ, PhCl, rt): δ = 126.9 (s, CF$_2$), 122.5 ppm (s, CF$_3$);

^{19}F NMR (282.4 MHz, PhCl, rt): δ = −82.1 (m, 9 F, CF$_3$), −115.6 ppm (m, 6 F, CF$_2$);

^{29}Si{^{19}F]DEPT30 (59.6 MHz, PhCl, rt): δ = −35.7 ppm (s, Si);

^{31}P NMR (121.5 MHz, PhCl, rt): δ = 34.1 ppm (m, P=N(H)^{t}Bu);

IR (ATR): $\tilde{\nu}$ = 2960 (w), 2944 (w), 2911 (w), 2866 (w), 2830 (w), 2819 (w), 1489 (w), 1457 (w), 1424 (w), 1395 (w), 1370 (w), 1303 (s), 1178 (vs), 1104 (s), 1090 (s), 1066 (s), 1047 (s), 1034 (s), 990 (vs), 949 (s), 920 (vs), 862 (m), 767 (m), 755 (m), 736 (s), 634 (w), 604 (m), 530 (w), 485 (m), 443 cm^{-1} (m);

MS (ESI, pos., THF): *m/z* (%): 235 (100) $[MeP_1H]^+$; MS (ESI, neg., THF): *m/z* (%): 301 (100) $[Si(C_2F_5)_2FO]^-$, 223 (5) $[Si(C_2F_5)F_4]^-$, 201 (81) $[Si(C_2F_5)F_2O]^-$;

Elemental analysis calcd. (%) for $C_{16}H_{28}F_{15}N_4PSi$: C 30.97, H 4.55, Si 4.53, F 45.93; found: C 30.08, H 4.55, Si 4.50, F 45.79.

[tgmP$_1$H][Si(C$_2$F$_5$)$_3$]: tmgP$_1$ (1.92 g, 4.32 mmol) and Si(C$_2$F$_5$)$_3$H (1.68 g, 4.35 mmol) afforded [tmgP$_1$H][Si(C$_2$F$_3$)$_3$] (3.26 g, 3.93 mmol) in 91 % yield.

^{1}H NMR (CD$_3$CN, rt): δ = 2.83 (s, 36 H, [(C**H**$_3$)$_2$N]$_2$C=N-), 1.94 (d, $^2J_{P,H}$ = 7 Hz, 1 H, -P=N(**H**)^{t}Bu), 1.20 ppm (s, 9 H, -P=N(H)C(C**H**$_3$)$_3$);

^{13}C{^{1}H} IG NMR (75.5 MHz, Et$_2$O, rt): δ = 160.5 (d, $^2J_{C,P}$ = 3 Hz, [(CH$_3$)$_2$N]$_2$**C**=N-), 50.9 (s, -P=N(H)**C**(CH$_3$)$_3$), 40.2 (s, [(**C**H$_3$)$_2$N]$_2$C=N-), 31.4 ppm (d, $^3J_{C,P}$ = 4 Hz, -P=N(H)C(**C**H$_3$)$_3$);

^{13}C{^{19}F}DEPT135 NMR (75.5 MHz, Et$_2$O, rt): δ = 127.4 (s, CF$_2$), 122.5 ppm (s, CF$_3$);

^{19}F NMR (282.4 MHz, Et$_2$O, rt): δ = −82.6 (s, 9 F, CF$_3$), −116.3 ppm (m, 6 F, CF$_2$);

^{29}Si,^{19}F-DEPT30 (59.6 MHz, Et$_2$O, rt): δ = −35.4 ppm (m, Si);

^{31}P NMR (121.5 MHz, Et$_2$O, rt): δ = −12.4 ppm (d, ^2J(^{1}H, ^{31}P) = 7 Hz, -P=N(H)^{t}Bu);

IR (ATR): $\tilde{\nu}$ = 2929 (w), 2900 (w), 2875 (w), 2809 (w), 1694 (w), 1536 (s), 1510 (s), 1473 (m), 1421 (s), 1405 (m), 1379 (s), 1342 (m), 1303 (m), 1192 (s), 1175 (s), 1136 (s), 1101 (s), 1062 (s), 1018 (s), 916 (vs), 897 (vs), 834 (m), 755 (m), 716 (s), 672 (m), 657 (s), 595 (s), 554 (m), 527 (m), 458 (m), 442 (m), 432 (m), 392 cm^{-1} (s);

MS (ESI, pos., THF): *m/z* (%): 445 (100) [tmgP$_1$H]$^+$; MS (ESI, neg., THF): *m/z* (%): 423 (4) [Si(C$_2$F$_5$)$_3$F$_2$]$^-$, 401 (16) [Si(C$_2$F$_5$)$_3$O]$^-$, 323 (32) [Si(C$_2$F$_5$)$_2$F$_3$]$^-$, 301 (23) [Si(C$_2$F$_5$)$_2$FO]$^-$, 223 (23) [Si(C$_2$F$_5$)F$_4$]$^-$, 201 (100) [Si(C$_2$F$_5$)F$_2$O]$^-$.

Elemental analysis calcd. (%) for C$_{25}$H$_{46}$F$_{15}$N$_{10}$PSi: C 36.15, H 5.58, Si 3.38, F 34.30; found: C 36.11, H 5.40, Si 3.41, F 34.17.

[EtP$_4$H][Si(C$_2$F$_5$)$_3$]: EtP$_4$ (1.64 g, 1.85 mmol) and Si(C$_2$F$_5$)$_3$H (0.830 g, 2.15 mmol) afforded [EtP$_4$H][Si(C$_2$F$_3$)$_3$] (2.39 g, 1.88 mmol) in 94 % yield.

^{1}H NMR (300.1 MHz, PhCl, rt): δ = 2.78 (dq, $^3J_{P,H}$ = 10, $^3J_{H,H}$ = 7 Hz, 36 H, [(CH$_3$C**H**$_2$)$_2$N]$_3$P=N-), 1.75 (d, $^2J_{P,H}$ = 7 Hz, 1 H, -P=N(**H**)^{t}Bu), 1.01 (s, 9 H, C(CH$_3$)$_3$), 0.77 ppm (t, $^3J_{H,H}$ = 7 Hz, 54 H, [(C**H**$_3$CH$_2$)$_2$N]$_3$P=N-);

^{13}C{^{1}H} NMR (125.8 MHz, Et$_2$O, rt): δ = 51.2 (d, $^2J_{C,P}$ = 4 Hz, P=N(H)**C**(CH$_3$)$_3$), 39.7 (d, $^2J_{C,P}$ = 6 Hz, [(CH$_3$**C**H$_2$)$_2$N]$_3$P=N-), 31.7 (d, $^3J_{C,P}$ = 5 Hz, -P=N(H)C(**C**H$_3$)$_3$), 13.5 ppm (d, $^3J_{C,P}$ = 4 Hz, [(**C**H$_3$CH$_2$)$_2$N]$_3$P=N-);

^{13}C{^{19}F}DEPT135 NMR (75.5 MHz, Et$_2$O, rt): δ = 127.3 (s, CF$_2$), 122.5 ppm (s, CF$_3$);

^{19}F NMR (470.7 MHz, Et$_2$O rt): δ = −82.6 (m, 9 F, CF$_3$), −116.4 ppm (m, 6 F, CF$_2$);

^{29}Si,^{19}F-DEPT30 (99.4 MHz, Et$_2$O, rt): δ = −35.4 ppm (m, Si);

^{31}P NMR (202.5, PhCl, rt): δ = 7.1 (dm, $^2J_{P,P}$ = 70, $^3J_{P,H}$ = 10 Hz, 3 P, [(Et$_2$N)$_3$**P**=N]$_3$PN(H)^{t}Bu), −34.1 ppm (qd, $^2J_{P,P}$ = 70, $^2J_{P,H}$ = 7 Hz, 1 P, [(Et$_2$N)$_3$P=N]$_3$**P**N(H)^{t}Bu));

IR (ATR): $\tilde{\nu}$ = 2971 (w), 2933 (w), 2872 (w), 1639 (w), 1463 (w), 1378 (m), 1352 (m), 1272 (s, br), 1226 (s), 1200 (vs), 1104 (m), 1056 (m), 1017 (vs), 940 (s), 914 (s), 848 (w), 791 (s), 735 (m), 700 (s), 612 (m), 508 (s), 441 cm^{-1} (s);

MS (ESI, pos., THF): *m/z* (%): 887 (100) [EtP$_4$H]$^+$; MS (ESI, neg., THF): *m/z* (%): 385 (3) [Si(C$_2$F$_5$)$_3$]$^-$, 323 (8) [Si(C$_2$F$_5$)$_2$F$_3$]$^-$, 301 (100) [Si(C$_2$F$_5$)$_2$FO]$^-$, 223 (5) [Si(C$_2$F$_5$)F$_4$]$^-$, 201 (81) [Si(C$_2$F$_5$)F$_2$O]$^-$;

Elemental analysis calcd. (%) for C$_{46}$H$_{100}$F$_{15}$N$_{13}$P$_4$Si: C 43.42, H 7.92, Si 2.21, F 22.40; found: C 42.89, H 7.84, Si 2.17, F 22.18.

1.2.3a Synthesis of $[EtP_4H][Si(C_2F_3)_3(\eta^2\text{-CPhHO})]$

$[EtP_4H][Si(C_2F_3)_3]$ (352 mg, 277 µmol) was solved in chlorobenzene and reacted with benzaldehyde (28.0 µL, 277 µmol) for 3 h at rt. Removal of all volatiles afforded $[EtP_4H][Si(C_2F_3)_3(\eta^2\text{-CPhHO})]$ (375 mg, 272 µmol, 98 %) as a sticky solid.

^{1}H NMR (300.1 MHz, PhCl, rt): δ = 7.43-7.02 (partially overlapped with PhCl, H_{Ar}), 5.01 (s, 1 H, $[(C_2F_5)_3Si(\eta^2\text{-CPh}\mathbf{H}O)]^-$), 2.74 (dq, $^3J_{P,H}$ = 10, $^3J_{H,H}$ = 7 Hz, 36 H, $[(CH_3C\mathbf{H}_2)_2N]_3P{=}N\text{-}$); 1.71 (d, $^2J_{P,H}$ = 7 Hz, 1 H, -P=N(**H**)^{t}Bu), 0.99 (s, 9 H, $C(CH_3)_3$), 0.73 (t, $^3J_{H,H}$ = 7 Hz, 54 H, $[(C\mathbf{H}_3CH_2)_2N]_3P{=}N\text{-}$);

^{13}C{^{1}H}DEPTq135 NMR (125.8 MHz, Et_2O, rt): 147.8 (ipso-C_{Ar}), 127.0 (s, C_{Ar}), 123.2 (s, C_{Ar}), 122.1 (s, C_{Ar}), 75.5 (s, $[(C_2F_5)_3Si(\eta^2\text{-}\mathbf{C}PhHO)]^-$), 50.2 (d, $^2J_{C,P}$ = 4 Hz, P=N(H)**C**$(CH_3)_3$), 38.7 (d, $^2J_{C,P}$ = 6 Hz, $[(CH_3\mathbf{C}H_2)_2N]_3P{=}N\text{-}$), 31.0 (d, $^3J_{C,P}$ = 5 Hz, -P=N(H)C$(\mathbf{C}H_3)_3$), 12.9 ppm(d, $^3J_{C,P}$ = 4 Hz, $[(\mathbf{C}H_3CH_2)_2N]_3P{=}N\text{-}$);

^{13}C{^{19}F}DEPT135 NMR (75.5 MHz, Et_2O, rt): δ = 121.5 (s, CF_3), 119.9 ppm (s, CF_2);

^{19}F NMR (470.7 MHz, Et_2O rt): δ = −82.6 (m, 9 F, CF_3), −123.0 ppm (AB system, 6 F, CF_2);

^{29}Si{^{19}F}DEPT30 (99.4 MHz, Et_2O, rt): δ = −127.5 (s, Si);

^{31}P NMR (202.5, PhCl, rt): 7.0 (dm, $^2J_{P,P}$ = 70, $^3J_{P,H}$ = 10 Hz, 3 P, $[(Et_2N)_3\mathbf{P}{=}N]_3PN(H)^tBu$), −34.2 ppm (qd, $^2J_{P,P}$ = 70, $^2J_{P,H}$ = 7 Hz, 1 P, $[(Et_2N)_3P{=}N]_3\mathbf{P}N(H)^tBu$));

IR (ATR): $\tilde{\nu}$ = 2970 (w), 2933 (w), 2871 (w), 1696 (vw), 1464 (w), 1379 (m), 1351 (m), 1269 (s), 1201 (s), 1173 (vs), 1102 (m), 1055 (m), 1017 (vs), 941 (s), 848 (m), 793 (s), 740 (m), 697 (s), 614 (m), 508 (s), 459 (s), 441 s);

MS (ESI, pos., $CHCl_3$): *m/z* (%): 887 (100) $[EtP_4H]^+$; MS (ESI, neg., $CHCl_3$): *m/z* (%): 491 (100) $[Si(C_2F_3)_3(\eta^2\text{-CPhHO})]^-$, 385 (56) $[Si(C_2F_3)_3]^-$.

1.2.3b Synthesis of $[EtP_4H][Si(C_2F_3)_3(\eta^2\text{-CPh}_2O)]$

$[EtP_4H][Si(C_2F_3)_3]$ (369 mg, 290 µmol) was reacted with benzophenone (381 mg, 2.09 mmol) in chlorobenzene for 16 d at room temperature. The solvent was removed under vacuum and the residue was washed with *n*-pentane (5 x 5 mL). Evaporation to dryness yielded $[EtP_4H][Si(C_2F_3)_3(O)CPh_2]$ (385 mg, 265 µmol, 91 %) as a yellowish solid.

^{1}H NMR (300.1 MHz, PhCl, rt): δ = 7.79-7.19 (partially overlapped with PhCl, m, H_{Ar}), 2.75 (dq, $^3J_{P,H}$ = 10, $^3J_{H,H}$ = 7 Hz, 36 H, $[(CH_3C\mathbf{H}_2)_2N]_3P{=}N\text{-}$); 1.72 (d, $^2J_{P,H}$ = 7 Hz, 1 H, -P=N(**H**)^{t}Bu), 0.99 (s, 9 H, $C(CH_3)_3$), 0.74 ppm (t, $^3J_{H,H}$ = 7 Hz, 54 H, $[(C\mathbf{H}_3CH_2)_2N]_3P{=}N\text{-}$);

^{13}C{^{1}H}-DEPTq135 NMR (125.8 MHz, PhCl, rt): δ = 149.5 (ipso-C_{Ar}), 126.8 (s, C_{Ar}), 125.6 (s, C_{Ar}), 123.3 (s, C_{Ar}), 78.9 (s, $[(C_2F_5)_3Si(\eta^2\text{-}\mathbf{C}Ph_2O)]^-$), 50.2 (d, $^2J_{C,P}$ = 4 Hz, -P=N(H)**C**$(CH_3)_3$), 38.7 (d, $^2J_{C,P}$ = 6 Hz, $[(CH_3\mathbf{C}H_2)_2N]_3P{=}N\text{-}$), 31.0 (d, $^3J_{C,P}$ = 5 Hz, -P=N(H)C$(\mathbf{C}H_3)_3$), 12.9 ppm (d, $^3J_{C,P}$ = 4 Hz, $[(\mathbf{C}H_3CH_2)_2N]_3P{=}N\text{-}$);

^{13}C{^{19}F}DEPT135 NMR (75.5 MHz, PhCl, rt): δ = 121.3 (s, CF_3), 120.1 ppm (s, CF_2);

^{19}F NMR (282.4 MHz, PhCl, rt): δ = −80.4 (s, 9 F, CF_3), −121.2 ppm (s, 6 F, CF_2);

^{29}Si{^{19}F}DEPT30 (59.6 MHz, PhCl, rt): δ = −122.8 ppm (s, Si);

^{29}Si,^{19}F-DEPT30 (59.6 MHz, PhCl, rt): δ = −122.8 ppm (sept, $^2J_{Si,F}$ = 33 Hz, Si);

^{31}P NMR (121.5 MHz, PhCl, rt): δ = 7.0 (dm, $^2J_{P,P}$ = 70, $^3J_{P,H}$ = 10 Hz, 3 P, $[(Et_2N)_3\mathbf{P}{=}N]_3PN(H)^tBu$), −34.2 ppm (qd, $^2J_{P,P}$ = 70, $^2J_{P,H}$ = 7 Hz, 1 P, $[(Et_2N)_3P{=}N]_3\mathbf{P}N(H)^tBu$));

IR (ATR): $\tilde{\nu}$ = 3058 (vw), 2972 (w), 2934 (w), 2872 (w), 1659 (w), 1598 (vw), 1578 (vw), 1545 (vw), 1465 (vw), 1447 (w), 1379 (w), 1351 (m), 1275 (s), 1202 (s), 1174 (vs), 1110 (m), 1055 (m), 1018 (vs), 941 (vs), 919 (m), 847 (m), 794 (m), 764 (m), 742 (m), 697 (vs), 638 (m), 615 (m), 509 (s), 463 (s), 440 (s), 408 cm^{-1} (m);

MS (ESI, pos., ACN): *m/z* (%): 887 (100) $[EtP_4H]^+$; MS (ESI, neg., ACN): *m/z* (%): 567 (100) $[Si(C_2F_5)_3(\eta^2\text{-CPh}_2O)]^-$, 385 (3) $[Si(C_2F_5)_3]^-$;

HRMS (ESI): *m/z* calcd. for $[C_{19}H_{10}OF_{15}Si]^-$: 567.02669 $[M]^-$; found: 567.0262;

1.2.4 Synthesis of [tmgP$_1$H][Si(C$_2$F$_5$)$_3$(η^2-CS$_2$)]

For crystallization [tmgP$_1$H][Si(C$_2$F$_5$)$_3$] was reacted with CS_2 in diethyl ether at −40 °C. *n*-Hexane was slowly diffused into the solution whereby needle-shaped single crystals suitable for X-ray diffraction studies precipitated.

^{19}F NMR (282.4 MHz, Et$_2$O, 253 K): δ = −79.3 (s, 9 F, CF$_3$), −118.1 ppm (s, 6 F, CF$_2$);

^{19}F,^{29}Si HMBC NMR (Et$_2$O, 253 K): δ_{Si} = −102.6 ppm, δ_F = −118.1 ppm.

1.2.5 Synthesis of [EtP$_4$H][Si(C$_2$F$_5$)$_3$(η^2-CO$_3$)]

[EtP$_4$H][Si(C$_2$F$_5$)$_3$] (571 mg, 0.449 mmol) was treated with an excess of CO_2 (2.62 mmol) in chlorobenzene and the reaction mixture was stirred for 10 minutes at room temperature. The gas phase was then investigated by IR spectroscopy. Adding and washing the solution with *n*-pentane yielded [EtP$_4$H][Si(C$_2$F$_5$)$_3$(η^2-CO$_3$)] as a yellowish oil containing residues of solvent. Several attempts to isolate [EtP$_4$H][Si(C$_2$F$_5$)$_3$(η^2-CO$_3$)] led to decomposition, which is why no elemental analysis could be performed.

^{1}H NMR (300.1 MHz, CD$_3$CN, rt): δ = 7.41 – 7.29 (H$_{Ar}$ of PhCl), 3.18 (dq, $^3J_{P,H}$ = 10, $^3J_{H,H}$ = 7 Hz, 36 H, [(CH$_3$C**H**$_2$)$_2$N]$_3$P=N-); 2.10 (d, $^2J_{P,H}$ = 7 Hz, 1 H, -P=N(**H**)^{t}Bu), 1.33 (overlapped with CH$_2$ of *n*-pentane, s, C(CH$_3$)$_3$), 1.11 ppm (t, $^3J_{H,H}$ = 7 Hz, 54 H, [(C**H**$_3$CH$_2$)$_2$N]$_3$P=N-) 0.93 (CH$_3$ of *n*-pentane);

^{13}C{^{1}H}-DEPTq135 NMR (125.8 MHz, CD$_3$CN, rt): δ = 148.6 (s, -**C**O$_3$), 133.8 (*ipso*-C$_{Ar}$ of PhCl), 130.0 (C$_{Ar}$ of PhCl), 128.4 (C$_{Ar}$ of PhCl), 126.7 (C$_{Ar}$ of PhCl), 50.5 (d, $^2J_{C,P}$ = 4 Hz, P=N(H)**C**(CH$_3$)$_3$), 39.0 (d, $^2J_{C,P}$ = 6 Hz, [(CH$_3$**C**H$_2$)$_2$N]$_3$P=N-), 34.9 (CH$_2$ of *n*-pentane), 31.1 (d, $^3J_{C,P}$ = 5 Hz, -P=N(H)C(**C**H$_3$)$_3$), 22.3 (CH$_2$ of *n*-pentane), 13.6 (CH$_3$ of *n*-pentane), 13.0 ppm (d, $^3J_{C,P}$ = 4 Hz, [(**C**H$_3$CH$_2$)$_2$N]$_3$P=N-);

^{13}C{^{19}F}DEPT135 NMR (75.5 MHz, CD$_3$CN, rt): δ = 121.7 (s, CF$_3$), 121.5 ppm (s, CF$_2$);

^{19}F NMR (282.4 MHz, CD$_3$CN, rt): δ = −79.6 (s, 9 F, CF$_3$), −121.0 ppm (s, 6 F, CF$_2$);

^{29}Si{^{19}F}DEPT30 (59.6 MHz, PhCl, rt): δ = −122.8 ppm (s, Si);

^{29}Si,^{19}F-DEPT30 (59.6 MHz, CD$_3$CN, rt): δ = −119.0 ppm (sept, $^2J_{Si,F}$ = 34 Hz, Si);

^{31}P NMR (121.5 MHz, CD$_3$CN, rt): δ = 7.7 (dm, $^2J_{P,P}$ = 70, $^3J_{P,H}$ = 10 Hz, 3 P, [(Et$_2$N)$_3$**P**=N]$_3$PN(H)^{t}Bu), −33.7 ppm (qd, $^2J_{P,P}$ = 70, $^2J_{P,H}$ = 7 Hz, 1 P, [(Et$_2$N)$_3$P=N]$_3$**P**N(H)^{t}Bu));

IR (ATR): $\tilde{\nu}$ = 2972 (w), 2935 (w), 2873 (w), 1703 (w), 1643 (w), 1465 (w), 1380 (m), 1351 (w), 1265 (s), 1202 (s), 1171 (vs), 1114 (m), 1055 (m), 1017 (vs), 941 (s), 920 (m), 849 (w), 793 (m), 739 (w), 700 (s), 616 (m), 592 (w), 504 (s), 440 (s), 407 cm^{-1} (m);

MS (ESI, pos., THF): *m/z* (%): 887 (100) [EtP$_4$H]$^+$; MS (ESI, neg., THF): *m/z* (%): 401 (27) [Si(C$_2$F$_5$)$_3$O]$^-$, 301 (30) [Si(C$_2$F$_5$)$_2$FO]$^-$, 201 (12) [Si(C$_2$F$_5$)F$_2$O]$^-$, 163 (62) [(F$_5$C$_2$)CO$_2$]$^-$, 113 (29) [(F$_3$C)CO$_2$]$^-$.

IR (gas phase): $\tilde{\nu}$ = 3086 (vw, br), 3001 (w), 2143 (w, ν(CO), CO), 1357 (w), 1308 (m), 1219 (vs), 1209 (vs), 1199 (s), 1147 (s), 1142 (s), 874 (w), 867 (w), 727 (w), 721 (w), 585 (w), 572 (w), 521 (w).

1.2.6 Catalytic hydrosilylation of benzaldehyde

In a Young NMR tube [EtP$_4$H][Si(C$_2$F$_5$)$_3$] (20 mg, 16 µmol) and benzaldehyde (21 mg, 198 µmol) were solved in THF. After 3 h triethylsilane (69 mg, 593 µmol) was added. The progress of the reaction was monitored by ^{1}H NMR spectroscopy.

(Benzyloxy)triethylsilane:
^{1}H NMR (500.0 MHz, THF, rt): δ = 7.19-7.03 (AA'XX' system, 5 H, H$_{Ar}$), 4.61 (s, 2 H, PhC**H**$_2$Si(CH$_2$CH$_3$)$_3$), 0.86 (t, partially overlapped with surplus HSiEt$_3$, PhCH$_2$Si(CH$_2$C**H**$_3$)$_3$), 0.54 (q, partially overlapped with surplus HSiEt$_3$, PhCH$_2$Si(C**H**$_2$CH$_3$)$_3$).

2 Results

2.1 NMR spectra of $Si(C_2F_5)_3H$

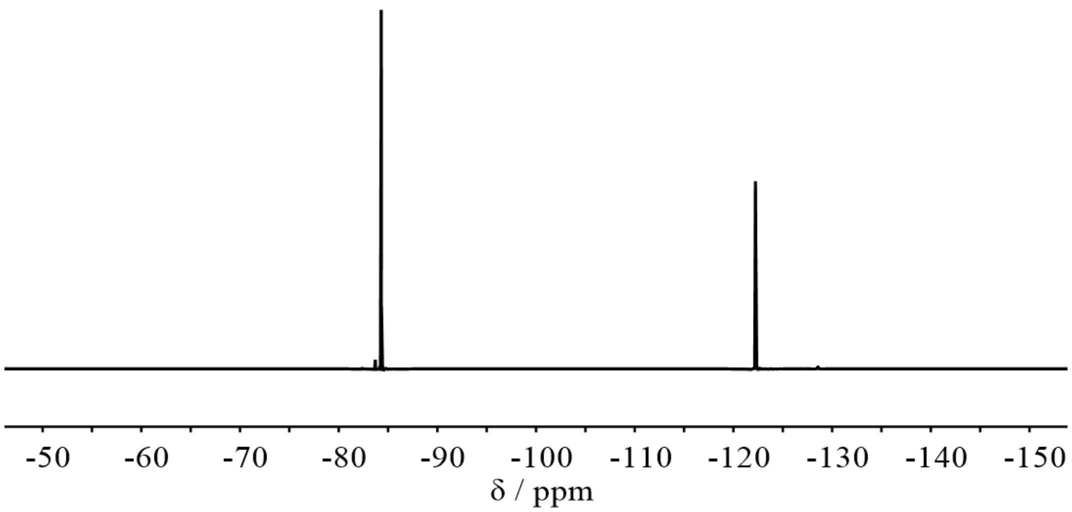

Figure S2. ^{19}F NMR spectrum of $Si(C_2F_5)_3H$, neat with acetone-[d_6] as external standard in a capillary.

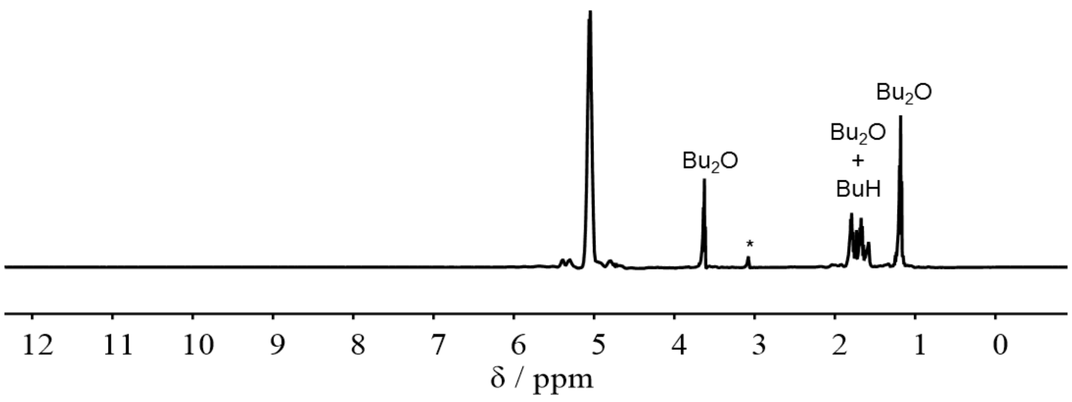

Figure S3. ^{1}H NMR spectrum of $Si(C_2F_5)_3H$, neat with acetone-[d_6] (*) as external standard in a capillary.

2.2 NMR spectra of $[Si(C_2F_5)_3]^-$ salts

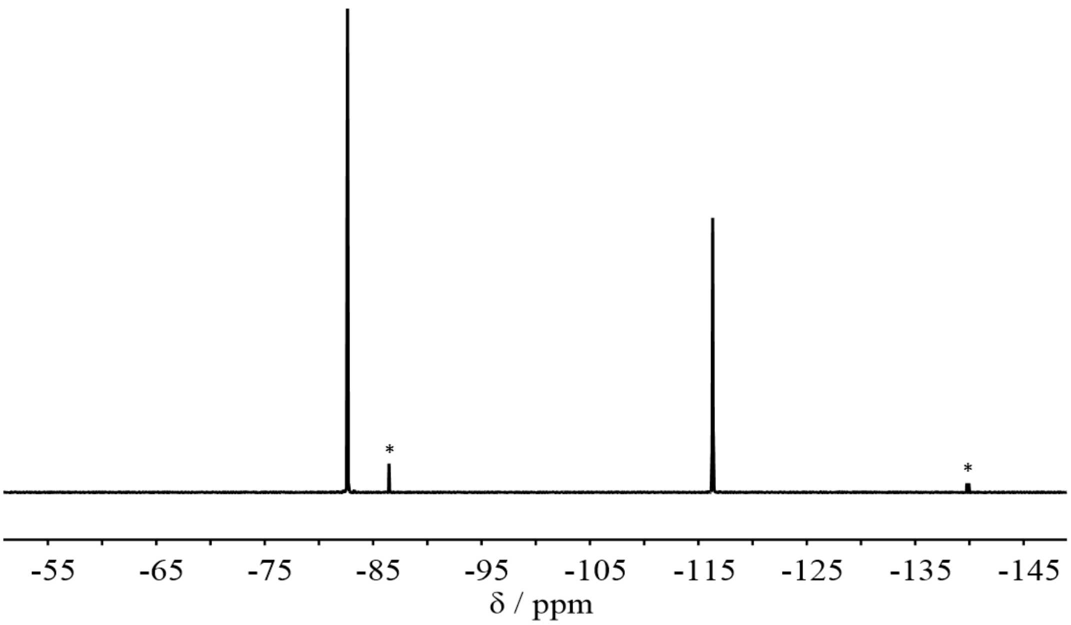

Figure S4. ^{19}F NMR spectrum of $[tmgP_1H][Si(C_2F_5)_3]$ in Et_2O with acetone-$[d_6]$ as external standard in a capillary. * Signals for HC_2F_5.

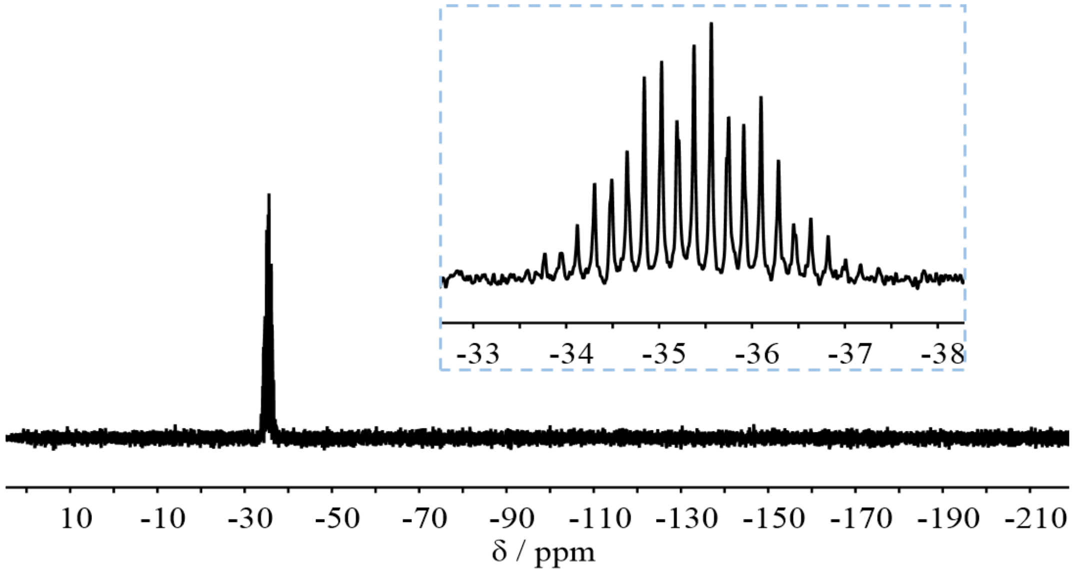

Figure S5. $^{29}Si,^{19}F$-DEPT30 NMR spectrum of $[tmgP_1H][Si(C_2F_5)_3]$ in Et_2O with acetone-$[d_6]$ as external standard in a capillary.

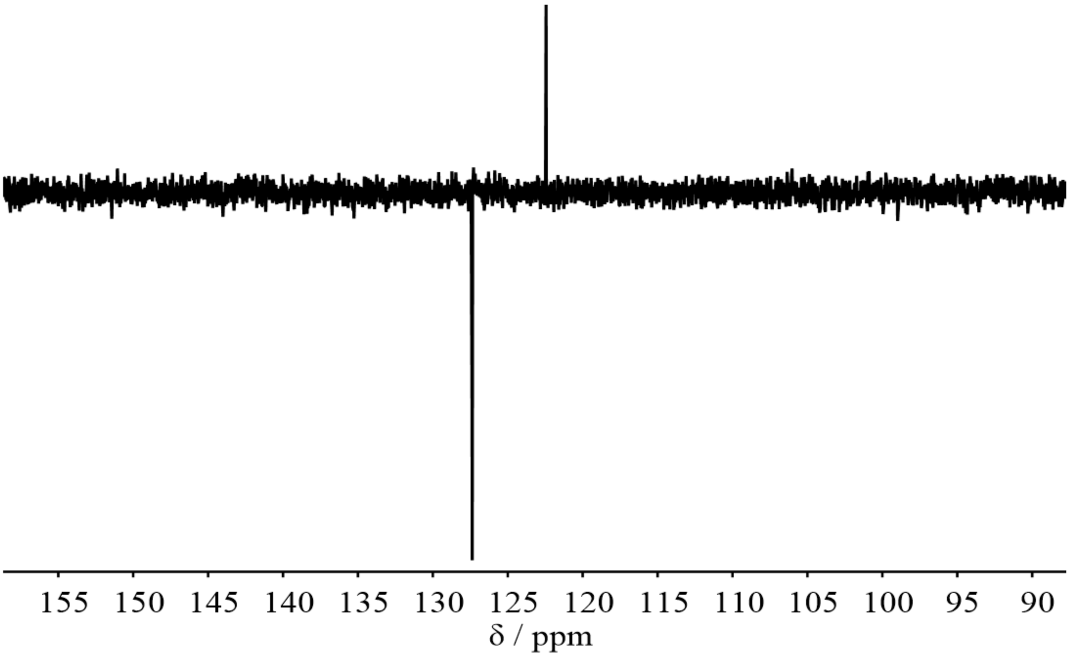

Figure S6. $^{13}C\{^{19}F\}$-DEPT135 NMR spectrum of [tmgP$_1$H][Si(C_2F_5)$_3$] in Et_2O with acetone-[d$_6$] as external standard in a capillary.

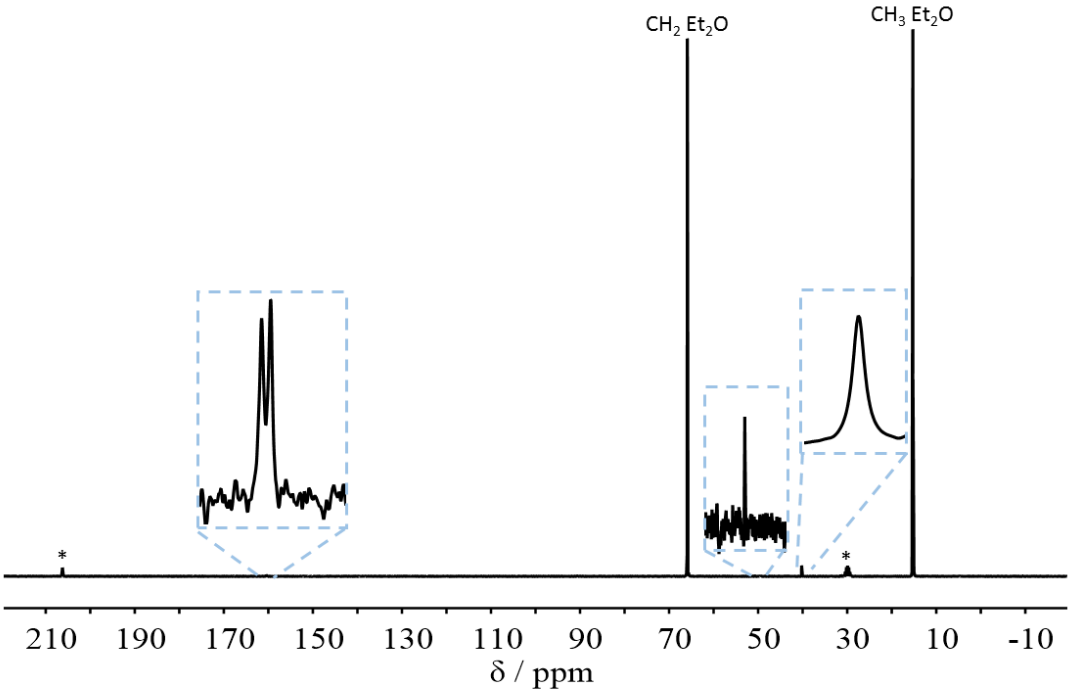

Figure S7. $^{13}C\{^{1}H\}$-IG NMR spectrum of [tmgP$_1$H][Si(C_2F_5)$_3$] in Et_2O with acetone-[d$_6$] (*) as external standard in a capillary.

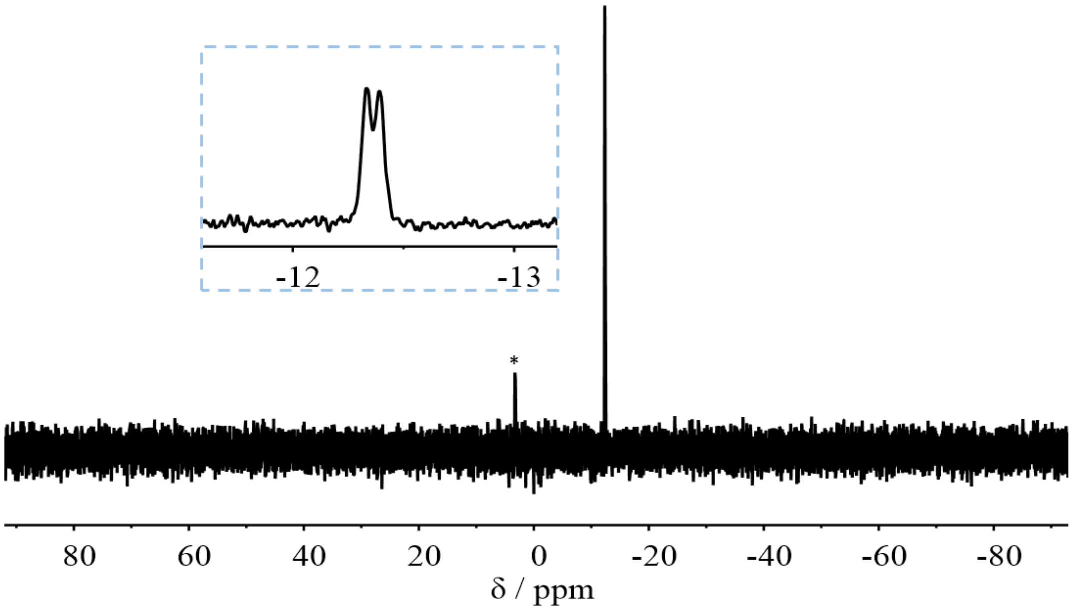

Figure S8. ^{31}P NMR spectrum of [tmgP$_1$H][Si(C$_2$F$_5$)$_3$] in Et$_2$O with acetone-[d$_6$] as external standard in a capillary. * Signals for OP(OMe)$_3$ in the capillary.

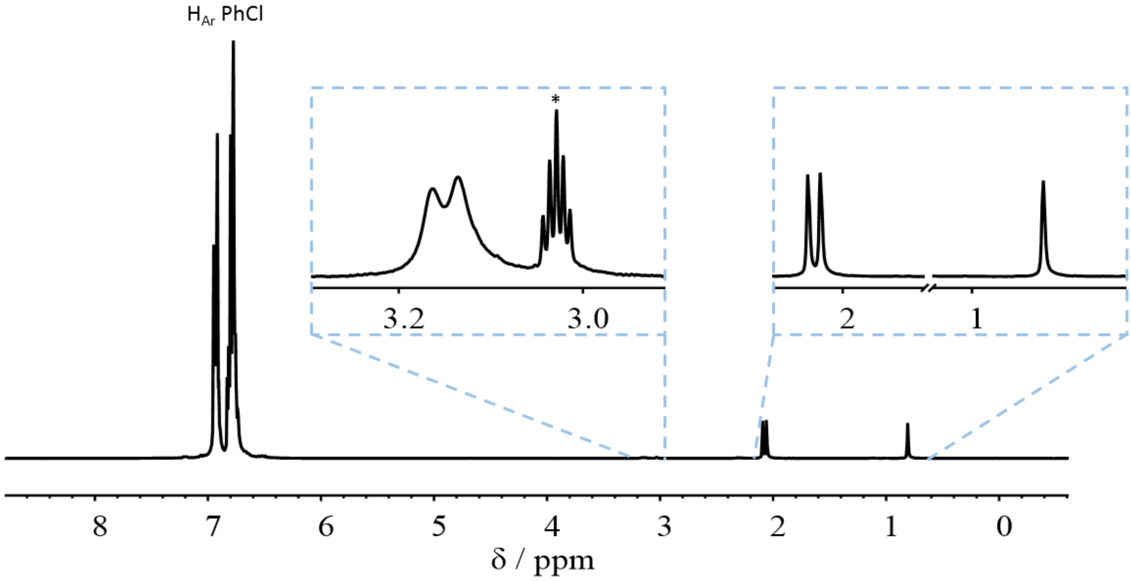

Figure S9. ^{1}H NMR spectrum of [MeP$_1$H][Si(C$_2$F$_5$)$_3$] in PhCl with acetone-[d$_6$] (*) as external standard in a capillary.

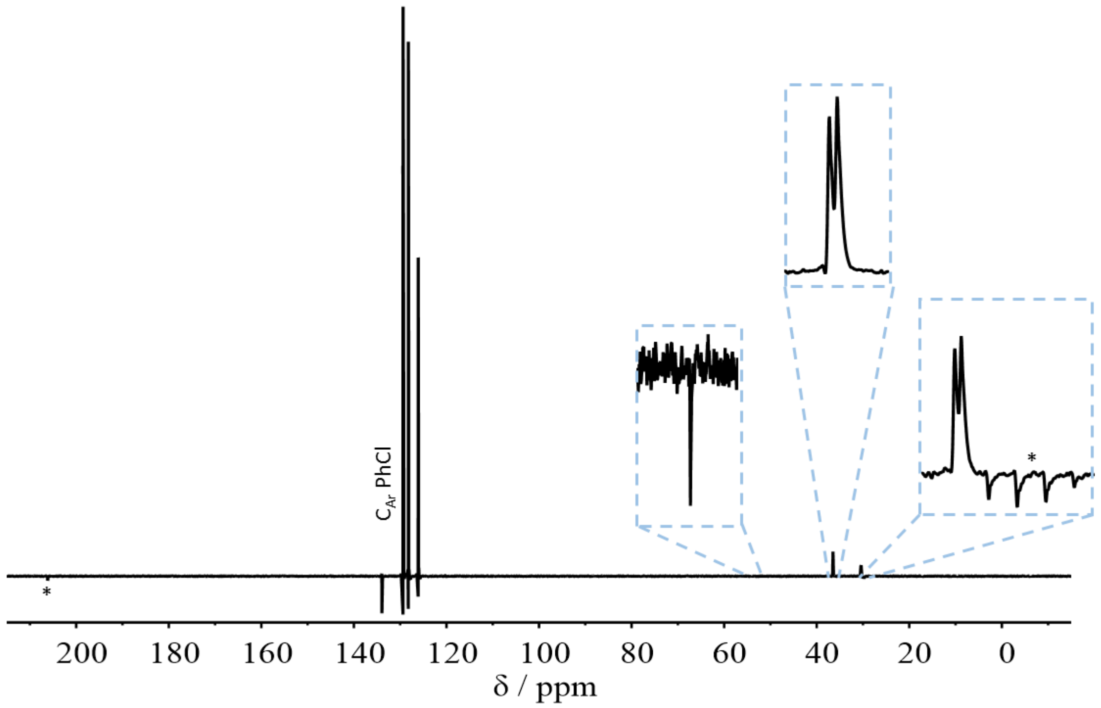

Figure S10. $^{13}C\{^{1}H\}$DEPTq135 NMR spectrum of $[MeP_1H][Si(C_2F_5)_3]$ in PhCl with acetone-$[d_6]$ (*) as external standard in a capillary.

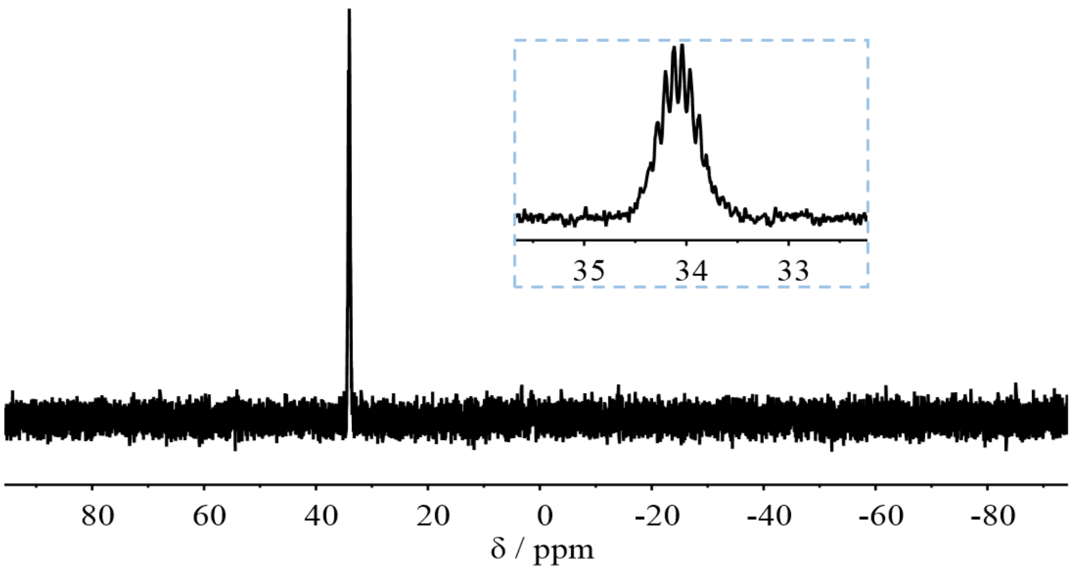

Figure S11. ^{31}P NMR spectrum of $[MeP_1H][Si(C_2F_5)_3]$ in PhCl with acetone-$[d_6]$ as external standard in a capillary.

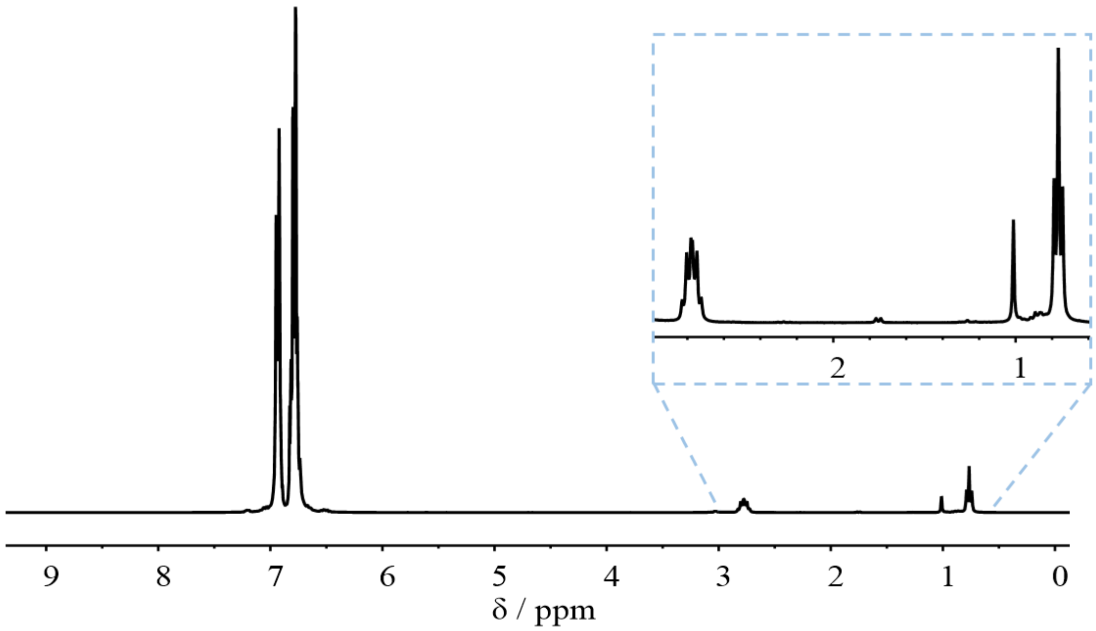

Figure S12. ^{1}H NMR spectrum of $[EtP_4H][Si(C_2F_5)_3]$ in PhCl with acetone-[d_6] as external standard in a capillary.

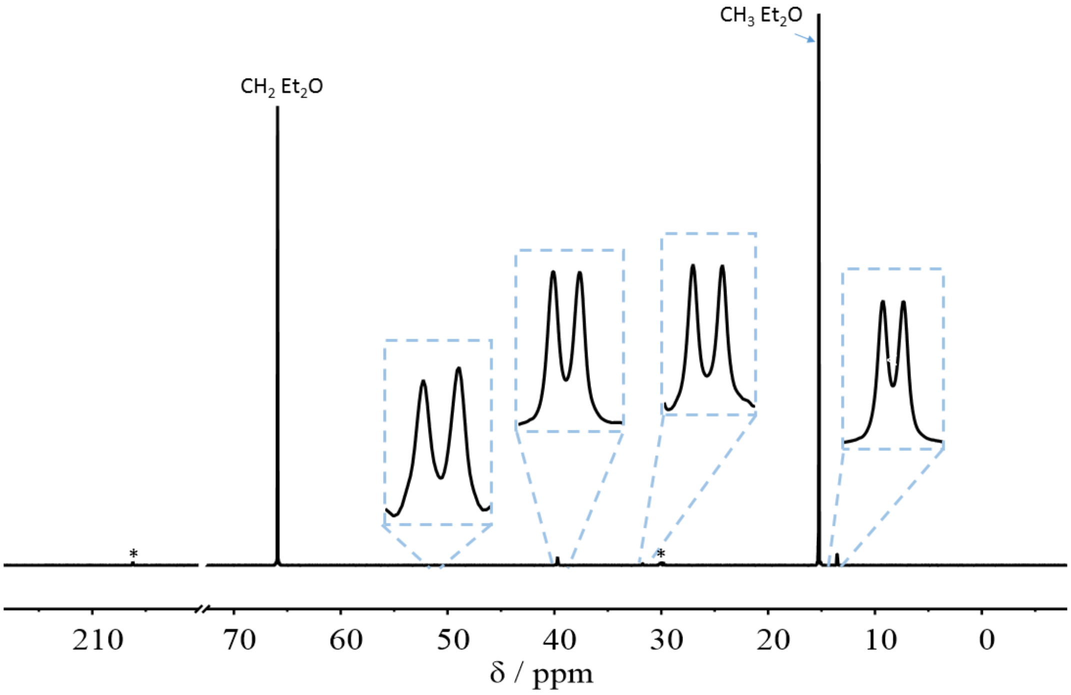

Figure S13. $^{13}C\{^1H\}$IG NMR spectrum of $[EtP_4H][Si(C_2F_5)_3]$ in PhCl with acetone-[d_6] (*) as external standard in a capillary.

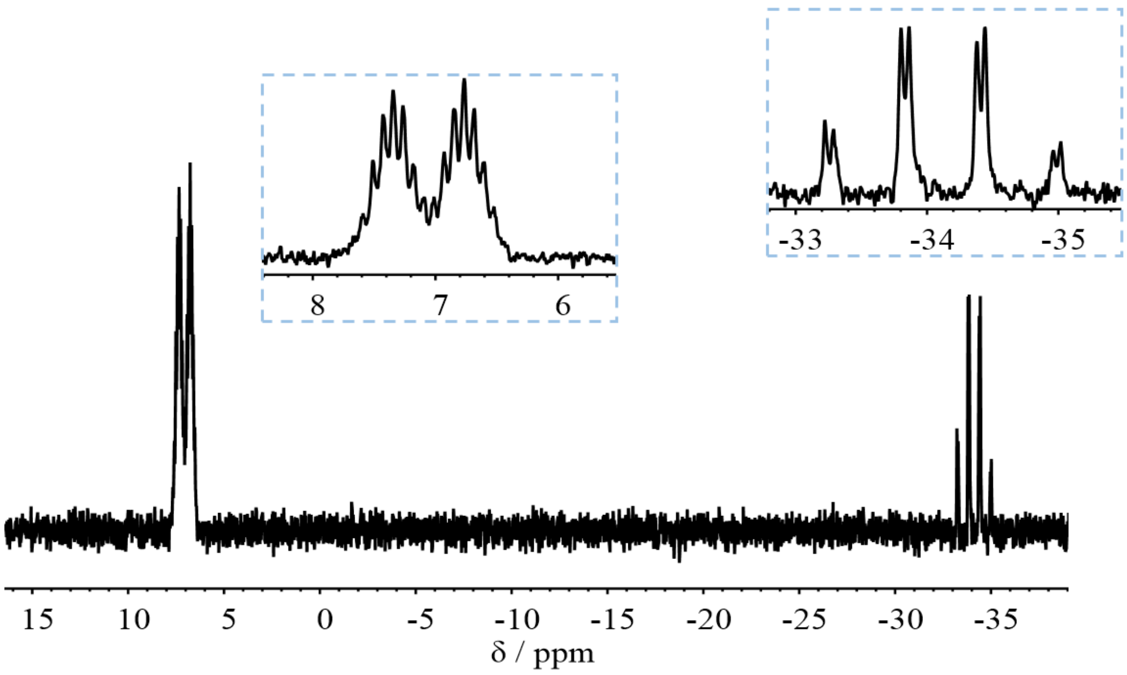

Figure S14. ^{31}P NMR spectrum of $[EtP_4H][Si(C_2F_5)_3]$ in Et_2O with acetone-$[d_6]$ as external standard in a capillary.

2.3 NMR spectra of $[EtP_4H][Si(C_2F_5)_3(\eta^2\text{-}CPhHO)]$

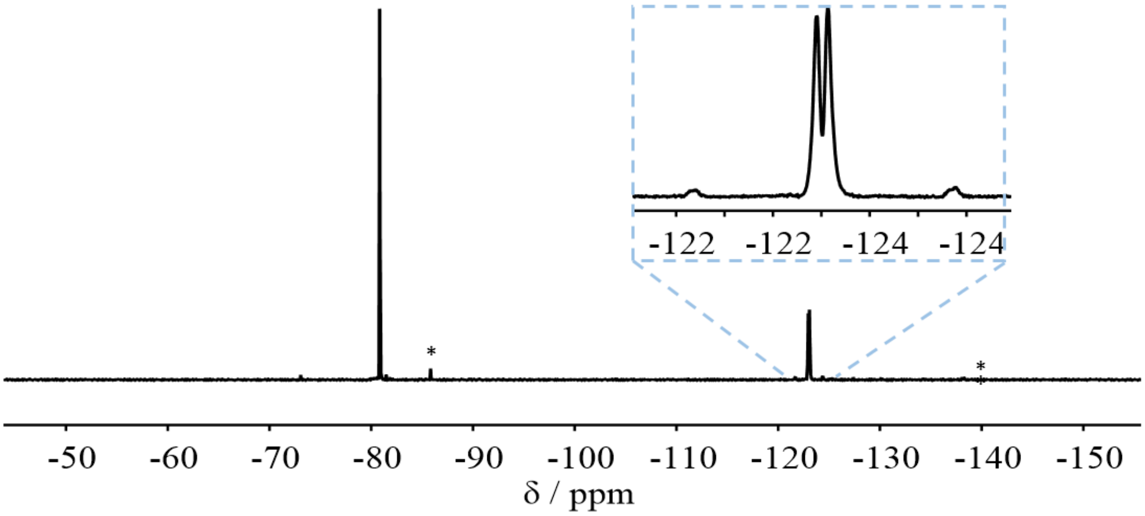

Figure S15. ^{19}F NMR spectrum of $[EtP_4H][Si(C_2F_5)_3(\eta^2\text{-}CPhHO)]$ in PhCl with acetone-$[d_6]$ as external standard in a capillary. *Signals for HC_2F_5.

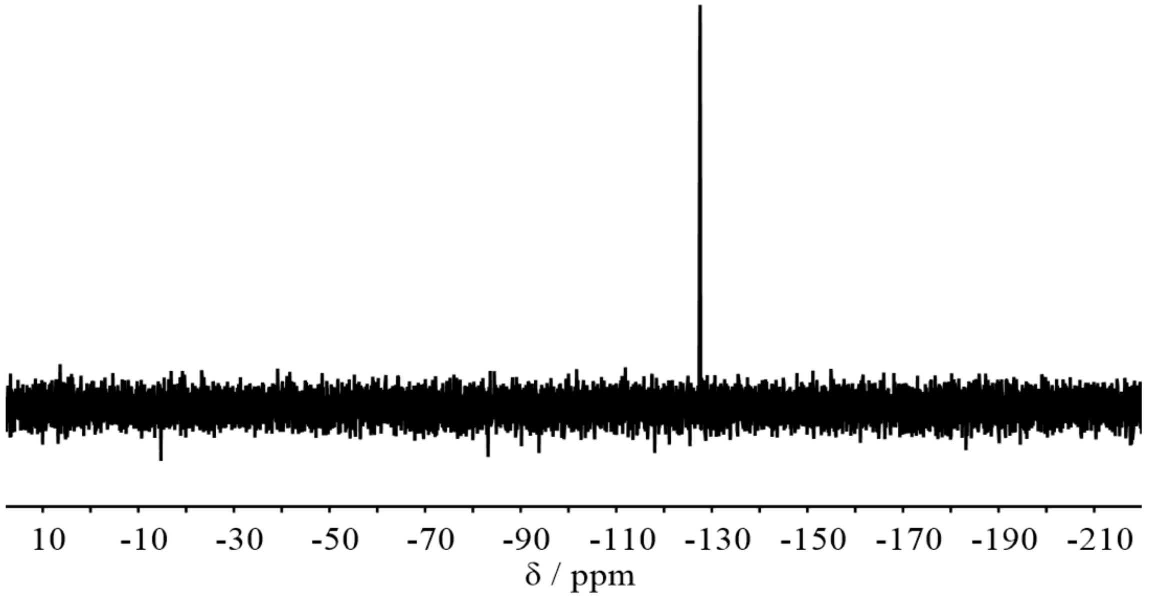

Figure S16. $^{29}Si\{^{19}F\}$ NMR spectrum of $[EtP_4H][Si(C_2F_5)_3(\eta^2\text{-CPhHO})]$ in PhCl with acetone-[d_6] as external standard in a capillary.

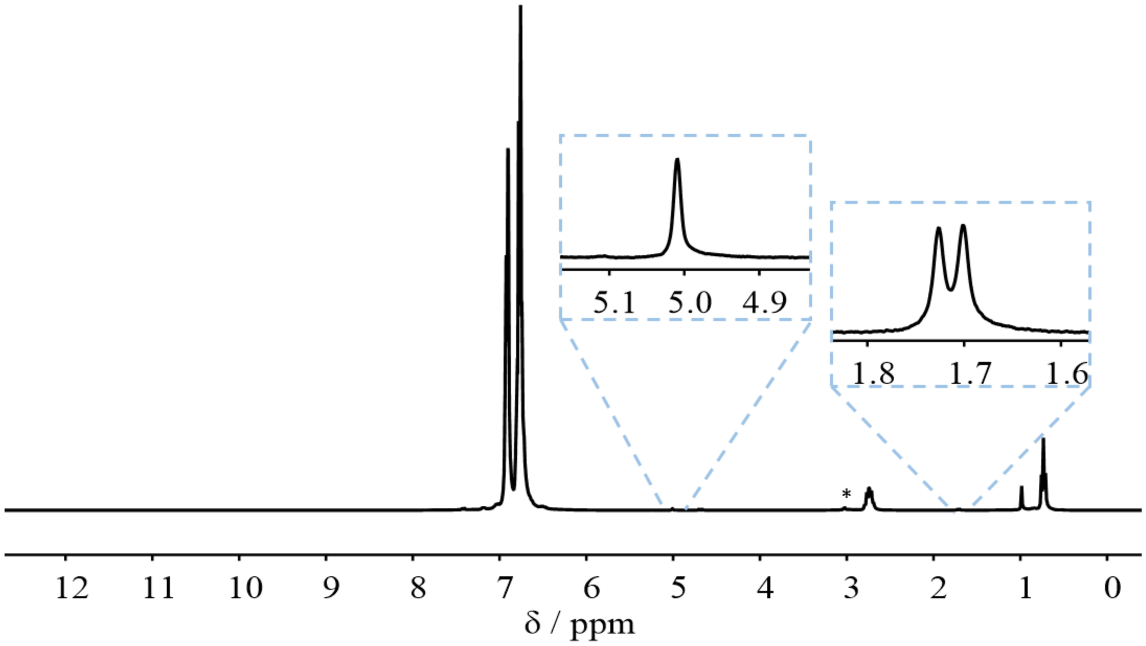

Figure S17. ^{1}H NMR spectrum of $[EtP_4H][Si(C_2F_5)_3(\eta^2\text{-CPhHO})]$ in PhCl with acetone-[d_6] (*) as external standard in a capillary.

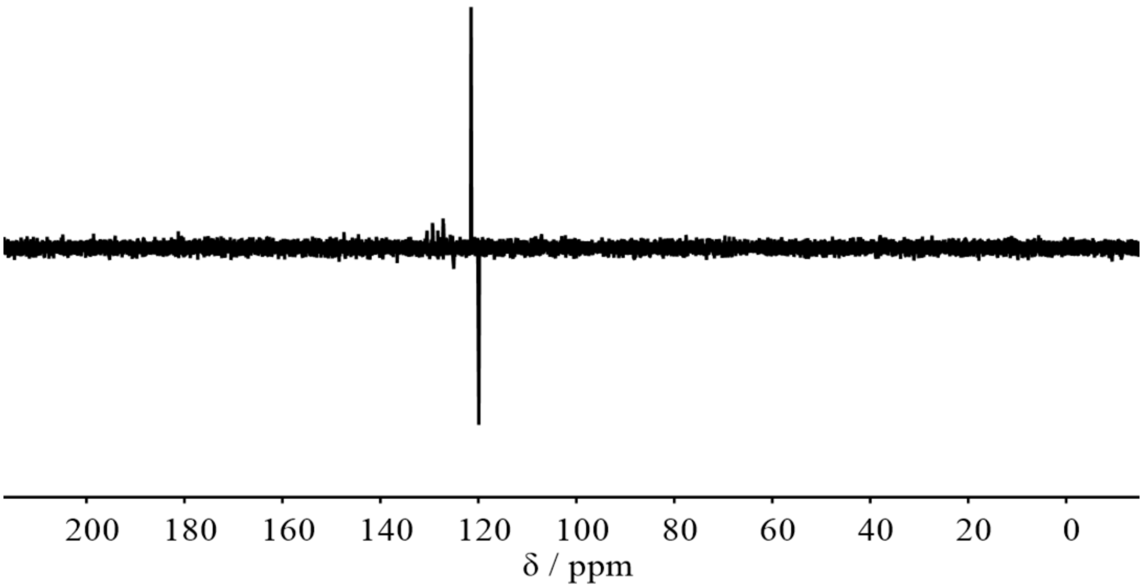

Figure S18. $^{13}C\{^{19}F\}$DEPT135 NMR spectrum of $[EtP_4H][Si(C_2F_5)_3(\eta^2\text{-}CPhHO)]$ in PhCl with acetone-$[d_6]$ as external standard in a capillary.

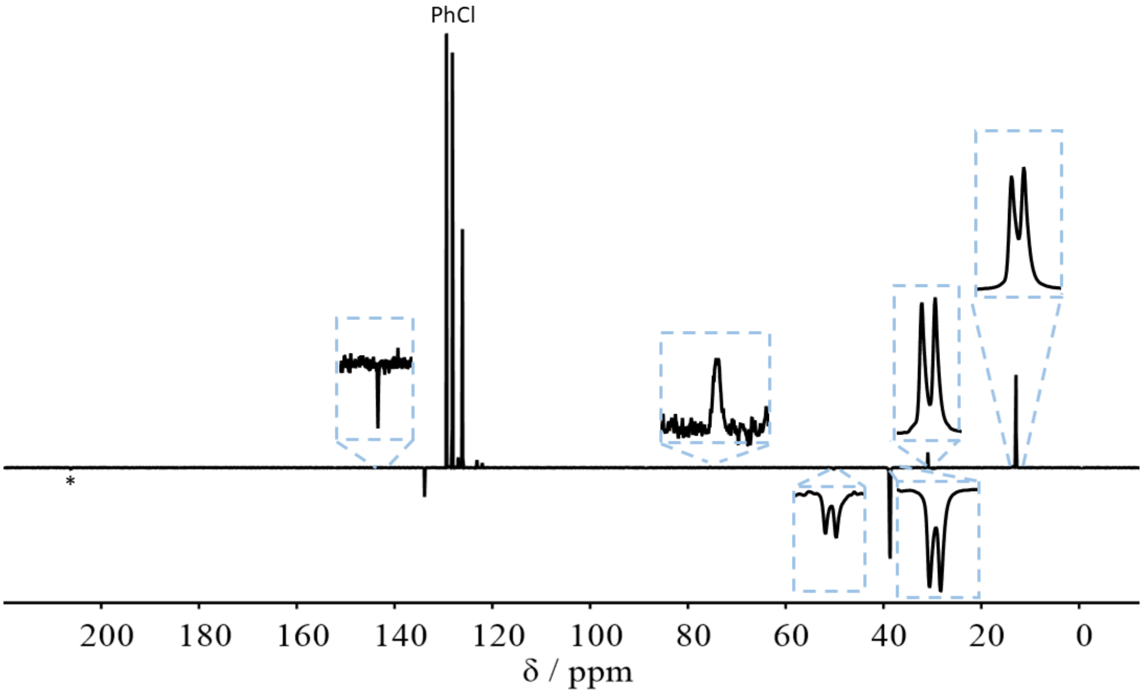

Figure S19. $^{13}C\{^{1}H\}$DEPTq135 NMR spectrum of $[EtP_4H][Si(C_2F_5)_3(\eta^2\text{-}CPhHO)]$ in PhCl with acetone-$[d_6]$ (*) as external standard in a capillary.

2.4 NMR spectra of [EtP$_4$H][Si(C$_2$F$_5$)$_3$(η^2-CPh$_2$O)]

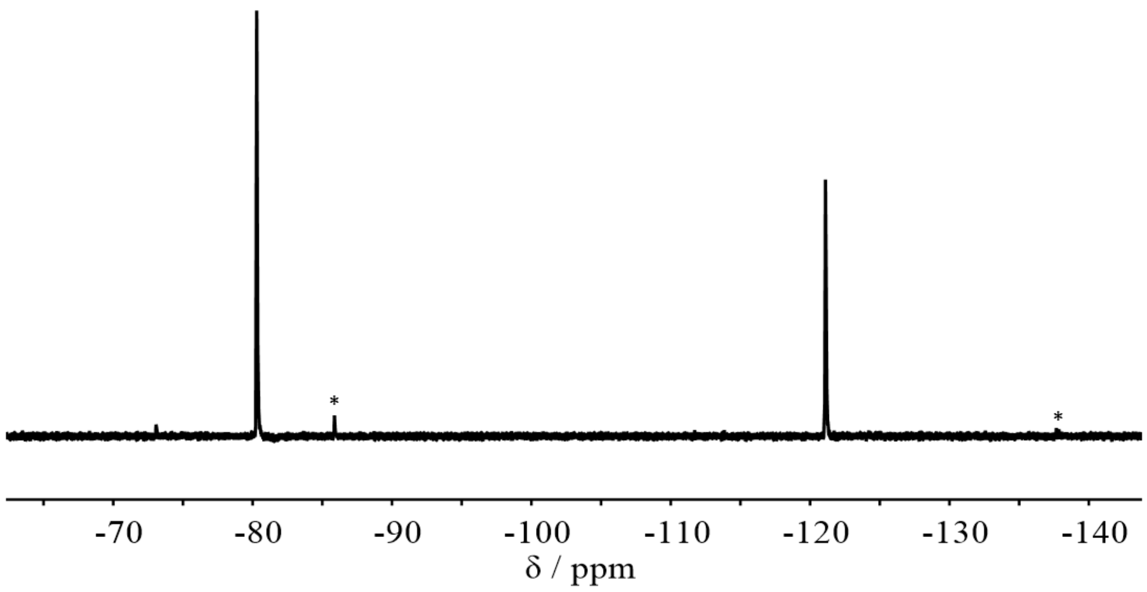

Figure S20. ^{19}F NMR spectrum of [EtP$_4$H][Si(C$_2$F$_5$)$_3$(η^2-CPh$_2$O)] in PhCl with acetone-[d$_6$] as external standard in a capillary.

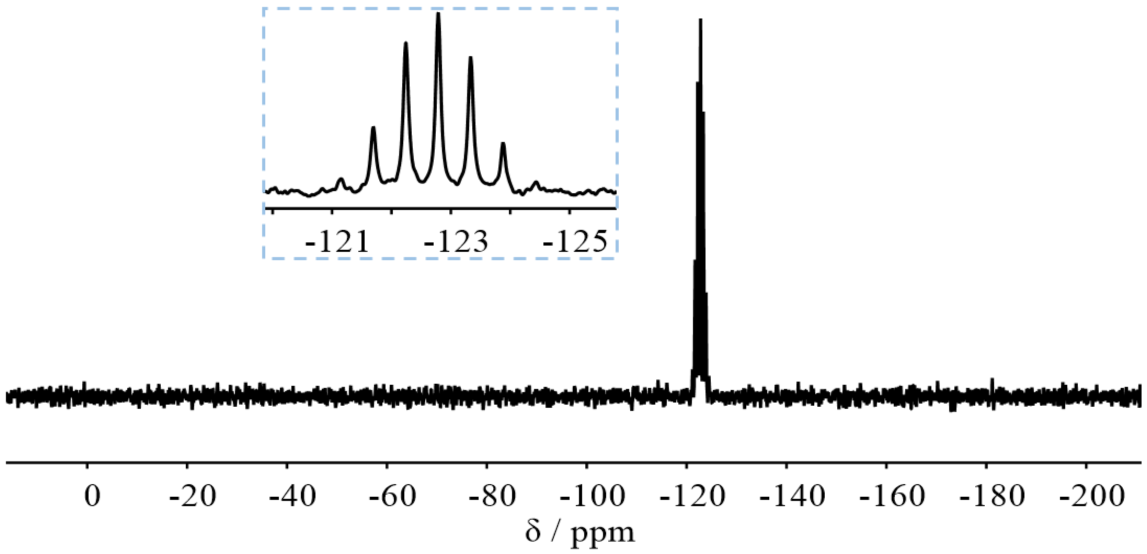

Figure S21. ^{29}Si NMR spectrum of [EtP$_4$H][Si(C$_2$F$_5$)$_3$(η^2-CPh$_2$O)] in PhCl with acetone-[d$_6$] as external standard in a capillary.

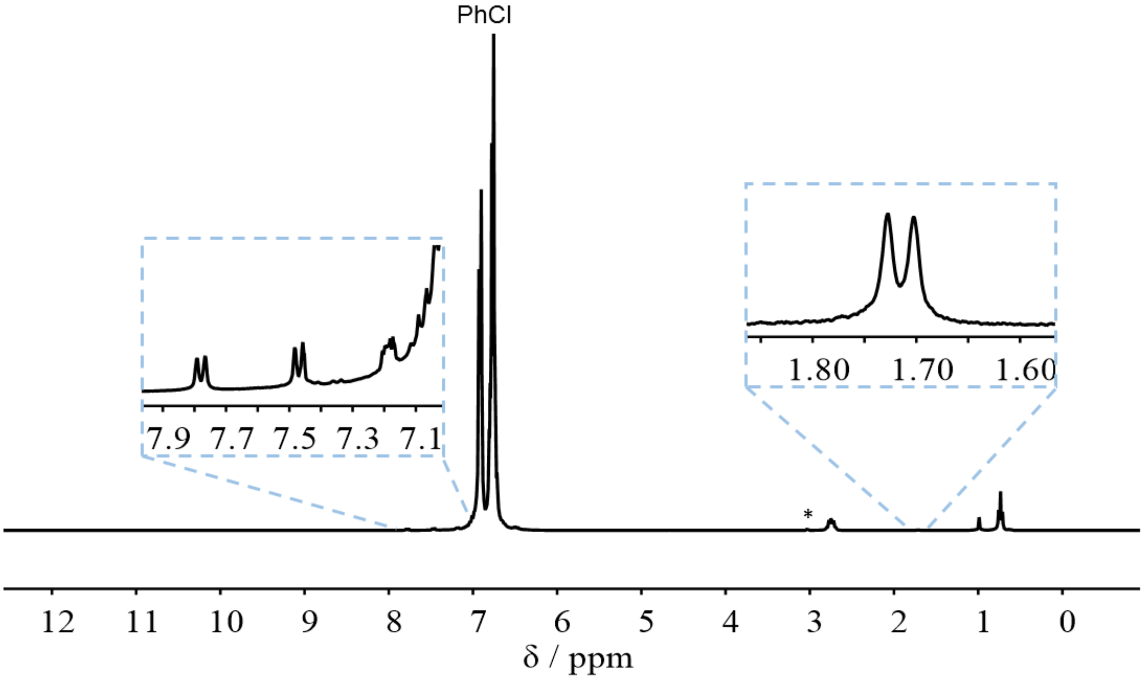

Figure S22. ^{1}H NMR spectrum of [EtP_4H][$Si(C_2F_5)_3(\eta^2$-$CPh_2O)$] in PhCl with acetone-[d_6] (*) as external standard in a capillary.

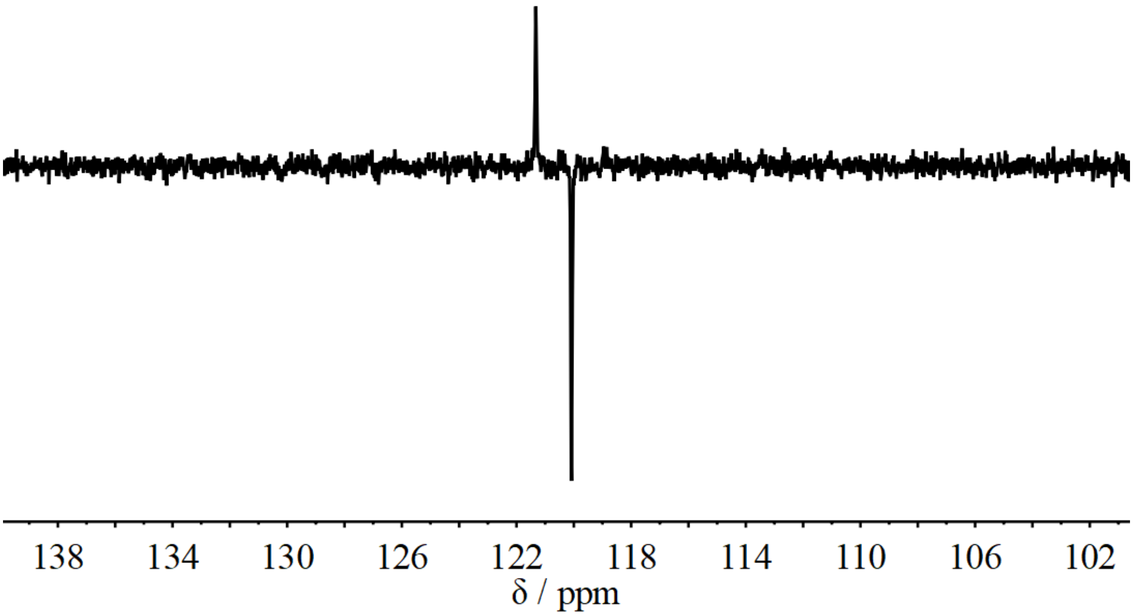

Figure S23. $^{13}C\{^{19}F\}$DEPT135 NMR spectrum of [EtP_4H][$Si(C_2F_5)_3(\eta^2$-$CPh_2O)$] in PhCl with acetone-[d_6] as external standard in a capillary.

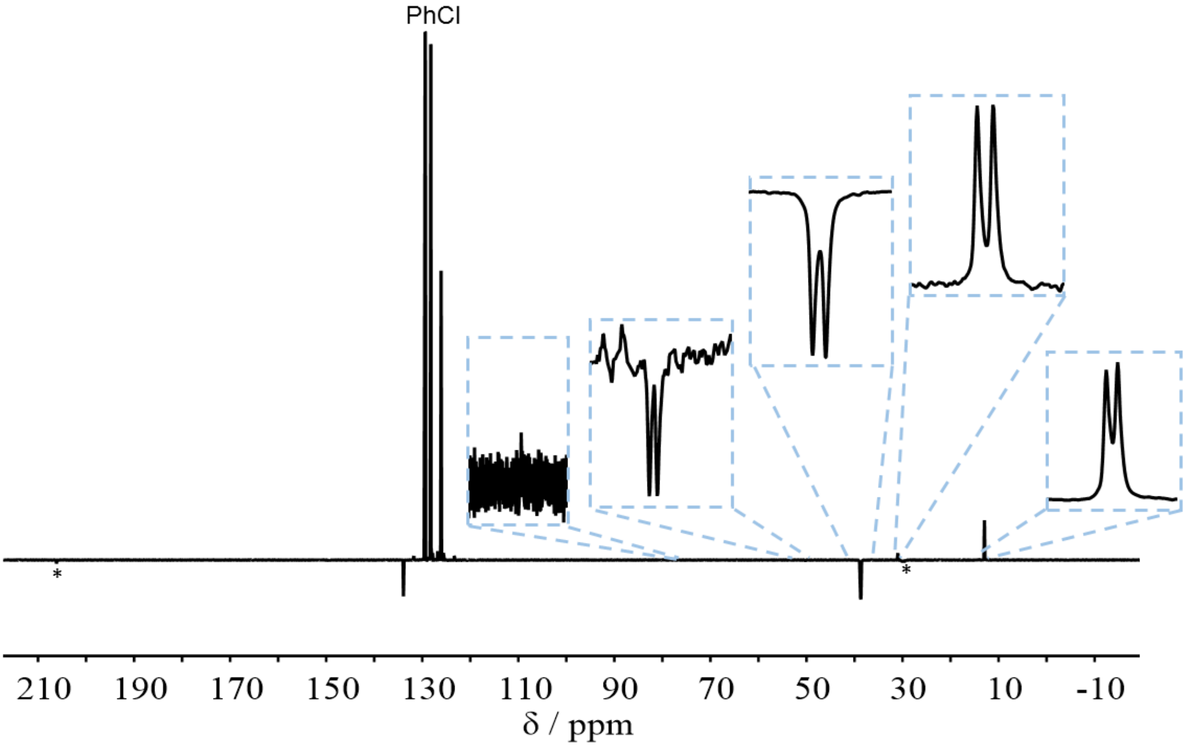

Figure S24. $^{13}C\{^{1}H\}$DEPTq135 NMR spectrum of $[EtP_4H][Si(C_2F_5)_3(\eta^2\text{-}CPh_2O)]$ in PhCl with acetone-[d_6] as external standard in a capillary.

2.5 NMR spectra of $[tmgP_1H][Si(C_2F_5)_3(\eta^2\text{-}CS_2)]$

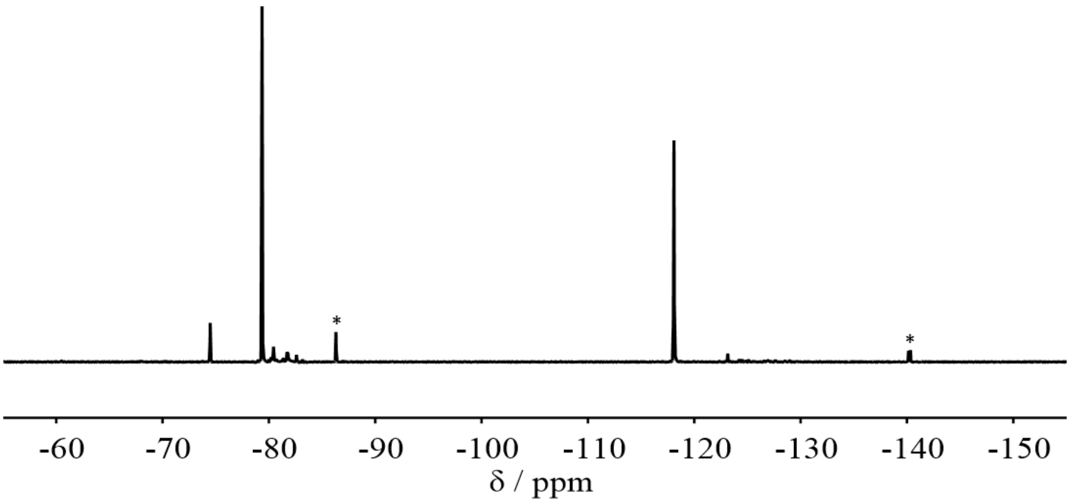

Figure S25. ^{19}F NMR spectrum of $[tmgP_1H][Si(C_2F_5)_3(\eta^2\text{-}CS_2)]$ in Et_2O at 253 K with acetone-[d_6] as external standard in a capillary. * signals for HC_2F_5.

2.6 NMR spectra of [EtP_4H][$Si(C_2F_5)_3(\eta^2-CO_3)$]

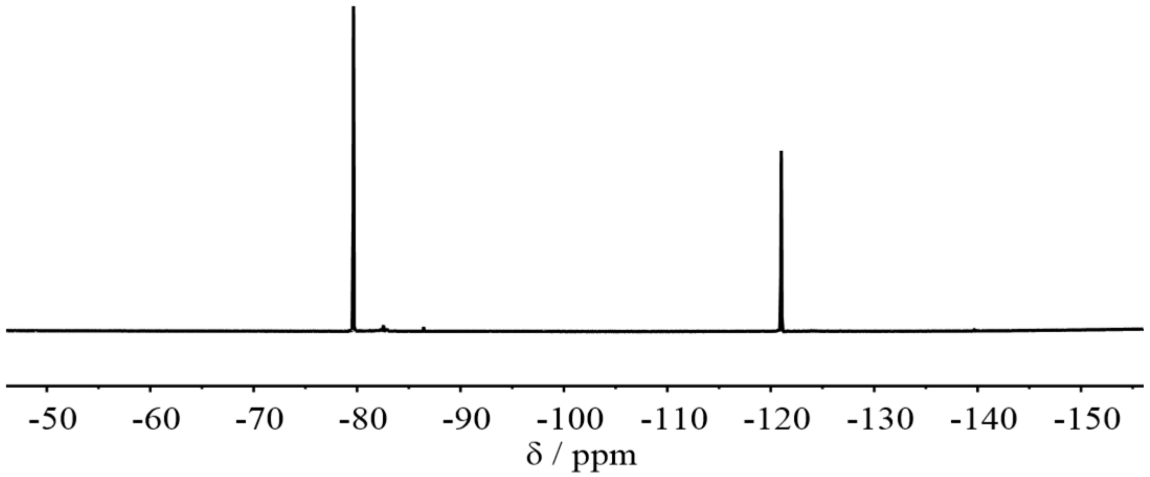

Figure S26. ^{19}F NMR spectrum of [EtP_4H][$Si(C_2F_5)_3(\eta^2-CO_3)$] in CD_3CN.

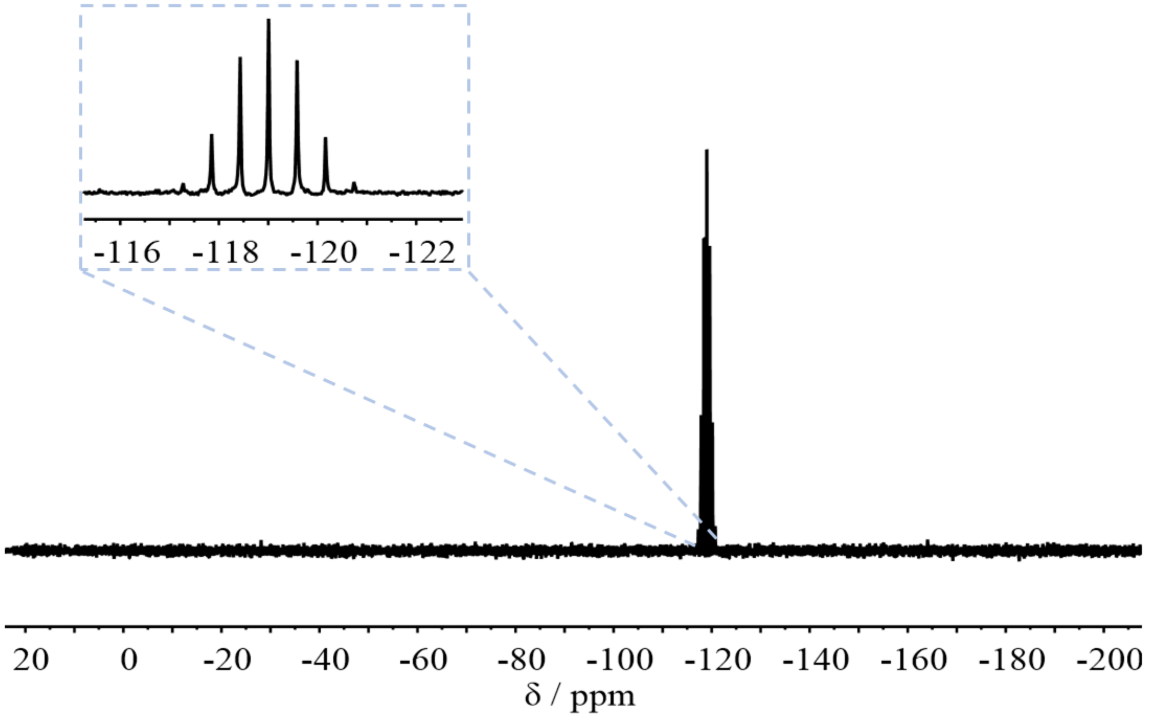

Figure S27. $^{29}Si,^{19}F$-DEPT30 NMR spectrum of [EtP_4H][$Si(C_2F_5)_3(\eta^2-CO_3)$] in CD_3CN.

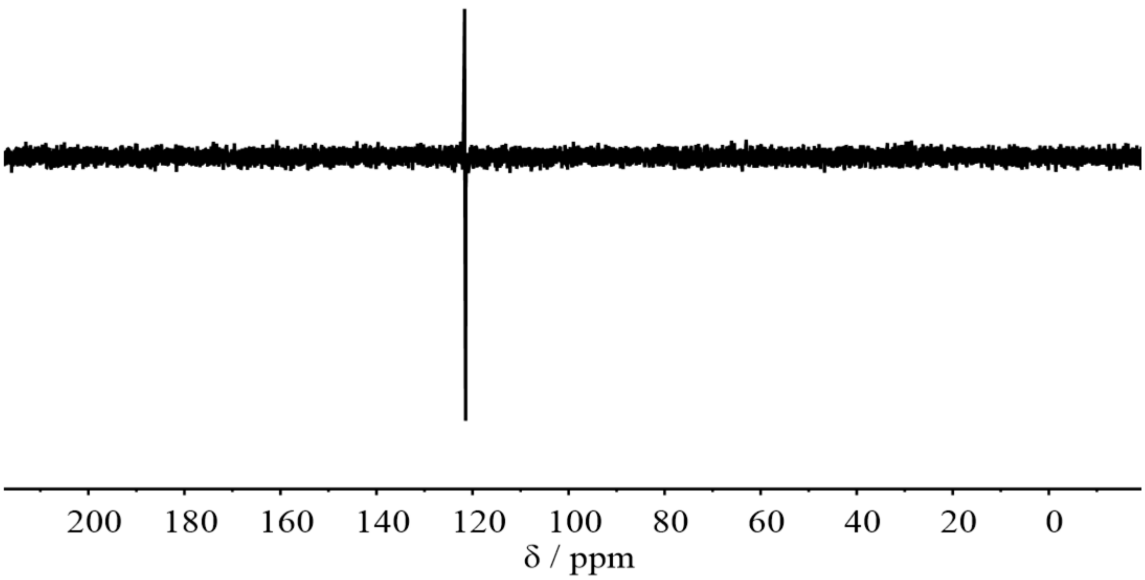

Figure S28. $^{13}C\{^{19}F\}$ NMR spectrum of $[EtP_4H][Si(C_2F_5)_3(\eta^2\text{-}CO_3)]$ in CD_3CN.

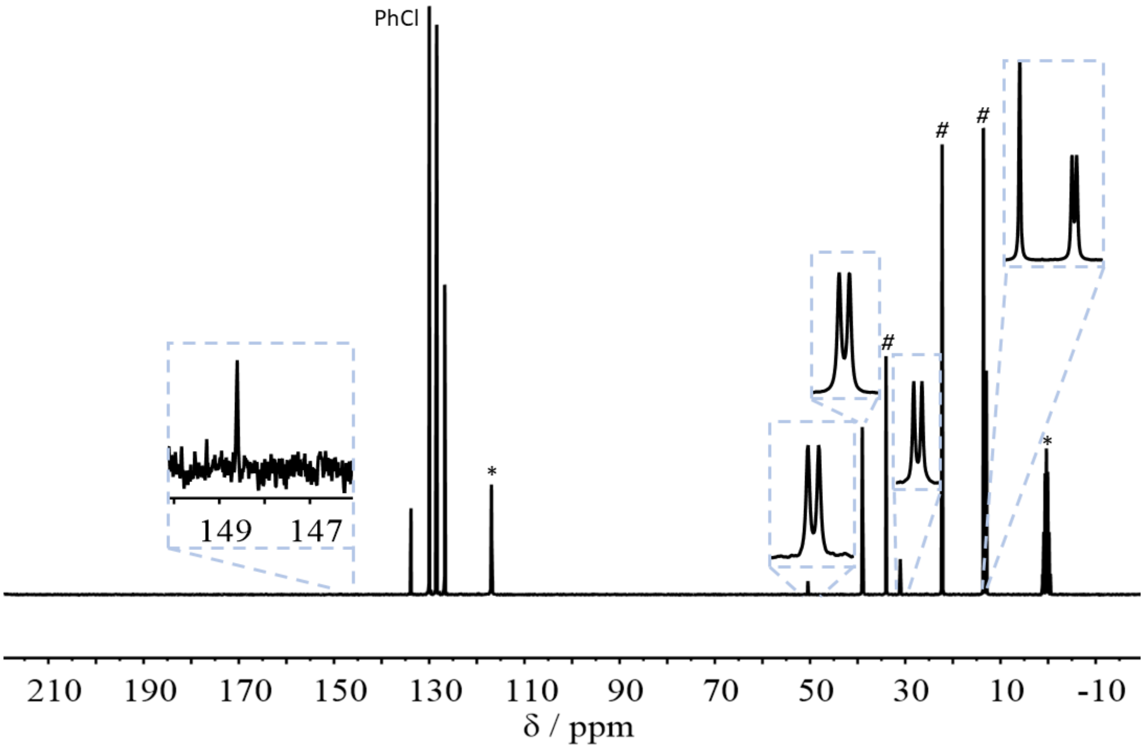

Figure S29. $^{13}C\{^{1}H\}$ NMR spectrum of $[EtP_4H][Si(C_2F_5)_3(\eta^2\text{-}CO_3)]$ in CD_3CN (*) with residues of *n*-pentane (#) and PhCl.

2.7 NMR spectra concerning the catalytic hydrosilylation of benzaldehyde

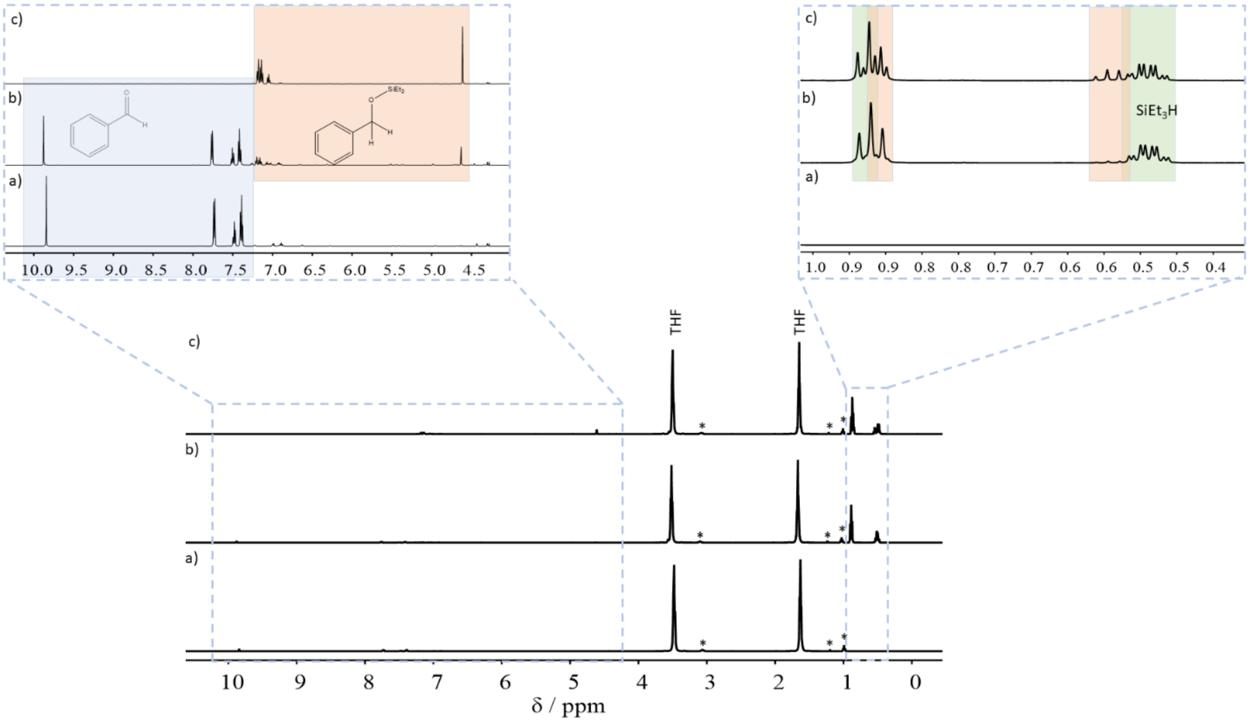

Figure S30. ^{1}H NMR spectrum of the reaction mixture of a) $[EtP_4H][Si(C_2F_5)_3]$ (*) and benzaldehyde (blue); b) $[EtP_4H][Si(C_2F_5)_3]$ (*), benzaldehyde (blue) and triethylsilane (green) after a reaction time of 30 min c) $[EtP_4H][Si(C_2F_5)_3]$ (*), benzaldehyde and triethylsilane (green) after a reaction time of 24 h in THF in which the benzaldehyde is completely converted to (benzyloxy)triethylsilane (red).

2.8 DFT calculations[1,2]

The following calculations were performed using B3LYP/6-31+G(3d,p) with a superfine integration grid (150,974). Frequency calculations were conducted to characterize the stationary points.
To provide reasonable starting structures for optimization towards a transition structure relaxed potential energy scans along the Si-$C_{C=O/C=S}$ bond or the Si-O bond were conducted. Transition structures were located either by a QST2 optimization or by directing the Berny geometry optimization algorithm towards a first order saddle point. To assure that the saddle points found connect the correct minima intrinsic reaction coordinate calculations were performed.

Table S1. Cartesian coordinates of the optimized minimum structures as well as the corresponding electronic energy, the energy with zero point energy correction (ZPE), thermal corrected energy (E^{therm}), Enthalpy (H) and Gibbs free energy (G).

Molecule	Charge	Cartesian coordinates of optimized structure				Electronic Energy /Hartree	Corrected Energies: ZPE, E^{therm}, H, G /Hartree
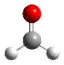	0	C<br>H<br>H<br>O	0.00000000<br>0.00000000<br>0.00000000<br>0.00000000	0.00000000<br>0.94224300<br>-0.94224300<br>0.00000000	-0.52902500<br>-1.11463500<br>-1.11463500<br>0.67542700	-114.518019	-114.491481,<br>-114.488612,<br>-114.487668<br>-114.512490
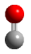	0	C<br>O	0.00000000<br>0.00000000	0.00000000<br>0.00000000	-0.64658100<br>0.48493600	-113.325535	-113.320513,<br>-113.318152,<br>-113.317208,<br>-113.339640
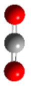	0	C<br>O<br>O	0.00000000<br>0.00000000<br>0.00000000	0.00000000<br>0.00000000<br>0.00000000	0.00000000<br>1.16442100<br>-1.16442100	-188.605103	-188.593401,<br>-188.590790,<br>-188.589846,<br>-188.614096
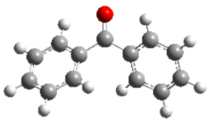	0	C<br>O<br>C<br>C<br>C<br>C<br>H<br>C<br>H<br>C<br>H<br>H<br>H<br>C<br>C<br>C<br>C<br>H<br>C<br>H<br>C<br>H<br>H<br>H	0.00006400<br>0.00016900<br>1.30043900<br>1.44399500<br>2.42691100<br>2.69303100<br>0.58714200<br>3.66727200<br>2.30980500<br>3.80322000<br>2.79865500<br>4.53096300<br>4.77324700<br>-1.30044400<br>-2.42654200<br>-1.44436300<br>-3.66697800<br>-2.30919900<br>-2.69345400<br>-0.58772500<br>-3.80332600<br>-4.53039300<br>-2.79939800<br>-4.77340800	1.09380000<br>2.31676200<br>0.34696200<br>-0.89432200<br>0.95901100<br>-1.51209200<br>-1.36627000<br>0.32999400<br>1.92985300<br>-0.90649700<br>-2.46495200<br>0.80403600<br>-1.39314700<br>0.34709900<br>0.95886200<br>-0.89393900<br>0.32993000<br>1.92944200<br>-1.51161700<br>-1.36575300<br>-0.90620200<br>0.80374700<br>-2.46422500<br>-1.39276600	-0.00027900<br>-0.00047600<br>0.02525700<br>0.66096700<br>-0.54474500<br>0.72446000<br>1.12968700<br>-0.50204500<br>-1.01453000<br>0.13537400<br>1.23431300<br>-0.95856800<br>0.17664300<br>-0.02553400<br>0.54545700<br>-0.66168600<br>0.50301800<br>1.01571400<br>-0.72487100<br>-1.13091500<br>-0.13498900<br>0.96029700<br>-1.23512600<br>-0.17601300	-576.693541	-576.502872,<br>-576.492043,<br>-576.491099,<br>-576.540679
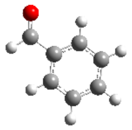	0	C<br>O<br>C<br>C<br>C<br>C<br>H<br>C<br>H<br>C<br>H<br>H<br>H<br>H	1.99252400<br>2.85114900<br>0.53452500<br>-0.35422500<br>0.03841200<br>-1.73040900<br>0.03753400<br>-1.33414300<br>0.74359400<br>-2.21845600<br>-2.41931700<br>-1.72205400<br>-3.29007100<br>2.27175800	0.46472500<br>-0.39278200<br>0.20556900<br>1.28788500<br>-1.10677200<br>1.06558800<br>2.30225500<br>-1.32731900<br>-1.93176100<br>-0.24202300<br>1.90454600<br>-2.34140900<br>-0.41861900<br>1.54132000	-0.00000200<br>0.00000200<br>-0.00000200<br>-0.00000100<br>-0.00000100<br>0.00000100<br>-0.00000100<br>0.00000000<br>-0.00000200<br>0.00000100<br>0.00000100<br>0.00000000<br>0.00000200<br>0.00000500	-345.612898	-345.503505,<br>-345.497158,<br>-345.496214,<br>-345.534107
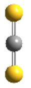	0	C<br>S<br>S	0.00000000<br>0.00000000<br>0.00000000	0.00000000<br>0.00000000<br>0.00000000	0.00000000<br>1.55719800<br>-1.55719800	-834.505333	-834.498440,<br>-834.495327,<br>-834.494383,<br>-834.521378
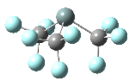	-1	Si<br>C<br>C<br>C<br>F<br>F<br>F<br>F<br>F<br>F<br>F<br>F<br>F	-0.00127900<br>1.30875900<br>-1.62808900<br>0.18736400<br>2.57746900<br>1.39513300<br>1.14586700<br>-2.75900400<br>-1.80429900<br>-1.75191100<br>1.29134400<br>-0.87979800<br>0.30437300	0.02259500<br>-1.21583100<br>-0.79857900<br>1.48724600<br>-0.68162500<br>-2.39121100<br>-1.61444900<br>-0.12020000<br>-2.08172400<br>-0.90262500<br>2.26058300<br>2.35431000<br>1.20086200	-1.09867900<br>-0.24437100<br>-0.28697600<br>0.24255100<br>-0.29172900<br>-0.95765100<br>1.05343100<br>-0.68518400<br>-0.75568600<br>1.07080600<br>-0.04193300<br>0.16154400<br>1.57449600	-1302.551523	-1302.511562,<br>-1302.497970,<br>-1302.497026,<br>-1302.554766

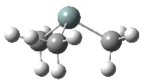	-1	Si	0.00000000	0.00000000	0.67294800	-409.270621	-409.164032, -409.156631, -409.155687, -409.192987
		C	0.00000000	1.70695800	-0.28456900		
		H	0.88270600	2.30942600	-0.02513700		
		H	0.00000000	1.57076800	-1.38273800		
		H	-0.88270600	2.30942600	-0.02513700		
		C	-1.47826900	-0.85347900	-0.28456900		
		H	-1.36032500	-0.78538400	-1.38273800		
		H	-1.55866900	-1.91915900	-0.02513700		
		H	-2.44137400	-0.39026800	-0.02513700		
		C	1.47826900	-0.85347900	-0.28456900		
		H	2.44137400	-0.39026800	-0.02513700		
		H	1.55866900	-1.91915900	-0.02513700		
		H	1.36032500	-0.78538400	-1.38273800		
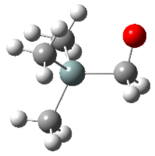	-1	Si	-0.30010000	-0.00000200	0.04379900	-523.835657	-523.699588, -523.689281, -523.688336, -523.734173
		C	-0.38647900	-1.53326400	-1.06801300		
		H	-0.53998100	-2.44584100	-0.47620700		
		H	-1.19282800	-1.47825700	-1.81220800		
		H	0.57228700	-1.62896800	-1.58922700		
		C	-0.38642800	1.53333300	-1.06792000		
		H	-1.19276900	1.47839500	-1.81212800		
		H	-0.53991200	2.44587700	-0.47606000		
		H	0.57234600	1.62904200	-1.58911900		
		C	-1.90953400	-0.00001100	1.09531100		
		H	-1.95925900	-0.88431900	1.74488700		
		H	-1.95923900	0.88426100	1.74493900		
		H	-2.81646000	0.00001800	0.47251500		
		C	1.34781200	-0.00005600	0.97798400		
		H	1.36456900	0.88317500	1.68823000		
		H	1.36456900	-0.88336100	1.68813800		
		O	2.31698200	-0.00000100	0.02236100		
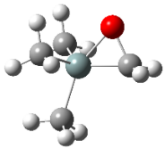	-1	Si	0.07588800	-0.00003300	-0.02066100	-523.834598	-523.697947, -523.688014, -523.687070, -523.731567
		C	0.45837700	-1.59217100	0.97412200		
		H	0.14220400	-2.47477700	0.40031700		
		H	1.52971500	-1.71063400	1.19021600		
		H	-0.09888900	-1.60764400	1.91821800		
		C	0.45953700	1.59272500	0.97273800		
		H	1.53091800	1.71029900	1.18912100		
		H	0.14453900	2.47507800	0.39788900		
		H	-0.09814400	1.60968100	1.91655000		
		C	1.70376400	-0.00088500	-1.15178300		
		H	1.72357800	-0.88504900	-1.80866300		
		H	1.72421300	0.88304800	-1.80897600		
		H	2.64467500	-0.00108300	-0.57484700		
		C	-1.42636100	-0.00047800	-1.10304800		
		H	-1.79335000	0.89457800	-1.63720300		
		H	-1.79421600	-0.89612600	-1.63559300		
		O	-1.73619700	0.00099300	0.32375600		
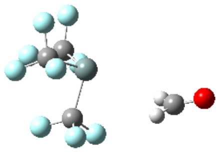	-1	Si	-0.15629600	-0.00265900	-0.55044800	-1417.078825	-1417.011172, -1416.992774, -1416.991829, -1417.064976
		C	-0.54046300	-0.53279500	1.33148100		
		C	0.81154700	1.68071300	-0.11393800		
		C	1.45617800	-1.14472200	-0.78285300		
		F	-0.86967400	-1.86756700	1.39745600		
		F	-1.66677600	0.12733600	1.78504200		
		F	0.38533200	-0.34749100	2.31513900		
		F	1.23833600	2.30391300	-1.26315600		
		F	-0.04247600	2.58133100	0.48248000		
		F	1.91246100	1.65134500	0.69388900		
		F	1.09176900	-2.43593500	-1.08727100		
		F	2.19651700	-0.72029300	-1.86136900		
		F	2.35688200	-1.27064100	0.23678800		
		C	-3.81678300	-0.02486000	-0.85871900		
		H	-3.36758000	0.89372300	-0.43834600		
		H	-3.35940300	-0.97488800	-0.52531300		
		O	-4.74613600	0.00629900	-1.63461200		
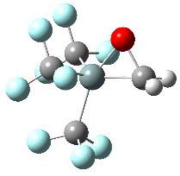	-1	Si	-0.00000100	-0.19371100	0.58423800	-1417.100921	-1417.030134, -1417.014132, -1417.013188, -1417.076057
		C	0.00000400	1.73662200	-0.01078800		
		C	-1.62470900	-0.71956100	-0.39379400		
		C	1.62470600	-0.71956800	-0.39379400		
		F	1.09201600	2.39316000	0.51704900		
		F	-1.09200700	2.39316400	0.51704800		
		F	0.00000600	2.07176400	-1.33578600		
		F	-1.60011100	-2.02642700	-0.78153800		
		F	-2.74125800	-0.58722500	0.39070200		
		F	-1.89786500	-0.01388000	-1.53220800		
		F	2.74125700	-0.58722200	0.39069700		
		F	1.60011000	-2.02643600	-0.78152700		
		F	1.89785700	-0.01389500	-1.53221400		
		C	-0.00000100	-0.20968600	2.40450400		
		H	-0.90295400	-0.05150300	3.00337900		
		H	0.90295400	-0.05150800	3.00337900		
		O	-0.00000400	-1.51736300	1.68839200		

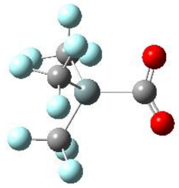	-1	Si	0.08698700	-0.00881400	0.37188800	-1491.159631	-1491.106822, -1491.089600, -1491.088655, -1491.155688
		C	-1.81132900	-0.39369100	0.04943300		
		C	0.98870200	-1.27714700	-0.81768400		
		C	0.31565000	1.69905300	-0.55933900		
		F	-2.61554200	0.32265400	0.87684900		
		F	-2.08735500	-1.70873200	0.28729200		
		F	-2.25787500	-0.14041400	-1.21488300		
		F	2.21676700	-0.84919900	-1.20661900		
		F	1.17151100	-2.48281100	-0.21439800		
		F	0.30017900	-1.54008100	-1.96845600		
		F	-0.60173100	2.61320600	-0.12291700		
		F	1.53243600	2.25626700	-0.35889700		
		F	0.15269900	1.62536000	-1.91303100		
		C	0.81268300	-0.03989300	2.21013900		
		O	2.04775500	0.06388600	2.09330200		
		O	0.03326200	-0.14798600	3.15842400		
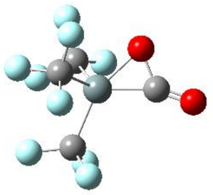	-1	Si	-0.05051400	-0.18576500	0.40942500	-1491.160920	-1491.107724, -1491.091027, -1491.090083, -1491.154480
		C	0.35074500	1.74207900	0.09614200		
		C	-1.64252500	-0.32845800	-0.72530600		
		C	1.45908700	-0.92329300	-0.60500000		
		F	1.34540800	2.17062500	0.93241600		
		F	-0.74042100	2.51621200	0.40282000		
		F	0.73733200	2.13479900	-1.15415500		
		F	-1.76468500	-1.56979500	-1.27400700		
		F	-2.79333400	-0.11146000	-0.02523000		
		F	-1.67637200	0.54247300	-1.77350600		
		F	2.65625500	-0.38674400	-0.21966400		
		F	1.60023500	-2.26563200	-0.49416000		
		F	1.35218700	-0.67007300	-1.94476100		
		C	-0.21774700	-0.62675000	2.19270900		
		O	-0.44341600	-1.69897600	1.41250800		
		O	-0.23653300	-0.52907600	3.39611500		
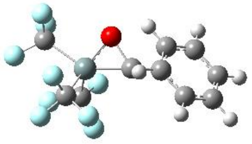	-1	Si	-0.66436700	0.05372600	-0.29250500	-1648.189358	-1648.037817, -1648.017177, -1648.016233, -1648.090563
		C	-0.24855800	-0.48958600	1.55403500		
		C	-2.14211100	-1.25965300	-0.60812500		
		C	-1.80783800	1.65750500	-0.12718000		
		F	0.23942100	0.55320400	2.28971100		
		F	0.71422600	-1.45759900	1.59417700		
		F	-1.29602100	-0.98281900	2.27635700		
		F	-2.57311100	-1.26320800	-1.90588100		
		F	-1.72761100	-2.54728200	-0.36359300		
		F	-3.27442800	-1.09734000	0.14277100		
		F	-1.10960400	2.82852400	-0.06214400		
		F	-2.65541800	1.80206700	-1.19287300		
		F	-2.60307100	1.65012900	0.98267300		
		C	0.97522900	0.73514800	-0.96108500		
		O	0.17724700	-0.32766200	-1.67555900		
		C	2.37056100	0.36183200	-0.65917000		
		C	3.26458500	1.33653200	-0.17315400		
		C	2.85753700	-0.94409800	-0.83657000		
		C	4.58464200	1.01696400	0.13054100		
		H	2.90887200	2.35423500	-0.02873500		
		C	4.18297100	-1.26054600	-0.53672300		
		H	2.18239300	-1.70302900	-1.21651100		
		C	5.05729900	-0.28758100	-0.04943300		
		H	5.25078200	1.78892000	0.50912500		
		H	4.53464400	-2.27914600	-0.68428500		
		H	6.08770600	-0.53848100	0.18750900		
		H	0.92340700	1.67643200	-1.51716600		
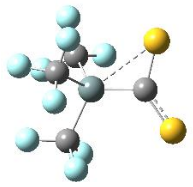	-1	Si	-0.07137100	0.00219400	0.00836200	-2137.079296	-2137.029921, -2137.012296, -2137.011352, -2137.079163
		C	0.89924000	1.69774600	-0.24745900		
		C	0.63845000	-1.10673300	-1.44542300		
		C	0.78513900	-0.66906900	1.63942100		
		F	0.52093800	2.65757700	0.62989500		
		F	0.69058500	2.20188800	-1.49522600		
		F	2.25354800	1.58266400	-0.11022800		
		F	0.76333500	-2.41711300	-1.12289800		
		F	-0.13730500	-1.04646600	-2.55624000		
		F	1.88353500	-0.70059600	-1.83567500		
		F	0.76543900	0.28517700	2.61559200		
		F	0.23994200	-1.77557700	2.17962700		
		F	2.10253500	-0.96010800	1.42258100		
		C	-1.92026600	0.12510800	0.05207600		
		S	-2.76520900	1.56307400	-0.01198900		
		S	-2.43223800	-1.48557700	0.15851300		

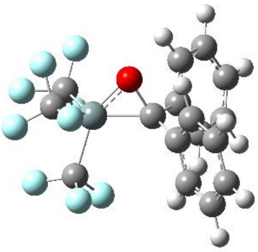	-1	Si	-0.96157100	-0.08575300	-0.18002600	-1879.263692	-1879.031779, -1879.006390, -1879.005445, -1879.091662
		C	-1.38753600	0.46435800	1.70523300		
		C	-1.63741600	-1.92476100	-0.07514500		
		C	-2.42493400	0.95521300	-1.00637400		
		F	-1.41074100	1.82961100	1.84385200		
		F	-0.42146700	0.03382000	2.58007100		
		F	-2.56549300	0.02311600	2.23563200		
		F	-1.34084000	-2.67508200	-1.17023700		
		F	-1.14600300	-2.61582800	0.99494500		
		F	-2.99819000	-1.97497600	0.04480400		
		F	-2.04385600	2.23782000	-1.28889200		
		F	-2.80679800	0.41215500	-2.20173900		
		F	-3.57038400	1.06784400	-0.27465600		
		C	0.90545800	0.05997000	-0.34029200		
		O	0.04663400	-0.04050700	-1.55148800		
		C	1.84434600	-1.10791000	-0.20729800		
		C	2.66850000	-1.28456200	0.91765200		
		C	1.92021200	-2.06406000	-1.23229200		
		C	3.53815200	-2.36909700	1.00874400		
		H	2.62519600	-0.57558700	1.73619400		
		C	2.79691700	-3.14562000	-1.14240100		
		H	1.27485200	-1.94678500	-2.09463200		
		C	3.61243300	-3.30855200	-0.02249100		
		H	4.15783900	-2.48326100	1.89485800		
		H	2.83597800	-3.86905800	-1.95332300		
		H	4.28808500	-4.15646500	0.05154000		
		C	1.57047300	1.41027100	-0.23914200		
		C	1.80324800	2.15764300	-1.40403200		
		C	1.97029000	1.95869400	0.98925100		
		C	2.44422500	3.39402000	-1.34609000		
		H	1.45750200	1.75885700	-2.35143100		
		C	2.61171500	3.19654100	1.04679800		
		H	1.74703000	1.43248300	1.91079700		
		C	2.85866100	3.92004100	-0.12052300		
		H	2.61511200	3.95306000	-2.26298000		
		H	2.90201700	3.60403300	2.01193400		
		H	3.35079400	4.88787800	-0.07431500		
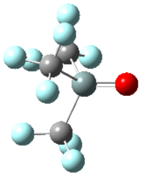	-1	Si	0.00011100	0.00013600	0.76520700	-1377.851440	-1377.806794, -1377.792147, -1377.791203, -1377.850722
		C	-0.40501000	-1.71495600	-0.11145200		
		C	1.68779200	0.50666000	-0.11158000		
		C	-1.28281800	1.20819600	-0.11148700		
		F	-1.45702600	-2.35491100	0.47285800		
		F	0.64709900	-2.58762100	-0.03652500		
		F	-0.71839500	-1.63315700	-1.44103400		
		F	1.91751300	1.85425400	-0.03628100		
		F	2.76800500	-0.08454200	0.47245200		
		F	1.77344500	0.19474600	-1.44120600		
		F	-2.56450300	0.73339900	-0.03570700		
		F	-1.31082500	2.43966300	0.47202600		
		F	-1.05572800	1.43786100	-1.44130400		
		O	0.00030000	0.00018400	2.30333900		
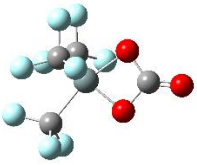	-1	Si	-0.22534700	-0.00010200	-0.14697300	-1566.476976	-1566.417481, -1566.400592, -1566.399648, -1566.464554
		C	1.55019900	0.00063800	-1.05222800		
		C	0.02670800	-1.63392200	0.91733400		
		C	0.02552200	1.63367100	0.91764600		
		F	1.70215100	1.09174900	-1.86668000		
		F	1.70274500	-1.08971000	-1.86756600		
		F	2.64866400	0.00060200	-0.24234200		
		F	-0.51333400	-1.53698200	2.16171100		
		F	-0.59455300	-2.69031700	0.31292000		
		F	1.31703100	-2.02442700	1.10756500		
		F	-0.59724900	2.68948000	0.31374900		
		F	-0.51389900	1.53574000	2.16222000		
		F	1.31544400	2.02567100	1.10744700		
		C	-2.27750400	-0.00060400	-1.08175400		
		O	-2.01921900	-0.00054700	0.22461200		
		O	-3.32982500	-0.00088100	-1.66296400		
		O	-1.02566700	-0.00026500	-1.66784300		
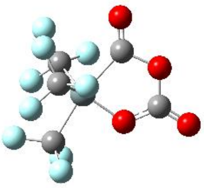	-1	Si	-0.03785000	0.05726400	0.00000700	-1679.785214	-1679.716609, -1679.697848, -1679.696904, -1679.765875
		C	1.55100800	1.28000600	0.00010700		
		C	0.46642400	-0.90118100	-1.66547500		
		C	0.46645100	-0.90149400	1.66530400		
		F	1.58415900	2.10841300	1.08852300		
		F	1.58411800	2.10864400	-1.08813300		
		F	2.75616100	0.63287000	0.00001600		
		F	0.50948700	-2.24952900	-1.50941700		
		F	-0.48668700	-0.65868400	-2.62338500		
		F	1.64752400	-0.55429200	-2.23548100		
		F	-0.48659000	-0.65907800	2.62330600		
		F	0.50940600	-2.24982000	1.50903100		
		F	1.64761000	-0.55478600	2.23529900		
		C	-2.32586100	1.45810600	0.00014100		
		O	-2.83940500	0.19222500	0.00003300		
		O	-3.03908400	2.42321300	0.00022800		
		O	-0.98929300	1.50692100	0.00014000		

C	-1.83879300	-0.83324200	-0.00005800
O	-2.22873900	-1.96342300	-0.00015600

Table S2. Cartesian coordinates of the transition structures as well as the corresponding electronic energy, the energy with zero point energy correction (ZPE), thermal corrected energy (E^{therm}), Enthalpy (H) and Gibbs free energy (G).

Molecule	Charge	Cartesian coordinates of optimized structure				Electronic Energy /Hartree	Corrected Energies: ZPE, E^{therm}, H, G /Hartree
		Si	-0.17875100	0.00000000	0.04982500		
		C	-0.33244600	-1.57175200	-1.00739300		
		H	-0.15487400	-2.46303800	-0.39027400		
		H	-1.33687800	-1.67122400	-1.44430800		
		H	0.41076200	-1.57572700	-1.80946500		
		C	-0.33244700	1.57175200	-1.00739400		
		H	-1.33687800	1.67122200	-1.44431100		
		H	-0.15487800	2.46303800	-0.39027300		
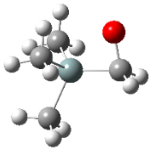	-1	H	0.41076300	1.57572800	-1.80946300	-523.832421	-523.696339, -523.686686, -523.685742, -523.730385
		C	-1.90058200	0.00000000	0.98380400		
		H	-2.00063700	-0.88394800	1.63131500		
		H	-2.00063700	0.88394700	1.63131600		
		H	-2.76384500	0.00000000	0.29865700		
		C	1.31958400	0.00000000	1.11625000		
		H	1.51584200	0.88887900	1.76379700		
		H	1.51584200	-0.88887800	1.76379800		
		O	1.98415900	0.00000000	-0.12624200		
		Si	0.12709200	0.01306900	0.59066600		
		C	-1.81024400	-0.40991100	0.44603800		
		C	0.85225500	-1.29432100	-0.69242800		
		C	0.15444600	1.65872000	-0.48916800		
		F	-2.45705600	-0.18369600	-0.73216200		
		F	-2.51981700	0.29732000	1.38502100		
		F	-2.03608700	-1.72976000	0.74277300		
		F	1.96066400	-0.86570300	-1.34563100		
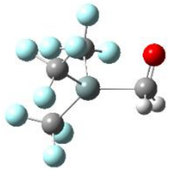	-1	F	1.20416000	-2.45491000	-0.06057500	-1417.065102	-1416.996217, -1416.979950, -1416.979006, -1417.042937
		F	-0.02856400	-1.67621600	-1.67185500		
		F	-0.78462500	2.54535200	-0.01494400		
		F	1.33906600	2.31696800	-0.43155300		
		F	-0.12550600	1.51504200	-1.81925200		
		C	1.60756500	0.12075100	2.20910200		
		H	1.31496400	-0.78642800	2.78405400		
		H	1.23320600	1.02792200	2.73545500		
		O	2.73478700	0.15556600	1.61293700		
		Si	0.14588500	0.04854900	0.37966500		
		C	-1.70082600	-0.66083900	0.31054500		
		C	1.08786500	-1.22135800	-0.77126900		
		C	-0.07532100	1.67386800	-0.68809700		
		F	-2.50262300	-0.01027600	1.20231100		
		F	-1.72540300	-1.97806400	0.67950400		
		F	-2.35299300	-0.59711800	-0.88559000		
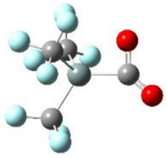	-1	F	2.09426400	-0.66184400	-1.48707800	-1491.158794	-1491.105912, -1491.089666, -1491.088722, -1491.152411
		F	1.63951900	-2.24149200	-0.06020800		
		F	0.26398200	-1.81247700	-1.68972900		
		F	-1.15101200	2.39867900	-0.25144800		
		F	0.97443800	2.51895700	-0.68066600		
		F	-0.32696400	1.39286800	-2.00309200		
		C	1.06511700	0.33794800	2.00692200		
		O	2.08904000	0.65705300	1.30951700		
		O	0.84567400	0.27538800	3.20548900		
		Si	0.37060000	-0.08252600	-0.02805000		
		C	-0.86513200	-1.46843400	-0.70423300		
		C	-0.36502800	1.48722200	-0.97655200		
		C	-0.45109500	0.21854500	1.74447200		
		F	-0.54019400	-2.69988900	-0.20780000		
		F	-0.76333000	-1.58039800	-2.06692900		
		F	-2.19837300	-1.32020200	-0.45147700		
		F	0.01343800	2.65136700	-0.36591600		
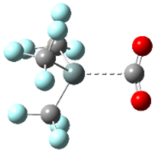	-1	F	0.13061000	1.56009900	-2.25128300	-1491.154365	-1491.102657, -1491.085629, -1491.084685, -1491.153597
		F	-1.72035400	1.57225300	-1.11639500		
		F	-0.45424700	-0.93792600	2.48002100		
		F	0.27847300	1.11601100	2.47618100		
		F	-1.73439300	0.68039700	1.78837500		
		C	2.86260500	-0.41277500	-0.11005100		
		O	3.29958400	0.69876700	-0.09057800		
		O	3.02776800	-1.59469000	-0.14593600		
		Si	0.03634100	0.02187300	-0.06551600		
		C	1.06954000	1.70980100	-0.13788100		
		C	1.11184100	-1.04095700	-1.34484500		
		C	0.77868300	-0.71119600	1.61720400		
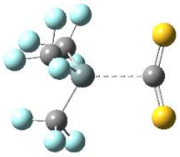	-1	F	0.80173900	2.48776600	0.95781200	-2137.042746	-2136.995820, -2136.977948, -2136.977003, -2137.049466
		F	0.69533200	2.45971800	-1.22144000		
		F	2.43129500	1.63901000	-0.20845000		
		F	0.56423200	-2.28182300	-1.53045600		
		F	1.10809500	-0.45028000	-2.58294000		

		F	2.42691400	-1.27097100	-1.05875600		
		F	0.12909200	-0.18527000	2.70324400		
		F	0.57730300	-2.06426800	1.68994200		
		F	2.10678400	-0.52734200	1.87553400		
		C	-2.71556800	0.04453000	-0.14300700		
		S	-3.11066300	1.58933300	-0.15251600		
		S	-3.11076400	-1.50046800	-0.13823300		
		Si	0.61652000	-0.00828800	0.21324800		
		C	0.58842600	-0.39153800	-1.71552100		
		C	2.24882800	-0.95649200	0.77542700		
		C	1.30411100	1.85247200	0.16652400		
		F	-0.10589400	0.58503100	-2.38177000		
		F	-0.03167100	-1.55962400	-2.01794700		
		F	1.81008000	-0.45098300	-2.32551800		
		F	2.28858400	-1.09734500	2.13488000		
		F	2.36037300	-2.20540800	0.25980100		
		F	3.41447600	-0.31478800	0.44870400		
		F	0.28077700	2.73405500	-0.06130900		
		F	1.81665300	2.19942600	1.39081600		
		F	2.26684600	2.17641500	-0.74079300		
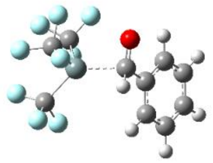	-1	C	-1.02117500	-1.05184900	1.30823400	-1648.150338	-1648.000529, -1647.979764, -1647.978820, -1648.054325
		O	-0.76313700	-2.27979800	1.09625400		
		C	-2.31160000	-0.44533200	0.77272900		
		C	-2.75524100	0.81680500	1.18782300		
		C	-3.11543200	-1.19431300	-0.09158800		
		C	-3.97002900	1.32833300	0.73833800		
		H	-2.13275700	1.40446800	1.86038900		
		C	-4.33211700	-0.68233100	-0.54769500		
		H	-2.76311200	-2.17846600	-0.38405000		
		C	-4.76531200	0.57932100	-0.13665700		
		H	-4.29909700	2.31156300	1.06576500		
		H	-4.94651500	-1.27187000	-1.22424200		
		H	-5.71245600	0.97772300	-0.49119400		
		H	-0.79699700	-0.62048700	2.31036500		
		Si	-0.92929000	-0.09871000	0.00643700		
		C	-1.79089400	0.21760600	1.76801700		
		C	-1.63531900	-1.90169200	-0.36330300		
		C	-2.10978000	0.98438300	-1.14303600		
		F	-1.86585000	1.55524700	2.03861900		
		F	-1.02250100	-0.31573400	2.77545000		
		F	-3.04265800	-0.27495200	1.97767400		
		F	-1.05255200	-2.48417500	-1.44128500		
		F	-1.38623500	-2.73709900	0.69582800		
		F	-2.98116100	-1.98135300	-0.58094300		
		F	-1.66397200	2.26776800	-1.26382700		
		F	-2.21985200	0.49781600	-2.40489500		
		F	-3.39221000	1.09272400	-0.67426800		
		C	1.18450500	0.16043600	-0.86216200		
		O	0.92064000	0.16327300	-2.10626800		
		C	1.95982500	-1.06437400	-0.33836200		
		C	1.90468500	-1.57796600	0.96307700		
		C	2.79813200	-1.69917100	-1.26452800		
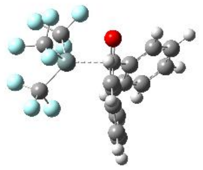	-1	C	2.68329400	-2.67415000	1.33668900	-1879.222587	-1878.992173, -1878.966746, -1878.965801, -1879.051530
		H	1.22248200	-1.14740800	1.68982200		
		C	3.57924000	-2.79399500	-0.89472300		
		H	2.80457200	-1.32237600	-2.28170100		
		C	3.53039600	-3.28535300	0.41083100		
		H	2.61134700	-3.06181700	2.34957000		
		H	4.22013900	-3.27171500	-1.63195100		
		H	4.12922900	-4.14545700	0.69891400		
		C	1.63718900	1.50835600	-0.26205500		
		C	1.54768000	2.62959000	-1.09772100		
		C	2.17888400	1.67958700	1.02022400		
		C	1.96567500	3.88613600	-0.66110500		
		H	1.14330500	2.48155000	-2.09261900		
		C	2.60015200	2.93525600	1.45775300		
		H	2.28043500	0.83339400	1.68898500		
		C	2.49339500	4.04795700	0.62085800		
		H	1.87984900	4.74238700	-1.32582900		
		H	3.01468300	3.04293500	2.45704200		
		H	2.81947900	5.02644600	0.96390100		
		Si	-0.19091300	0.02066700	0.46015200		
		C	-0.58532900	-1.47493600	-0.75790300		
		C	1.77641400	-0.20720000	0.56156000		
		C	-0.21077000	1.62760100	-0.67830900		
		F	-1.59673000	-1.19581400	-1.63146300		
		F	-0.99434000	-2.57670200	-0.05706400		
		F	0.45404200	-1.89701500	-1.53131700		
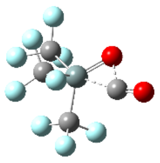	-1	F	2.36507400	0.63707600	1.45842800	-1491.156534	-1491.104911, -1491.088470, -1491.087526, -1491.150806
		F	2.11212700	-1.47145000	0.97652000		
		F	2.46287800	-0.01754300	-0.60946800		
		F	-1.40921600	2.27230500	-0.73086200		
		F	0.68505500	2.56471800	-0.24305400		
		F	0.12363500	1.36656800	-1.97658600		
		C	-2.06045800	0.14365500	1.17917000		

		O	-0.54704200	0.04500300	2.03691000		
		O	-3.03659200	0.20958000	1.81740900		
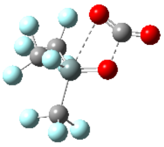	-1	Si	-0.03216800	0.02440900	0.27765700	-1566.452487	-1566.395250, -1566.377878, -1566.376934, -1566.443385
		C	1.74072100	-0.22750000	1.06448500		
		C	0.22745200	1.61662900	-0.82999800		
		C	-0.16503400	-1.46237200	-0.99334900		
		F	1.88990500	-1.48612400	1.56991100		
		F	1.96737000	0.62303200	2.09999500		
		F	2.77754000	-0.04405900	0.19686600		
		F	-0.90698200	2.18811100	-1.28420000		
		F	0.88566400	2.58623600	-0.12416600		
		F	1.00015200	1.36417000	-1.92788400		
		F	-0.80847000	-2.53011200	-0.45847100		
		F	-0.79539000	-1.15862000	-2.15125800		
		F	1.07609000	-1.91773700	-1.35805100		
		C	-2.73609900	0.11587800	0.98453800		
		O	-2.73854500	0.00766800	-0.22417700		
		O	-3.41550200	0.21925500	1.96174800		
		O	-1.06155200	0.12037500	1.47418900		
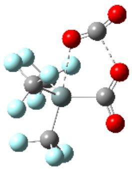	-1	Si	0.19871900	-0.00027300	0.18683900	-1679.759671	-1679.693395, -1679.673924, -1679.672980, -1679.744665
		C	0.09241000	1.62624700	-0.93200900		
		C	0.09276500	-1.62372900	-0.93639800		
		C	2.10955500	-0.00054500	0.64559300		
		F	-0.80328600	2.51003600	-0.41570200		
		F	-0.26910300	1.41532100	-2.21993600		
		F	1.27832000	2.29698200	-0.98361800		
		F	-0.80245200	-2.50925700	-0.42223500		
		F	-0.26936900	-1.40924900	-2.22359100		
		F	1.27886800	-2.29386900	-0.99039400		
		F	2.48281900	1.08690900	1.36445400		
		F	2.48330200	-1.08990200	1.36129800		
		F	2.90659200	0.00123700	-0.47165700		
		C	-3.11124500	-0.00014000	0.16471200		
		O	-2.03753500	-0.00275400	1.91005600		
		O	-4.20710700	-0.00010200	0.58147500		
		O	-2.25016100	0.00023200	-0.65536100		
		C	-0.77601200	-0.00308700	1.89167800		
		O	0.02003800	-0.00519000	2.83823700		
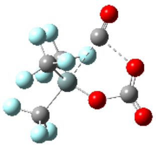	-1	Si	-0.14120800	0.15436100	0.00163800	-1679.763955	-1679.698675, -1679.679023, -1679.678079, -1679.750009
		C	-1.93861200	0.95614900	0.00453600		
		C	-0.26296800	-0.93082300	1.64415400		
		C	-0.25767900	-0.90688200	-1.65688600		
		F	-2.15506400	1.75326800	-1.07695700		
		F	-2.15761500	1.74031800	1.09486600		
		F	-2.95071300	0.03204600	-0.00213300		
		F	0.73113500	-0.61937400	2.51624900		
		F	-1.42594700	-0.73698000	2.32046300		
		F	-0.18111700	-2.27231500	1.42864300		
		F	-1.42176300	-0.70929700	-2.33024800		
		F	0.73483500	-0.57671500	-2.52387600		
		F	-0.16843700	-2.25099500	-1.46203100		
		C	2.18322500	1.91117800	0.01407200		
		O	2.97976900	0.95395300	0.00741700		
		O	2.30497100	3.11585700	0.02096900		
		O	0.72991600	1.49355600	0.01354900		
		C	2.10309400	-1.01957600	-0.00383400		
		O	2.98118600	-1.74598400	-0.00693400		

Section of the NBO analysis of silanide $[Si(CF_3)_3]^-$ (without Rydberg orbitals):

```
        NBO              Occupancy         Energy      Principal Delocalizations (geminal,vicinal,remote)
=================================================================================
Molecular unit  1  (C3F9Si)
 ------ Lewis --------------------------------------
    1. CR ( 1)Si  1          2.00000           -65.04779
    2. CR ( 2)Si  1          1.99825           -5.98749     66(v),63(v),69(v)
    3. CR ( 3)Si  1          1.99967           -3.48607
    4. CR ( 4)Si  1          1.99967           -3.48594
    5. CR ( 5)Si  1          1.99974           -3.48272
    6. CR ( 1) C  2          1.99961           -10.19328   58(g)
    7. CR ( 1) C  3          1.99961           -10.19328   59(g)
    8. CR ( 1) C  4          1.99961           -10.19327   60(g)
    9. CR ( 1) F  5          1.99993           -24.32153   97(v),98(v)
   10. CR ( 1) F  6          1.99993           -24.32070  97(v),98(v)
   11. CR ( 1) F  7          1.99993           -24.31582  96(v),98(v)
   12. CR ( 1) F  8          1.99993           -24.32070  123(v),124(v)
   13. CR ( 1) F  9          1.99993           -24.32153  123(v),124(v)
   14. CR ( 1) F 10          1.99993           -24.31582  122(v),124(v)
   15. CR ( 1) F 11          1.99993           -24.32071  149(v),150(v)
   16. CR ( 1) F 12          1.99993           -24.32153  149(v),150(v)
   17. CR ( 1) F 13          1.99993           -24.31582  148(v),150(v)
   18. LP ( 1)Si  1          1.92982           -0.19538     69(v),63(v),66(v)
```

19. LP (1) F 5	1.98799	-0.96438	97(v),98(v),58(v)
20. LP (2) F 5	1.95865	-0.29951	63(v),62(v),58(v),59(r)
21. LP (3) F 5	1.94415	-0.29695	62(v),63(v),101(v)
22. LP (1) F 6	1.98801	-0.96393	98(v),97(v),58(v),96(v)
23. LP (2) F 6	1.95940	-0.29842	63(v),61(v),58(v),60(r)
24. LP (3) F 6	1.94367	-0.29605	61(v),63(v),101(v)
25. LP (1) F 7	1.98792	-0.95838	96(v),98(v),58(v)
26. LP (2) F 7	1.95693	-0.29435	62(v),61(v),58(v),101(v)
27. LP (3) F 7	1.94039	-0.29369	61(v),62(v),100(v)
28. LP (1) F 8	1.98800	-0.96394	124(v),123(v),59(v),122(v)
29. LP (2) F 8	1.95940	-0.29842	66(v),65(v),59(v),58(r)
30. LP (3) F 8	1.94368	-0.29605	65(v),66(v),127(v)
31. LP (1) F 9	1.98800	-0.96435	123(v),124(v),59(v)
32. LP (2) F 9	1.95865	-0.29951	66(v),64(v),59(v),60(r)
33. LP (3) F 9	1.94414	-0.29696	64(v),66(v),127(v)
34. LP (1) F 10	1.98793	-0.95836	122(v),124(v),59(v)
35. LP (2) F 10	1.95694	-0.29435	64(v),65(v),59(v)
36. LP (3) F 10	1.94038	-0.29368	65(v),64(v),126(v)
37. LP (1) F 11	1.98801	-0.96393	150(v),149(v),60(v),148(v)
38. LP (2) F 11	1.95940	-0.29842	69(v),68(v),60(v),59(r)
39. LP (3) F 11	1.94368	-0.29605	68(v),69(v),153(v)
40. LP (1) F 12	1.98799	-0.96437	149(v),150(v),60(v)
41. LP (2) F 12	1.95864	-0.29952	69(v),67(v),60(v),58(r)
42. LP (3) F 12	1.94415	-0.29697	67(v),69(v),153(v)
43. LP (1) F 13	1.98792	-0.95840	148(v),150(v),60(v)
44. LP (2) F 13	1.95694	-0.29435	67(v),68(v),60(v),153(v)
45. LP (3) F 13	1.94040	-0.29369	68(v),67(v),152(v)
46. BD (1)Si 1- C 2	1.96103	-0.36295	60(g),59(g),64(v),68(v)
			226(v),174(v),200(v),70(g)
47. BD (1)Si 1- C 3	1.96114	-0.36304	58(g),60(g),67(v),61(v)
			304(v),252(v),278(v),71(g)
48. BD (1)Si 1- C 4	1.96093	-0.36293	59(g),58(g),62(v),65(v)
			382(v),356(v),330(v),71(g)
49. BD (1) C 2- F 5	1.99399	-0.85658	63(g),62(g),97(g)
50. BD (1) C 2- F 6	1.99397	-0.85554	63(g),61(g),97(g)
51. BD (1) C 2- F 7	1.99393	-0.86757	62(g),61(g),96(g)
52. BD (1) C 3- F 8	1.99398	-0.85555	66(g),65(g),123(g)
53. BD (1) C 3- F 9	1.99399	-0.85662	66(g),64(g),123(g)
54. BD (1) C 3- F 10	1.99394	-0.86759	64(g),65(g),122(g)
55. BD (1) C 4- F 11	1.99395	-0.85554	69(g),68(g),149(g)
56. BD (1) C 4- F 12	1.99396	-0.85659	69(g),67(g),149(g)
57. BD (1) C 4- F 13	1.99395	-0.86755	67(g),68(g),148(g)
------ non-Lewis ----------------------------------			
58. BD*(1)Si 1- C 2	0.05374	0.29095	
59. BD*(1)Si 1- C 3	0.05374	0.29099	
60. BD*(1)Si 1- C 4	0.05375	0.29099	
61. BD*(1) C 2- F 5	0.09592	0.32308	
62. BD*(1) C 2- F 6	0.09776	0.32339	
63. BD*(1) C 2- F 7	0.10236	0.33366	
64. BD*(1) C 3- F 8	0.09779	0.32323	
65. BD*(1) C 3- F 9	0.09591	0.32319	
66. BD*(1) C 3- F 10	0.10237	0.33370	
67. BD*(1) C 4- F 11	0.09776	0.32335	
68. BD*(1) C 4- F 12	0.09591	0.32307	
69. BD*(1) C 4- F 13	0.10237	0.33365	

Total Lewis 112.75542 (98.9083%)
Valence non-Lewis 1.04937 (0.9205%)
Rydberg non-Lewis 0.19521 (0.1712%)

Total unit 1 114.00000 (100.0000%)
Charge unit 1 -1.00000

Section of the NBO analysis of the transition state $[Si(CF_3)_3(CH_2O)]^-$ (without Rydberg orbitals):

NBO	Occupancy	Energy	Principal Delocalizations (geminal,vicinal,remote)
Molecular unit 1 (C4H2F9OSi)			
------ Lewis --------------------------------------			
1. CR (1)Si 1	2.00000	-65.09681	
2. CR (2)Si 1	1.99806	-6.02374	66(g),69(g),81(v),67(g)
			68(g),74(v),77(v),78(v)
			75(v)
3. CR (3)Si 1	1.99961	-3.52680	
4. CR (4)Si 1	1.99967	-3.52687	

5. CR (5)Si 1	1.99972	-3.52446	
6. CR (1) C 2	1.99960	-10.00148	69(v)
7. CR (1) C 3	1.99948	-10.21564	67(g),74(g),73(g)
8. CR (1) O 4	1.99982	-18.70501	108(v)
9. CR (1) C 7	1.99949	-10.21280	68(g),77(g),78(g)
10. CR (1) C 8	1.99946	-10.21746	69(g),81(g)
11. CR (1) F 9	1.99993	-24.34358	221(v),222(v)
12. CR (1) F 10	1.99993	-24.34418	222(v),221(v),220(v)
13. CR (1) F 11	1.99993	-24.33971	220(v),222(v)
14. CR (1) F 12	1.99993	-24.34250	136(v),135(v),134(v)
15. CR (1) F 13	1.99993	-24.33816	135(v),136(v)
16. CR (1) F 14	1.99993	-24.33438	134(v),136(v)
17. CR (1) F 15	1.99993	-24.33565	194(v),196(v),195(v)
18. CR (1) F 16	1.99993	-24.33613	195(v),196(v)
19. CR (1) F 17	1.99992	-24.33177	194(v),196(v)
20. LP (1) O 4	1.98082	-0.53543	108(v),66(v),71(v),72(v)
			69(r)
21. LP (2) O 4	1.87636	-0.08256	71(v),72(v),110(v),115(v)
			186(r),190(r)
22. LP (3) O 4	1.62235	-0.08460	66(v),109(v),70(g),72(v)
			71(v),69(r),68(r)
23. LP (1) F 9	1.98831	-0.98597	221(v),222(v),69(v)
24. LP (2) F 9	1.95731	-0.32291	81(v),80(v),69(v),68(r)
25. LP (3) F 9	1.94249	-0.31944	80(v),81(v),225(v)
26. LP (1) F 10	1.98827	-0.98581	222(v),221(v),220(v),69(v)
27. LP (2) F 10	1.95615	-0.32388	81(v),79(v),69(v),67(r)
			227(v)
28. LP (3) F 10	1.94248	-0.32027	79(v),81(v),224(v)
29. LP (1) F 11	1.98827	-0.98217	220(v),222(v),69(v),221(v)
30. LP (2) F 11	1.95349	-0.31815	79(v),80(v),69(v)
31. LP (3) F 11	1.93949	-0.31714	80(v),79(v),224(v)
32. LP (1) F 12	1.98860	-0.98643	136(v),135(v),134(v),67(v)
33. LP (2) F 12	1.95714	-0.32069	75(v),74(v),67(v),68(r)
			141(v)
34. LP (3) F 12	1.94402	-0.31713	74(v),75(v),139(v)
35. LP (1) F 13	1.98856	-0.98275	135(v),136(v),67(v)
36. LP (2) F 13	1.95568	-0.31557	75(v),73(v),67(v)
37. LP (3) F 13	1.94075	-0.31422	73(v),75(v),139(v)
38. LP (1) F 14	1.98824	-0.97527	134(v),136(v),67(v)
39. LP (2) F 14	1.95311	-0.31373	74(v),73(v),67(v),69(r)
40. LP (3) F 14	1.93704	-0.31303	73(v),74(v),138(v)
41. LP (1) F 15	1.98851	-0.97767	196(v),194(v),195(v),68(v)
42. LP (2) F 15	1.95621	-0.31430	77(v),78(v),68(v),67(r)
			200(v)
43. LP (3) F 15	1.94094	-0.31057	78(v),77(v),199(v)
44. LP (1) F 16	1.98878	-0.98207	195(v),196(v),68(v)
45. LP (2) F 16	1.95658	-0.31298	78(v),76(v),68(v),198(v)
46. LP (3) F 16	1.94305	-0.31153	76(v),78(v),199(v)
47. LP (1) F 17	1.98833	-0.97319	194(v),196(v),68(v),195(v)
48. LP (2) F 17	1.95274	-0.31119	76(v),77(v),68(v),69(r)
			201(v)
49. LP (3) F 17	1.93726	-0.30894	76(v),77(v),198(v)
50. BD (1)Si 1- C 2	1.90991	-0.25190	69(g),67(g),68(g),74(v)
			81(v),161(v),77(v),66(g)
51. BD (1)Si 1- C 3	1.95683	-0.41510	69(g),68(g),66(g),376(v)
			80(v),350(v),76(v),324(v)
			83(g)
52. BD (1)Si 1- C 7	1.95738	-0.41167	69(g),67(g),66(g),79(v)
			428(v),454(v),402(v),73(v)
			82(g)
53. BD (1)Si 1- C 8	1.95002	-0.41150	66(g),68(g),67(g),75(v)
			298(v),272(v),246(v),78(v)
			82(g)
54. BD (1) C 2- O 4	1.99428	-0.81776	69(v),108(g)
55. BD (1) C 2- H 5	1.98341	-0.38368	160(v),69(v),68(v)
56. BD (1) C 2- H 6	1.98383	-0.38339	160(v),67(v),69(v)
57. BD (1) C 3- F 12	1.99360	-0.87620	74(g),75(g)
58. BD (1) C 3- F 13	1.99375	-0.88732	73(g),75(g),135(g)
59. BD (1) C 3- F 14	1.99403	-0.89940	74(g),73(g),134(g)
60. BD (1) C 7- F 15	1.99392	-0.88144	77(g),78(g)
61. BD (1) C 7- F 16	1.99365	-0.87710	76(g),78(g),195(g)
62. BD (1) C 7- F 17	1.99405	-0.89636	77(g),76(g),194(g)
63. BD (1) C 8- F 9	1.99343	-0.88362	81(g),80(g),221(g)
64. BD (1) C 8- F 10	1.99347	-0.88713	81(g),79(g)
65. BD (1) C 8- F 11	1.99357	-0.89831	79(g),80(g),220(g)

```
------ non-Lewis ----------------------------------
  66. BD*( 1)Si  1- C  2       0.32626     0.16708
  67. BD*( 1)Si  1- C  3       0.08115     0.26792
  68. BD*( 1)Si  1- C  7       0.08244     0.26988
  69. BD*( 1)Si  1- C  8       0.14590     0.27175
  70. BD*( 1) C  2- O  4       0.00340     0.64398
  71. BD*( 1) C  2- H  5       0.06300     0.53004
  72. BD*( 1) C  2- H  6       0.06282     0.53113
  73. BD*( 1) C  3- F 12       0.09825     0.30262
  74. BD*( 1) C  3- F 13       0.10318     0.31227
  75. BD*( 1) C  3- F 14       0.09026     0.32602
  76. BD*( 1) C  7- F 15       0.09680     0.31629
  77. BD*( 1) C  7- F 16       0.09889     0.31159
  78. BD*( 1) C  7- F 17       0.08950     0.32910
  79. BD*( 1) C  8- F  9       0.09641     0.30363
  80. BD*( 1) C  8- F 10       0.09447     0.30506
  81. BD*( 1) C  8- F 11       0.10527     0.31320
  -------------------------------
  Total Lewis  128.11471  ( 98.5498%)
  Valence non-Lewis    1.63799  (  1.2600%)
  Rydberg non-Lewis    0.24729  (  0.1902%)
  -------------------------------
  Total unit  1  130.00000  (100.0000%)
  Charge unit  1   -1.00000
```

2.9 X-ray Data

All crystals were examined on a Rigaku Supernova diffractometer using CuKα (λ = 1.54184 Å) radiation. Using Olex2,[3] the structures were solved with the ShelXT[4] structure solution program using Intrinsic Phasing if not otherwise stated, and refined with the ShelXL[5] refinement package using Least Squares minimization. CCDC 2005658-2005660 and 2027760 contain the supplementary crystallographic data for this paper. These data can be obtained free of charge from The Cambridge Crystallographic Data Centre via www.ccdc.cam.ac.uk/conts/retrieving.html.

Table S3. Structure refinement data of [EtP_4H][$Si(C_2F_5)_3$], [$tmgP_1H$][$Si(C_2F_5)_3(\eta^2$-$CPh_2O)$], [$tmgP_1H$][$Si(C_2F_5)_3(\eta^2$-$CS_2)$] and [EtP_4H][$Si(C_2F_5)_3(\eta^2$-$CO_3)$].

	**[EtP_4H][$Si(C_2F_5)_3$][a]**	**[$tmgP_1H$] [$Si(C_2F_5)_3(\eta^2$-$CPh_2O)$][b]**	**[$tmgP_1H$] [$Si(C_2F_5)_3(\eta^2$-$CS_2)$][c]**	**[EtP_4H][$Si(C_2F_5)_3(\eta^2$-$CO_3)$][d]**
Empirical formula	$C_{46}H_{100}F_{15}N_{13}P_4Si$	$C_{38}H_{56}F_{15}N_{10}OPSi$	$C_{26}H_{46}F_{15}N_{10}PS_2Si$	$C_{47}H_{100}F_{15}N_{13}O_3P_4Si$
Formula weight	1272.35	1012.98	906.91	1332.36
Temperature/K	100.0(1)	100.0(1)	100.0(1)	100.0(1)
Crystal system	triclinic	triclinic	monoclinic	monoclinic
Space group	$P\bar{1}$	$P\bar{1}$	$P2_1/n$	$P2_1/c$
a/Å	13.06406(10)	15.2688(4)	8.58401(6)	16.1785(3)
b/Å	21.8714(2)	15.4367(4)	21.13256(12)	15.2455(3)
c/Å	22.5673(2)	21.5634(5)	22.29838(15)	26.5606(4)
α/°	93.2499(8)	93.182(2)	90	90
β/°	92.9427(7)	100.391(2)	95.0427(6)	90.2858(16)
γ/°	91.2473(7)	107.490(2)	90	90
Volume/Å^3	6427.30(10)	4735.3(2)	4029.32(5)	6551.1(2)
Z	4	4	4	4
ρ_{calc} g/cm^3	1.315	1.421	1.495	1.351
μ/mm^{-1}	2.017	1.673	2.815	2.040
F(000)	2704.0	2104.0	1872.0	2824.0
Crystal size/mm^3	0.36 x 0.32 x 0.20	0.13 x 0.10 x 0.03	0.42 x 0.32 x 0.25	0.42 x 0.19 x 0.09
2Θ range for data collection/°	5.476 to 144.7	6.044 to 144.258	5.772 to 153.196	5.462 to 153.108
Index ranges	−16 ≤ h ≤ 16, −27 ≤ k ≤ 27, −27 ≤ l ≤ 26	−18 ≤ h ≤ 18, −19 ≤ k ≤ 19, −26 ≤ l ≤ 26	−10 ≤ h ≤ 10, −26 ≤ k ≤ 26, −27 ≤ l ≤ 28	−20 ≤ h ≤ 20, −16 ≤ k ≤ 19, −33 ≤ l ≤ 33
Reflections collected	231625	85459	107219	49496
Independent reflections	25367	18650	8402	13512
R(int)	0.0594	0.0871	0.0576	0.0576
R(sigma)	0.0245	0.0596	0.0217	0.0420
Reflections with I > 2σ(I)	23340	13813	7823	10611
Data/restraints/ parameters	25367/0/1542	18650/1/1271	8402/15/541	13512/0/773

	$[EtP_4H][Si(C_2F_5)_3]$[a]	$[tmgP_1H][Si(C_2F_5)_3(\eta^2\text{-}CPh_2O]$[b]	$[tmgP_1H][Si(C_2F_5)_3(\eta^2\text{-}CS_2)]$[c]	$[EtP_4H][Si(C_2F_3)_3(\eta^2\text{-}CO_3)]$[d]
Goodness-of-fit on F^2	1.047	1.102	1.074	1.026
Final R indexes [I > 2σ(I)]	R_1 = 0.0694<br>wR_2 = 0.1901	R_1 = 0.0751<br>wR_2 =0.2072	R_1 = 0.0465<br>wR_2 = 0.1088	R_1 = 0.0559<br>wR_2 = 0.1524
Final R indexes [all data]	R_1 = 0.0731<br>wR_2 = 0.1940	R_1 = 0.0974<br>wR_2 =0.2265	R_1 = 0.0496<br>wR_2 = 0.1105	R_1 = 0.0716<br>wR_2 = 0.1683
Largest diff. peak/hole / e Å^{-3}	1.14/−1.03	0.90/−1.04	0.80/−0.45	0.93/−0.50
CCDC number	2005658	2005659	2005660	2027760

[a] Disorder of one ethyl group over two sites C11/C12 (74:26). Disorder of the second anion over two sites (58:42). The C_2F_5 groups were refined as rigid groups, using a well-defined fragment. [b] Disorder of Si2 and two C_2F_5 groups over two sites (80:20). [c] Disorder of F4, F5 over two sites (0.74:0.26). Disorder of F11 to F15, C25 over two sites (0.69:0.31). [d] H1 was refined isotropically.

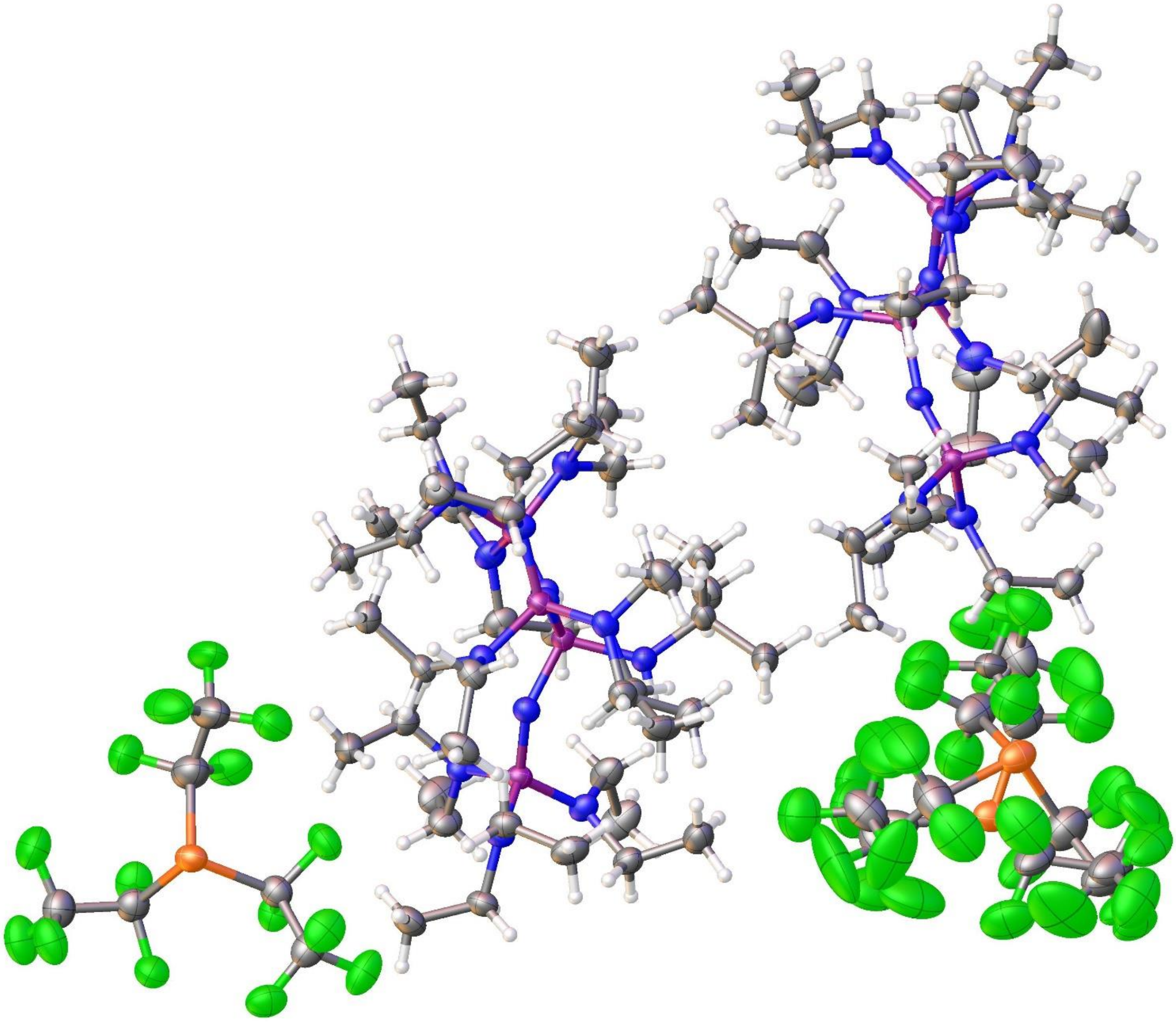

Figure S31. Plot of the molecular structure (asymmetric unit) of $[EtP_4H][Si(C_2F_5)_3]$.

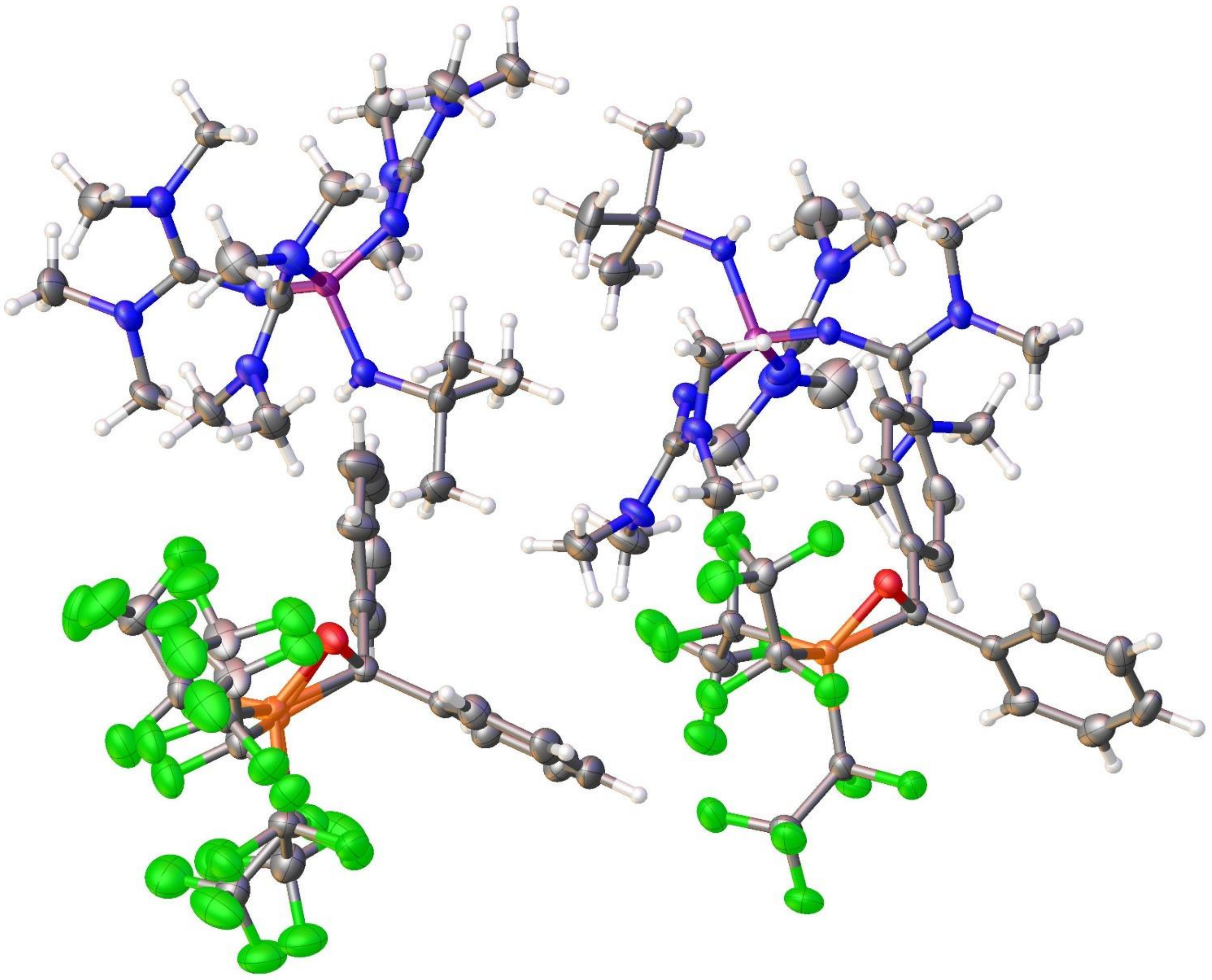

Figure S32. Plot of the molecular structure (asymmetric unit) of [tmgP$_1$H][Si(C$_2$F$_5$)$_3$(η^2-CPh$_2$O)].

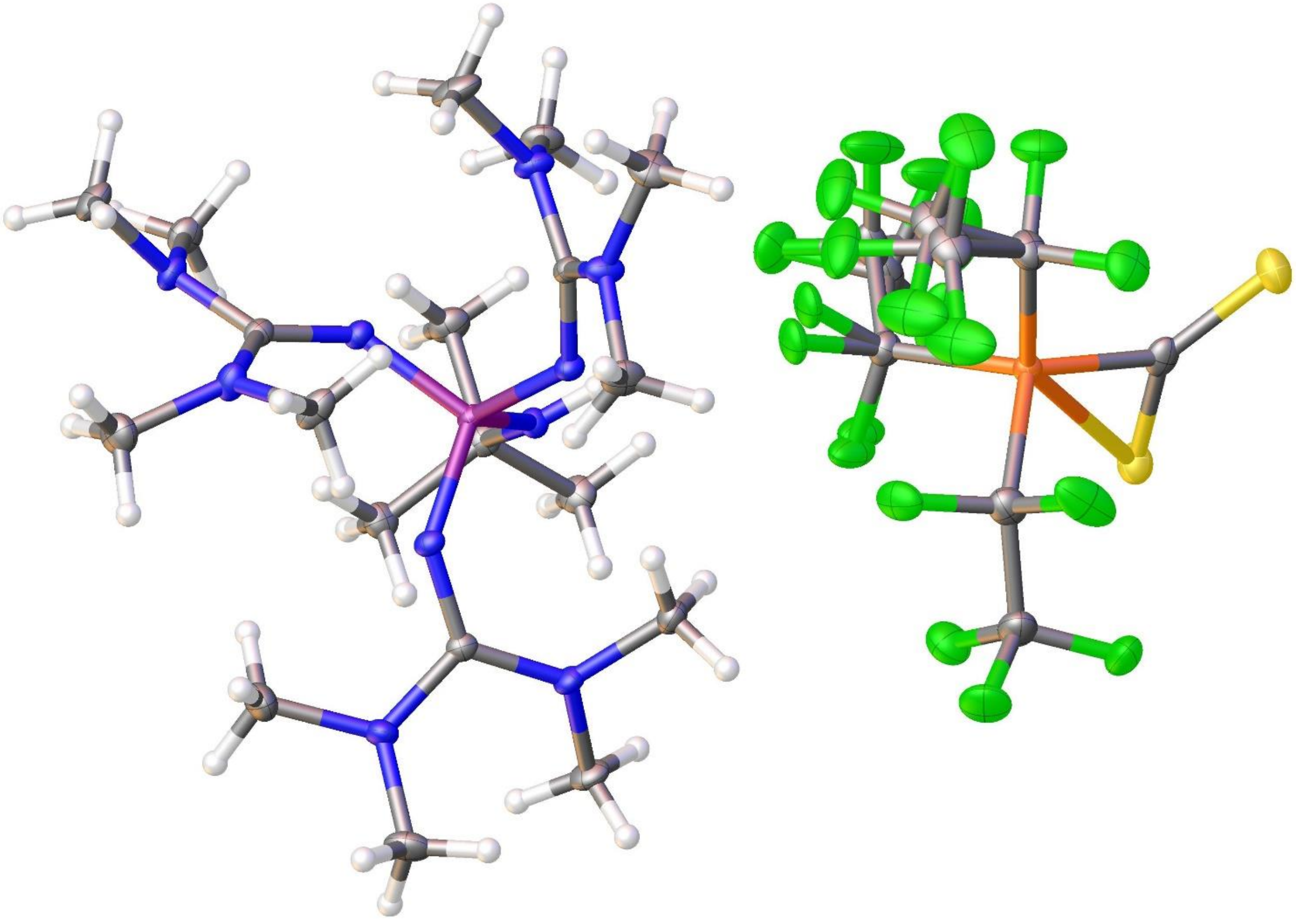

Figure S33. Plot of the molecular structure (asymmetric unit) of [tmgP$_1$H][Si(C$_2$F$_5$)$_3$(η^2-CS$_2$)].

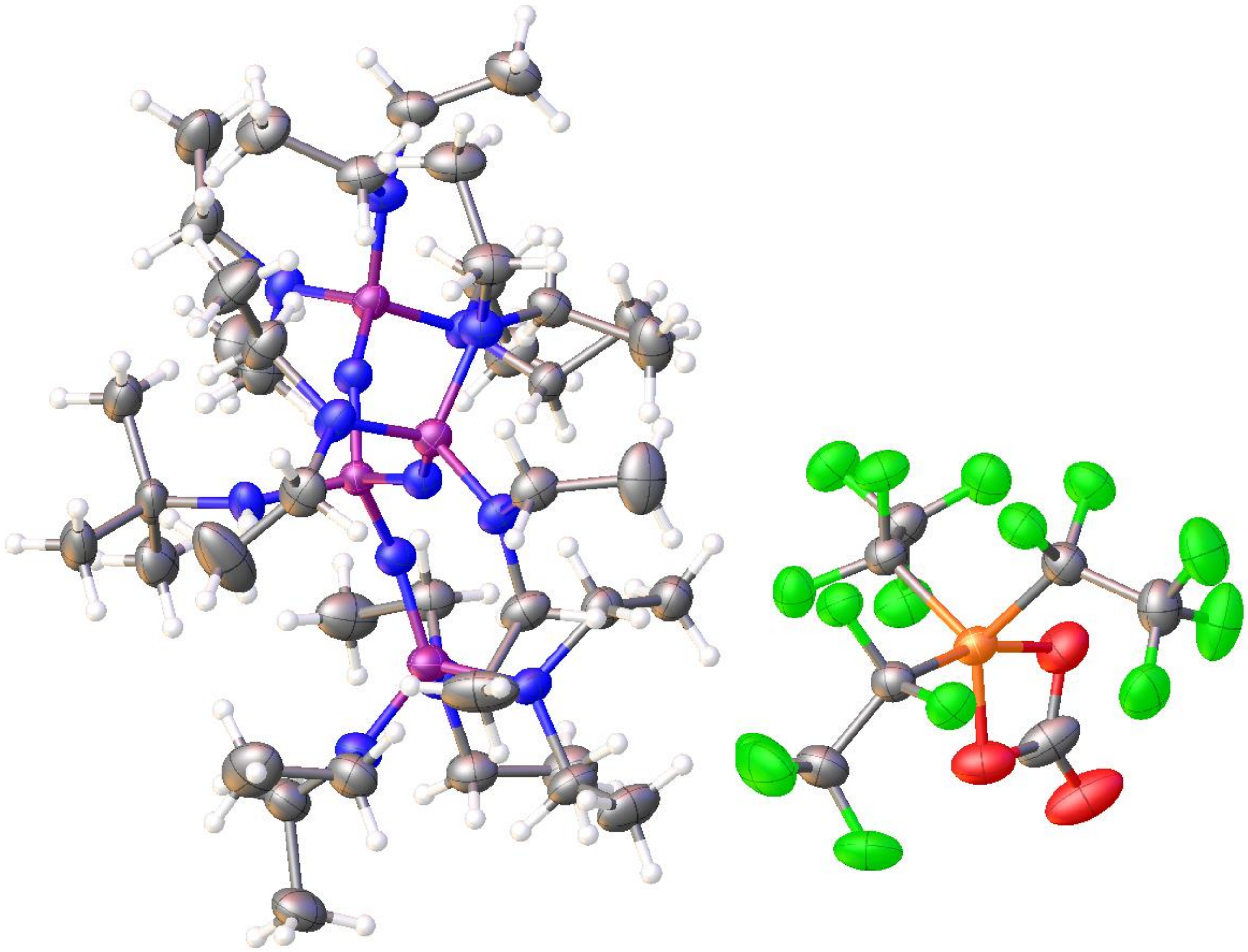

Figure S34. Plot of the molecular structure (asymmetric unit) of [EtP_4H][$Si(C_2F_5)_3(\eta^2\text{-}CO_3)$].

References

[1] M. J. Frisch, G. W. Trucks, H. B. Schlegel, G. E. Scuseria, M. A. Robb, J. R. Cheeseman, G. Scalmani, V. Barone, B. Mennucci, G. A. Petersson, H. Nakatsuji, M. Caricato, X. Li, H. P. Hratchian, A. F. Izmaylov, J. Bloino, G. Zheng, J. L. Sonnenberg, M. Hada, M. Ehara, K. Toyota, R. Fukuda, J. Hasegawa, M. Ishida, T. Nakajima, Y. Honda, O. Kitao, H. Nakai, T. Vreven, J. A. Montgomery, Jr., J. E. Peralta, F. Ogliaro, M. Bearpark, J. J. Heyd, E. Brothers, K. N. Kudin, V. N. Staroverov, T. Keith, R. Kobayashi, J. Normand, K. Raghavachari, A. Rendell, J. C. Burant, S. S. Iyengar, J. Tomasi, M. Cossi, N. Rega, J. M. Millam, M. Klene, J. E. Knox, J. B. Cross, V. Bakken, C. Adamo, J. Jaramillo, R. Gomperts, R. E. Stratmann, O. Yazyev, A. J. Austin, R. Cammi, C. Pomelli, J. W. Ochterski, R. L. Martin, K. Morokuma, V. G. Zakrzewski, G. A. Voth, P. Salvador, J. J. Dannenberg, S. Dapprich, A. D. Daniels, O. Farkas, J. B. Foresman, J. V. Ortiz, J. Cioslowski, D. J. Fox, *Gaussian 09, Revision D.01*, Gaussian, Inc., Willingford CT, **2013**.

[2] E.D. Glendining, J. K. Badenhoop, A. E. Reed, J. E. Carpenter, J. A. Bohmann, C. M. Morales, C. R. Landis, F. Weinhold, *NBO 6.0*, Theoretical Chemistry Institute, University of Wisconsin, Madison, WI, **2013**.

[3] O. V. Dolomanov, L. J. Bourhis, R. J. Gildea, J. A. K. Howard, H. Puschmann, *J. Appl. Cryst.* **2009**, *42*, 339.

[4] G. M. Sheldrick, *Acta Cryst.* **2015**, *A71*, 3.

[5] G. M. Sheldrick, *Acta Cryst.* **2015**, *C71*, 3.
